# Light-Induced Polymeric Frustrated Radical Pairs as Building Blocks for Materials and Photocatalysts

**DOI:** 10.1021/jacs.3c09075

**Published:** 2023-10-27

**Authors:** Meng Wang, Muralidharan Shanmugam, Eric J. L. McInnes, Michael P. Shaver

**Affiliations:** † Department of Materials, School of Natural Sciences, University of Manchester, Manchester M13 9PL, U.K.; ‡ Sustainable Materials Innovation Hub, Henry Royce Institute, University of Manchester, Manchester M13 9PL, U.K.; § Photon Science Institute, Department of Chemistry, The University of Manchester, Manchester M13 9PL, U.K.

## Abstract

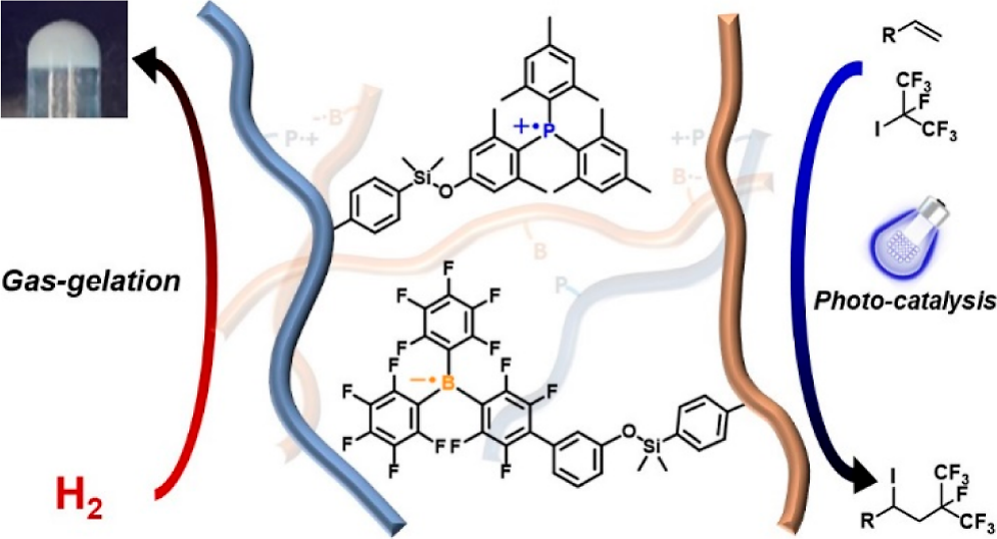

Polymeric frustrated Lewis pairs, or poly(FLP)s, have served to bridge the gap between functional polymer science and main group catalysis, pairing the uniqueness of sterically frustrated Lewis acids and bases with a polymer scaffold to create self-healing gels and recyclable catalysts. However, their utilization in radical chemistry is unprecedented. In this paper, we disclose the synthesis of polymeric frustrated radical pairs, or poly(FRP)s, by in situ photoinduction of FLP moieties, where their Lewis acidic and basic centers are tuned to promote single electron transfer (SET). Through systematic manipulation of the chemical structure, we demonstrate that inclusion of ortho-methyl groups on phosphine monomers is crucial to enable SET. The generation of radicals is evidenced by monitoring the stable polymeric phosphine radical cations via UV/vis and EPR spectroscopy. These new poly(FRP)s enable both catalytic hydrogenation and radical-mediated photocatalytic perfluoroalkylations. These polymeric radical systems open new avenues to design novel functional polymers for catalysis and photoelectrical chemistry.

## Introduction

Classical Lewis pairs (CLPs) are stable adducts formed from an electron acceptor, or Lewis acid (LA), and an electron donor, or Lewis base (LB).^1^ CLPs are widely adopted across main group, organic, and transition metal chemistry, but are transformed in their function by increasing steric encumbrance and modulating their electronics to enhance their small molecule reactivity.^1−3^ These “frustrated” Lewis pairs (FLPs) offer a sustainable alternative to conventional transition metal catalysts in a multitude of chemical processes,^1−4^ including the reversible cleavage of dihydrogen^3,5^ to support metal-free hydrogenation catalysis of imines, enamines, silyl enol ethers and more.^6^ Activation of other small molecules (CO_2_, CO, SO_2_, NO, N_2_O, alkenes, alkynes, isocyanates) has also enabled catalytic transformation into useful chemical feedstocks and polymers.^7−11^

Inspired by advances in small-molecule FLP chemistry, our team developed the inaugural poly(FLP) by incorporating pendant triaryl-borane and phosphine groups of moderate Lewis strength into polystyrenes and polyacrylates.^12,13^ Triggered gelation upon addition of small molecules gave temperature responsive and self-healing gels.^12,13^ Subsequent systems incorporated cyclohexyl-bis(pentafluorophenyl)-borane or perfluorinated-triaryl borinate ester groups to manipulate gel strength and bonding by changing borane Lewis acidity.^14,15^ These polymers can serve as efficient and recyclable catalysts for the formation of cyclic carbonates through coupling of CO_2_ and cyclic ethers.^16^ The work has inspired other poly(FLP) systems including masked polystyrene-based FLPs for catalytic C–H borylation^17^ and three-dimensional phosphine networks with exogenous tris(pentafluorophenyl)borane (B(C_6_F_5_)_3_) for dihydrogen cleavage.^18^ Transformations of CO_2_/H_2_ by polymer-supported FLPs have also been documented.^19−21^

**Scheme 1:**
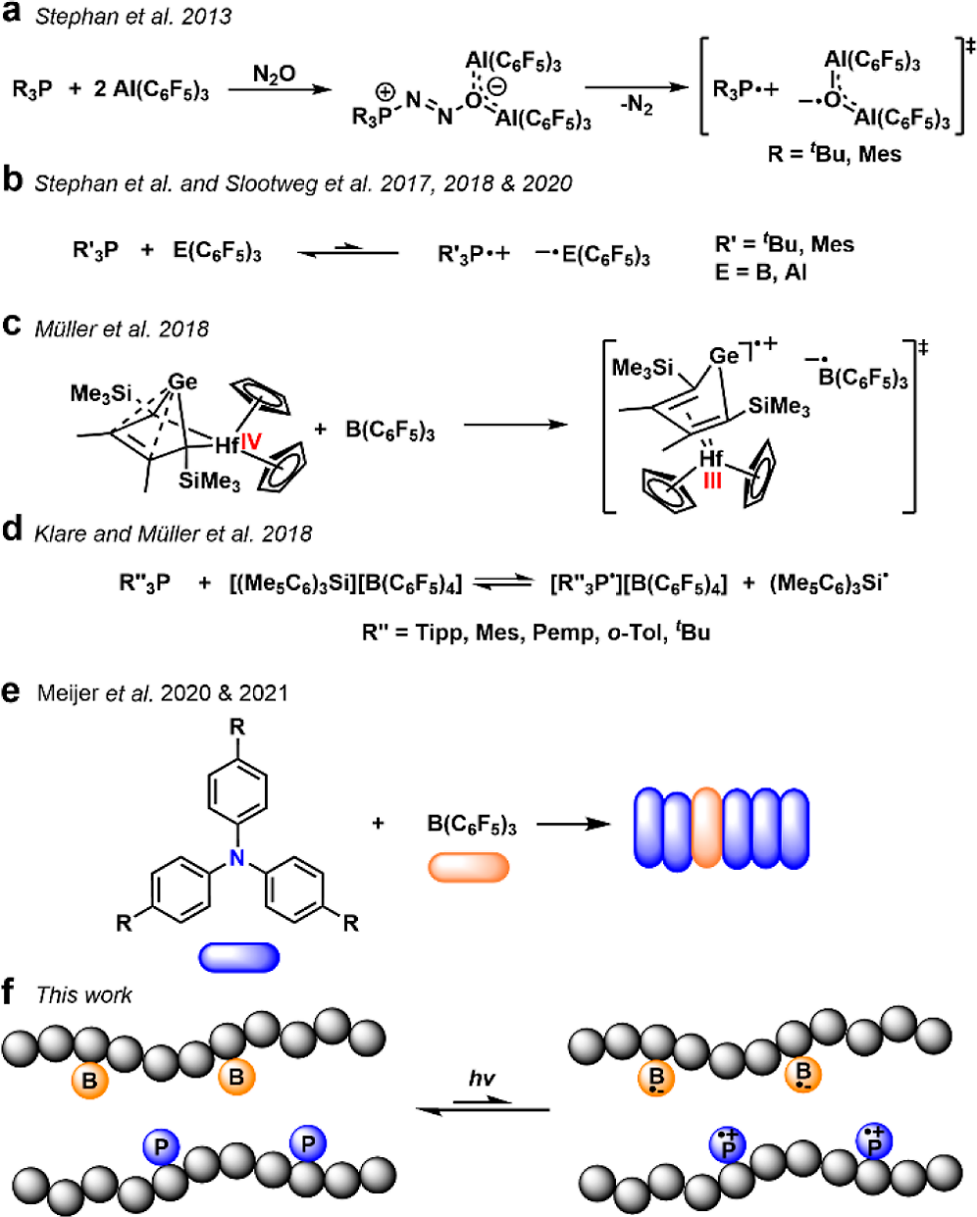
Frustrated Radical Pairs Including (a) Phosphorus and Aluminum Oxide Radical Pairs in N_2_O Activation; (b) Reversible Formation of Radical Pairs in P/Perfluoro-B Systems; (c) Hf/Ge SET Reaction with B(C_6_F_5_)_3_ to form Radical Pair Intermediates; (d) Reversible Formation of Radical Pairs between Phosphine LB and Silicon LA; (e) Supramolecular Assembly of Small Molecular Amine and Borane; (f) Transformation of Poly(FLP) to Poly(FRP) by Light-Irradiation

These discoveries highlight the versatility and advantages of polymeric FLPs as recyclable catalysts and functional materials. However, the substrate scope of poly(FLPs) is limited to polar unsaturated functionalities. This restriction is imposed by an FLP activation mechanism that inherently requires the transfer of a pair of electrons. The advent of single electron transfer (SET) in FLP systems presents a promising opportunity to break down this barrier.^22^ In 2013, Stephan et al. reported the formation of frustrated radical pairs from nitrous oxide, tris(pentafluorophenyl)aluminum and *tert*-butylphosphine (Scheme 1a); the authors demonstrated the activation of challenging C–H bonds.^23^ That same year, Wang et al. also reported single electron oxidation of a methylene-bridged triphenylamine by B(C_6_F_5_)_3_.^24^ In later studies, phosphine-coordinated boryl radicals were developed and characterized,^25^ including the observation of a Lewis basic radical cation (R_3_P•+) by Stephan et al.^26−28^ and the [B(C_6_F_5_)_3_]•– radical anion by Slootweg et al. (Scheme 1b).^29−31^ Müller et al. also spectroscopically observed the SET between B(C_6_F_5_)_3_ and bespoke LBs (Scheme 1c,d).^32,33^ This fundamental understanding has recently been extended to radical-assisted catalysis.^34−36^ Besides these findings, Meijer et al. also extended FRP into supramolecular chain structure by small molecular boranes and amines, with photoinduced SET between LA and LB observed (Scheme 1e).^37,38^ Most recently, Lin et al. reported the FRP-mediated regioselective C–H functionalization using the neutral [HMDS^•^/HPDS^•^/^*t*^BuO^•^] [TEMPO^•^] pair.^39,40^

As stable polymeric radicals are potentially transformative for both photoelectrochemistry and functional material science, we speculated that light-induced FRP-based radicals could be translated to polymeric systems. Herein, we report a light-induced polymeric frustrated radical pair, poly(FRP), the first built from macromolecular FLP. We demonstrate how small differences in chemical structure and light can affect the radical reactivity and extend the use of these systems to conventional unsaturated bond activation and radical-based catalysis (Scheme 1f).

## Results and Discussion

### Synthesis of LA

We hypothesized that increased Lewis acidity was key to a polymeric SET system. To target the required high Lewis acidity, we attempted to incorporate vinyl-functionalized B(C_6_F_5_)_3_ functionalities into polymer chains via radical polymerization based on a published procedure.^20^ We observed that highly Lewis acidic boron atoms complexed with various polymerization reagents, including sulfur-containing reversible addition–fragmentation chain-transfer agents, nitrile and oxygen-functionalized initiators in free radical polymerizations, TEMPO derivatives in nitroxide-mediated polymerizations, and ligands in atom transfer radical polymerizations (ATRP), quenching the reactivity of the resultant copolymers. Unable to reproduce this work, we shifted our synthetic strategy to post-polymerization modification, which has proven successful for other Lewis acidic polymer systems.^14,15,41^ This methodology had the added benefit of distributing the functionality more evenly across the polymer chain, as first generation systems were gradient copolymers.

**Figure 1:**
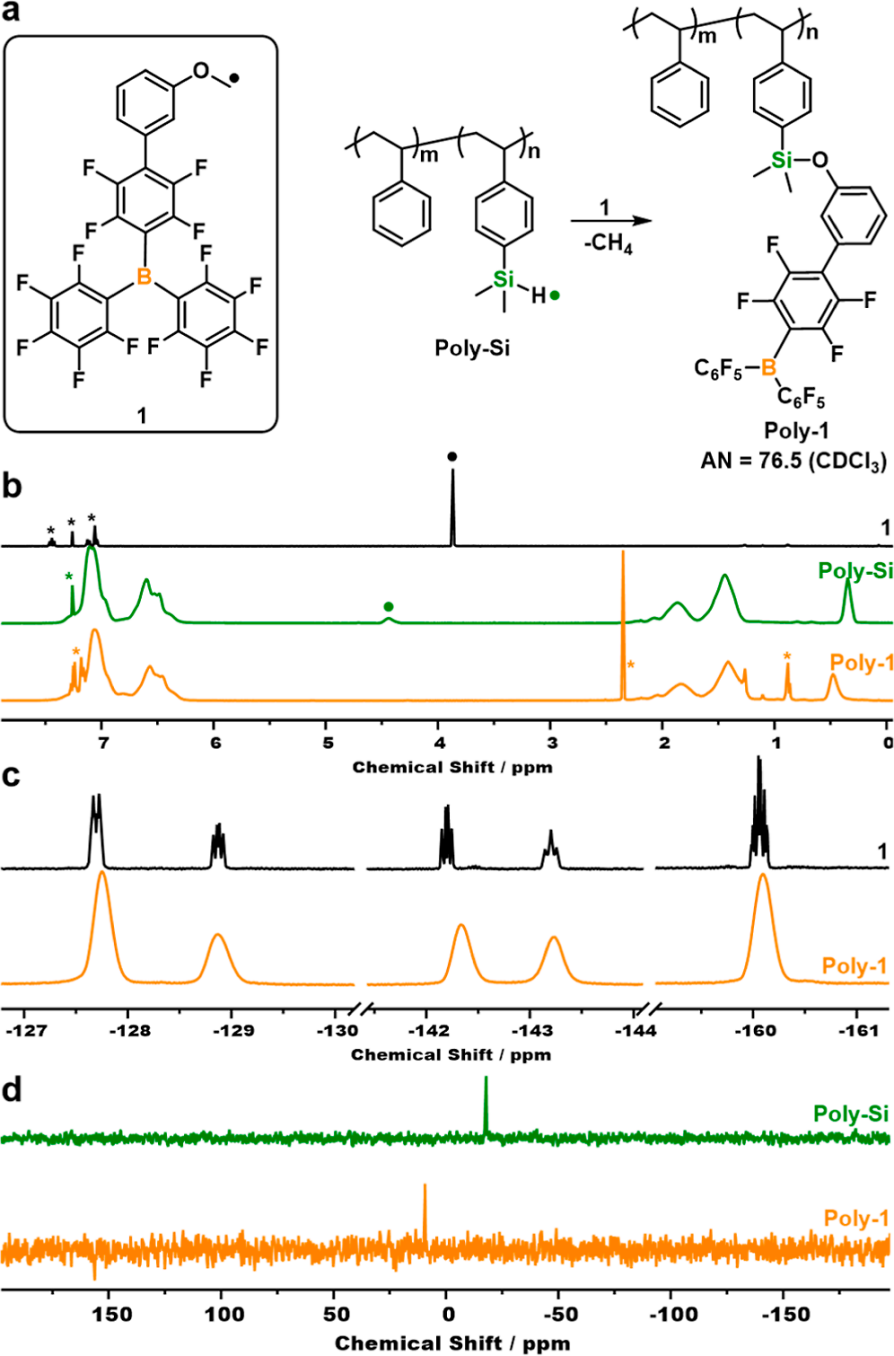
(a) Schematic representation of the reaction between **1** and **Poly-Si** to form **Poly-1**. The Lewis acidity (AN = acceptor number) shown was determined by the Gutmann–Beckett scale. (b) Stacked ^1^H, (c) ^19^F, and (d) ^29^Si DEPT90 NMR spectra before and after decoration of **Poly-Si** with **1**. *solvent peaks.

To preserve the integrity of the boron moieties, a highly efficient and clean reaction between the boron precursor and the parent polymers is needed. Catalytic reduction of silanes and anisoles, to yield siloxanes and inert methane gas as the sole byproduct was promising.^42,43^ Piers et al. utilized this reaction to attach an anisole-modified boron molecule **1** to dendrimers.^44^ We thus developed a route to append **1** to poly(4-vinylphenyldimethylsilane-*co*-styrene) (**poly-Si**) to produce the desired linear Lewis acidic copolymers (Figure 1a). The methodology for copolymerization of styrene with 4-vinylphenyl-dimethylsilane is key, as it is crucial to avoid any potential complexation between the end-groups of the resultant **poly-Si** copolymer with boron functionalities. ATRP yields halide end-groups and was therefore a promising first choice. Rare reports of FLP-mediated alkyl halide activation^45,46^ suggest potential dehydrohalogenation could effectively quench both the LAs and the LBs. Indeed, we observed these side reactions when testing **1/3** with 1-(chloroethyl)benzene or 1-(bromoethyl)benzene, as evidenced by ^1^H nuclear magnetic resonance (NMR) spectroscopy (Supporting Information SYN.12, Figures S42–S49). We pivoted to using anionic polymerization to produce the desired **Poly-Si** (5 mol % loading of Si moieties). Tetrahydrofuran is the preferred solvent for this polymerization, which produces well-defined polymers and monomodal molecular weight distributions when reactions are carried out at a low temperature (−78 °C).

Post-polymerization modification of **Poly-Si** was performed by slow addition of **1** at ambient temperature (Figure 1a) to control rapid gas evolution. The resultant **Poly-1** was purified via precipitation into hexane with the composition confirmed by NMR spectroscopy, including the disappearance of silane protons and anisole-methyl groups (Figure 1b), broadening of ^19^F NMR resonances (Figure 1c) and a downward shift of the lone ^29^Si NMR signal, indicating the formation of siloxanes (Figure 1d). The low loading of boron moieties in **Poly-1** (5 mol %) prevented detection of peaks in the ^11^B NMR spectrum, similar to other reports of polymeric LAs.^15,47^ The presence of boron centers was instead confirmed using ^31^P NMR spectroscopy and the Gutmann–Beckett method, with a resultant acceptor number of 76.5 in CDCl_3_ (Supporting Information SYN10–11, Figures S35–S41), which remained unchanged to **1** and both similar to the most widely used small molecular B(C_6_F_5_)_3_ (AN = 81.8 CDCl_3_).^48^ Taken together, these analyses confirm a single Lewis acidic boron environment in **Poly-1** and thus a clean and quantitative reduction reaction.

Thermogravimetric analysis further corroborated the composition of **Poly-1**, revealing three stages of mass loss upon heating under air in contrast to precursor polymer **Poly-Si**. For **Poly-1**, the first two events (at 110 and 200 °C) were attributed to the sequential hydrolysis and loss of the two perfluoroaromatic rings on boron, as the observed mass loss over each event (8.3 and 16.0%) were in reasonable agreement with the theoretical values (6.1 and 12.3% for the sequential loss of the two C_6_F_5_ rings; see Supporting Information Figure S70). This degradation precluded more thermal characterization; attempts to use differential scanning calorimetry to measure the glass transition temperature (*T*_g_) of **Poly-1** were unsuccessful.

### LA/LB Screening for FRP–SET

**Figure 2:**
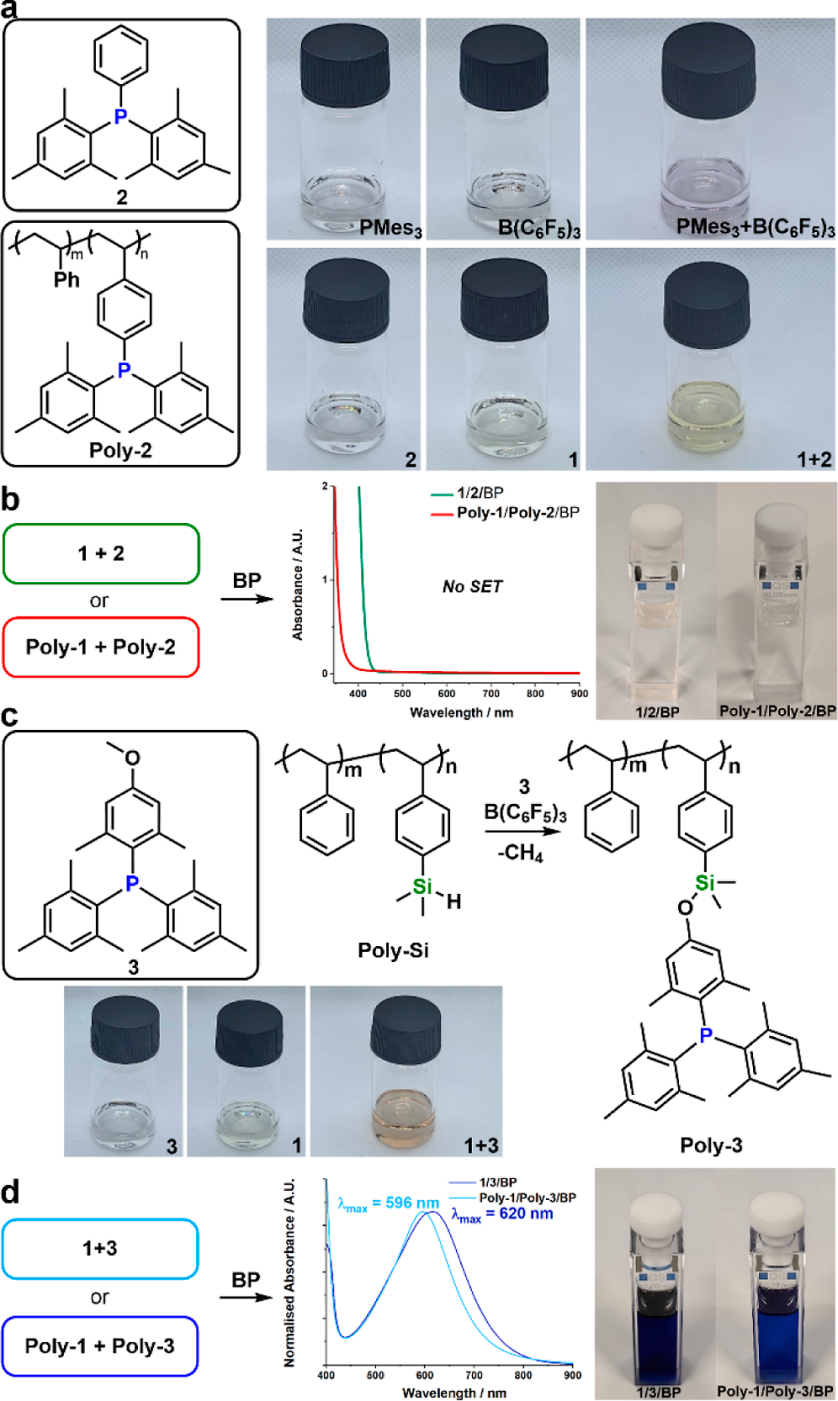
(a). Photographs of solution mixtures of PMes_3_/B(C_6_F_5_)_3_, **1/2**, and **1/3**; addition of BP into the (b) mixture of **1** and **2** or **Poly-1** and **Poly-2** and the (c) mixture of **1** and **3** or **Poly-1** and **Poly-3** in toluene and their corresponding UV/vis spectra.

Previously reported small molecule LBs and acids that can induce SET normally appear as a pale purple color solution upon mixing at ambient conditions, indicating the formation of electron–donor–acceptor (EDA) complexes, confirmed by Slootweg et al. (reproduced in Figure 2a).^26−28,30^ Addition of benzoyl peroxide (BP) into this mixture allowed trapping of phosphine radical cations, which are ^31^P NMR silent but can be probed by UV/vis spectroscopy. However, the boron radicals will be quickly consumed by BP radicals to form dormant tetracoordinated boryl anion, confirmed by ^11^B NMR spectra. In the case of our system, mixing dimesitylphenylphosphine **2** with **1** or their polymeric variants instead gave a pale-yellow solution (Figure 2a). UV/vis and electron paramagnetic resonance (EPR) spectroscopy did not support the formation of any EDA or radical species, including when the radical sensing molecule BP was added (Figure 2b). Additionally, both the ^11^B and ^31^P NMR spectra of the mixtures remained unchanged, suggesting that thermal-induced SET is highly unlikely to occur under ambient conditions. The strong π-stacking interactions among the aromatic rings of the planar molecules is instead a likely origin for the color, rather than the inhibited SET process.

We hypothesized that the additional *ortho*-positioned methyl groups in trimesitylphosphine (PMes_3_) may be essential for the thermal-SET process. We therefore synthesized an analogous phosphine compound **3**, which contains both the additional *ortho*-substituents as well as a methoxy group to graft onto **Poly-Si** analogously to **1** (Figure 2c). The substitution of the styryl group with 2,5-dimethyl-4-methoxybenzene to afford **3** is straightforward (Supporting Information SYN.8). The reduction reaction between **3** and **Poly-Si** is facilitated by adding 5 mol % of B(C_6_F_5_)_3_, which is removed by repeated precipitation during isolation of the product (**Poly-3**).

The mixing of **1** and **3** in toluene gave an intense orange-brown color (Figure 2c), indicating a potential formation of EDA species. BP was added to confirm if **1** and **3** can undergo radical reaction with it; indeed, this triggered an immediate change to a deep blue color (Figure 2d and Supporting Information SYN.13). UV/vis spectroscopy confirmed the formation of the radical cation of **3** by a strong absorbance band with λ_max_ = 620 nm (Figure 2d). This band is red-shifted compared to that of the published PMes_3_ radical cation (λ_max_ = 592 nm), indicating the presence of the donating methoxy groups.

A similar radical reaction was observed on the macromolecular variants (i.e., **Poly-1**/**Poly-3**) as well. Addition of BP created a blue color and a large absorbance peak with λ_max_ = 596 nm for the polymeric phosphine radical cations (Figure 2d), blue-shifted when compared to their small-molecular counterparts. The ^31^P NMR spectra of both small molecular and macromolecular mixtures were silent, indicating the formation of paramagnetic species upon peroxide addition (Supporting Information Figures S52 and S53).

**Figure 3:**
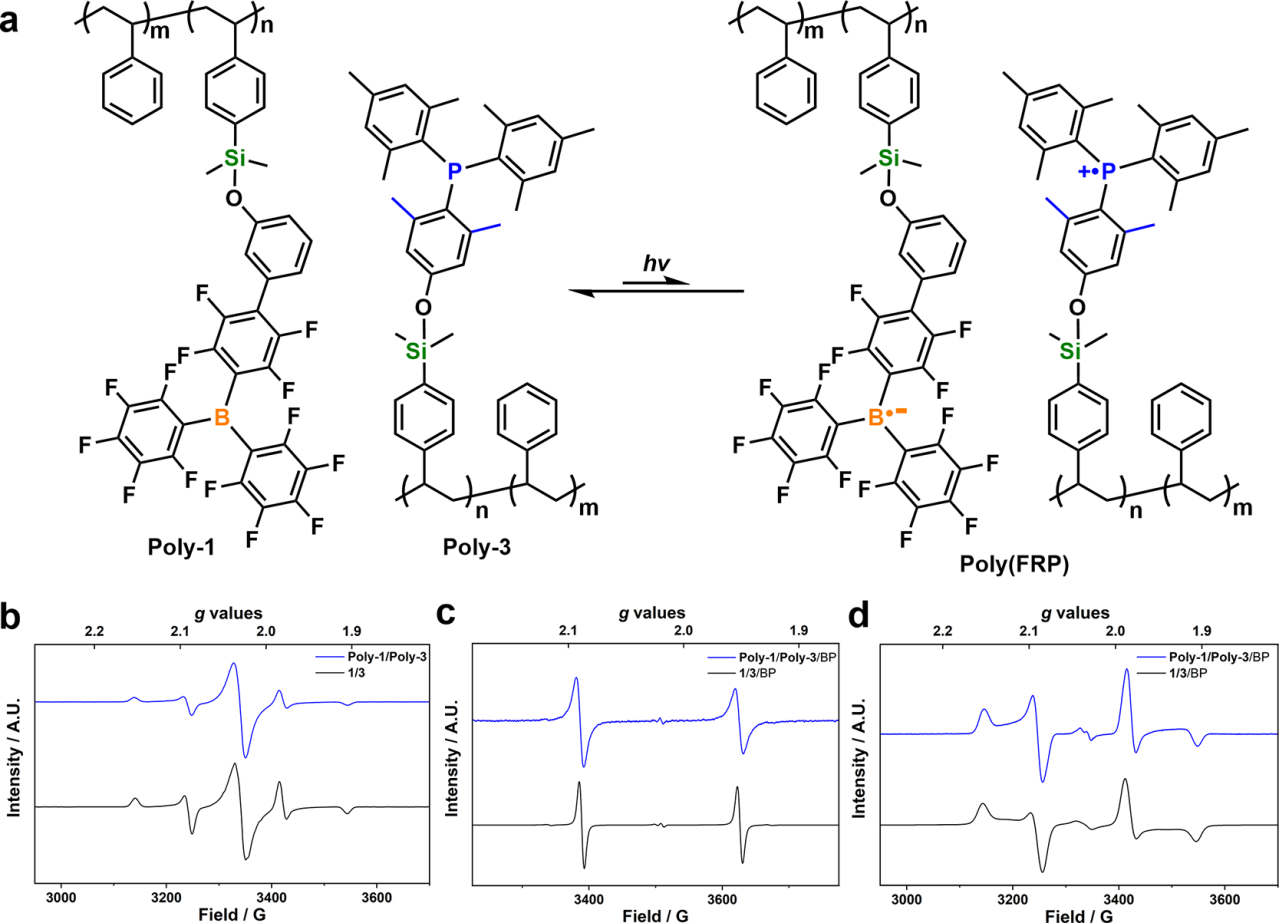
(a) Poly(FRP) triggered by light irradiation; X-band EPR spectra of **1**/**3** and **Poly-1**/**Poly-3** (b) at 20K; (c) at RT in the presence of BP; and (d) at 20 K in the presence of BP.

To further validate the formation of poly(FRPs) upon photoinduced SET, continuous-wave X-band EPR spectroscopy was used to compare to published P^*t*^Bu_3_/PMes_3_ and B(C_6_F_5_)_3_ SET processes.^30^ The frozen solution EPR spectra of mixtures of **1/3** and **Poly-1/Poly-3** in toluene clearly showed radical signals (Figure 3b and Supporting Information S78–S80). Under blue light-emitting diode (LED) irradiation (λ_max_ = 455 nm) at 20 K, a color change from orange-brown to blue was observed for both small-molecular and polymeric systems, indicating the formation of radical pairs. The boron radical anion formed showed a broad, intense peak at center (*g* = 2.0042 for small-molecular FRP, and *g* = 2.0050 for polymeric FRP), while the cationic phosphorus radical showed four lines [centered at *g*_iso_ = 2.0042 | *g* = (2.0020, 2.0044, 2.0062) for **1**/**3** and *g*_iso_ = 2.0042 | *g* = (2.0020, 2.0046, 2.0061) for **Poly-1**/**Poly-3**] due to the hyperfine coupling [*A*_iso_ = 698 MHz | *A* = (1130, 477, 488) MHz for **1**/**3** and *A*_iso_ = 702 MHz | *A* = (1137, 473, 495) MHz for **Poly-1**/**Poly-3**] with the spin-half ^31^P nucleus. Both the *g*-factors and coupling tensors are similar in value to the reported analogues in the literature, and the simulated spectra fit well with the experimental data (Supporting Information Figures S78–S80).

Remarkably, the radical species for the polymeric system were much longer lived than those of the small molecules. The relative decay in blue color upon removal of irradiation and cooling was instantaneous for small-molecule pair **1/3**, whereas the equivalent process took ca. 6 min for **Poly-1/Poly-3**. The reduced diffusion rate of FRP species tethered to macromolecular backbones may enable this important feature; however, the details of this mechanism still needs investigation. For both small-molecular and macromolecular FRPs, light-induced SET can be observed up to 200 K, although the signal intensity decreases quicker at this temperature.

**Poly-1/Poly-3** does not show any response in isotropic EPR spectroscopy due to the short lifetime of induced radicals at ambient temperature. We thus further probed the phosphine radicals by the addition of BP. Doublet splitting resonances were observed for **1/3** and **Poly-1/Poly-3** (Figure 3c, Supporting Information S81), confirming the presence of phosphorus radical cations. The experimental results (*g* = 2.0050, A_iso_ = 666 MHz for **1**/**3** and *g* = 2.0051, A_iso_ = 670 MHz for **Poly-1**/**Poly-3**) were in good agreement with the simulated results (Supporting Information Figures S82 and S83) and consistent with previous small-molecule reports.^27^ The boron radical is of course quenched with the peroxide addition, precluding observation. In the case of small-molecular FRPs, additional minor peaks corresponding to phosphine dimer and semiradical on BP were detected (2.5 and 0.3% of total phosphine radicals). Previous reports suggest that phosphorus dimer radicals can be suppressed with increased steric hindrance,^33^ which is indeed achieved in our macromolecular FRP system. Notably, slower radical formation was observed in poly(FRP)s compared to their small-molecular counterparts potentially due to slower diffusion of radical scavenger through the viscous polymer solution (Supporting Information Figure S1). The resonances for polymer systems were broadened, supporting the localization of radicals on polymers. Frozen solution EPR spectra at 20K for these mixtures were also probed (Figure 3d, Supporting Information S84–S86). The boron and phosphorus dimer radicals again were not observed for **Poly-1/Poly-3**/BP. The Hamiltonian parameters of phosphine radicals were determined [*g* = (2.0024, 2.0049, 2.0070) *A* = (1121, 456, 476) MHz for **1**/**3** and *g* = (2.0012, 2.0041, 2.0067) *A* = (1126, 451, 477) MHz for **Poly-1**/**Poly-3**]. The *A*_iso_ of the mixture (∼684 MHz) also showed reasonable agreement with the coupling constant extracted from the liquid-state spectra. All the EPR and UV/vis analysis results demonstrate the first occurrence of SET in a poly(FRP) systems, and their ability to trigger radical reactions under ambient conditions. It should be noted that while the *ortho*-methyl groups introduced to **3** and **Poly-3** are the key factor for enabling SET, a potential role of the more flexible *para*-oxygen linkage in this system should not be excluded.

### Small-Molecule Activation by Poly(FRP)

**Figure 4:**
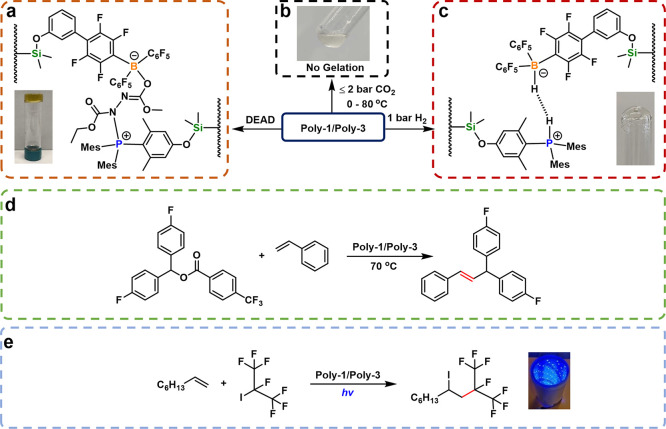
Activation reactions by **Poly-1**/**Poly-3**: (a) DEAD-triggered gelation; (b) no gas-induced gelation achieved with CO_2_ introduction under mild conditions (≤2 bar, 0–80 °C); (c) dihydrogen cleavage to form transparent gel; (d) attempted diaryl-ester/styrene coupling catalysis; and (e) alkene-perfluoroalkylation coupling reaction.

With poly(FRP)s in hand, we sought to test their catalytic reactivity (Figure 4). As observed in EPR samples, mixing concentrated toluene solutions (67.6 mM of B/P moieties) of **Poly-1** (10 mol % loading, *M*_n,th_ = 33300) and **Poly-3** (10 mol % loading, *M*_n,th_ = 29800) led to a noticeable increase in viscosity (Supporting Information Figure S2). ^31^P NMR spectra showed no change from pure **Poly-1**, indicating no substantive binding between the LA and LB moieties on the NMR timescale. Coordination of boron would also quench molecule fluorescence under long-range wavelength UV; this is also not observed. This suggests that weak and dynamic interactions between the boron and phosphorus groups may increase solution viscosity through reversible adduct formation but come short of forming a true gel.

The introduction of 1 bar of dihydrogen gas to a mixture of **Poly-1/Poly-3** (63.2 mM, each with 5 mol % loading of LA [*M*_n,th_ = 22000] or LB [*M*_n,th_ = 20,300]) induced a rapid transformation from a viscous liquid to a nontransparent solid (Figure 4c and Supporting Information S3). This gelation was accompanied by a downfield shift of the broad resonance in ^31^P NMR spectra (from −37.1 to −28.0 ppm) with concomitant P–H coupling (1*J*_P–H_ = 485 Hz), confirming the direct attachment of the protons to the phosphorus centers (83% conversion, Supporting Information Figure S58). Although the low boron loadings and broadening of the polymeric resonances make it challenging to directly probe B–H formation in the polymeric system, NMR analyses of dihydrogen cleavage using small-molecule analogues **1** and **3** corroborated B–H formation. A conspicuous upfield shift of the ^11^B NMR peak (from +59.7 to −25.1 ppm) and the emergence of B–H coupling (^1^*J*_B–H_ = 91 Hz) (Supporting Information Figure S55) is matched to ^31^P NMR trends consistent with those observed from the macromolecular counterparts (Supporting Information Figure S56). Catalytic hydrogenation of *N*-benzylidene *tert*-butylamine—a classic test for FLP systems—gave quantitative conversion to products using **Poly-1/Poly-3** (Supporting Information SNY.19 and Figure S68). This agrees well with the efficacy of the previously reported PMes_3_/B(C_6_F_5_)_3_ FRP^49^ and indeed our small-molecule mimic **1/3**.

The subtle reactivity of this extends to responsive gelation, especially when comparing the reactivity of cross-linkers diethyl azodicarboxylate (DEAD) and propylene oxide in **Poly-1/Poly-2** (5 mol % LB, *M*_n,th_ = 19 000) to **Poly-1/Poly-3** (63.2 mM in toluene, 5 mol % LA/LB)(Supporting Information SYN.16 and Figures S4–S7). We observed immediate gelation upon the addition of DEAD when using the less bulky **Poly-2** as the LB, whereas DEAD-triggered gelation was significantly delayed with **Poly-3**. For the **Poly-1/Poly-3** pair, the initially dark green solution transitioned into an orange-colored gel over the course of 3 h. This weak gel broke easily when swollen with solvent. Substituting DEAD with propylene oxide, a substrate that requires the proximity of both LA and LB for activation, did not form a stable gel. These findings suggest that the enabling switch to SET behavior via the extra *ortho*-methyl groups around phosphorus in **Poly-3** also controls the selectivity of gelators as functional material building blocks.

No CO_2_ activation by **Poly-1**/**Poly-2** or **Poly-1/Poly-3** to form free-standing gels occurred; no visual or NMR evidence of direct carbon capture was observed up to 2 bar of CO_2_ pressures and temperatures from 0 to 80 °C (Figure 4b). This is not surprising as no CO_2_ activation under mild conditions by small-molecular PMes_3_/B(C_6_F_5_)_3_ has ever been reported, despite published polymeric systems with similar Lewis acidity and basicity suggesting strong binding.^21,50,51^ We have not been able to reproduce these findings and note that CO_2_ capture in polymeric FLPs should be more challenging than that for analogous small molecules due to the relative entropic penalties in both cases.

### Radical Reactivity by Poly(FRP)

Small-molecule FRPs can trigger homolytic cleavage of C–O bonds to generate carbon radicals, which can then undergo addition reactions with unsaturated substrates.^35^ We tested our poly(FRP) systems in the reaction of bis(4-fluorophenyl)methyl 4-(trifluoro-methyl)benzoate and styrene (Figure 4d). Small-molecule FRP mimics **1**/**3** gave 41% conversion of alkene (70 °C, 7 h, Supporting Information SYN.17), while **Poly-1**/**Poly-3** gave a disappointing 10.3% conversion. No significant improvement was observed when tuning reaction conditions or substrate order of addition (Supporting Information SYN.17, Table S1 Entries 1–5, and Supporting Information Figure S59 and 60). A significant increase in solution viscosity was noted as the reaction progress, suggesting potential competing cross-linking reactions. Indeed, only 65% of polymeric phosphine was converted into its final protonated form, compared to 94% conversion when using small molecules. ^11^B NMR spectroscopy supported benzoate binding to boron (Supporting Information Figure S61), suggesting that cross-linking may lower the catalytic efficiency. This contrasts with the hydrogenation catalysis (vide supra) or our previously reported CO_2_–cyclic ether coupling^16^ where the efficient product release avoids unwanted cross-linking.

In seeking other SET catalytic transformations, we revisited the aforementioned reactivity between alkyl halides and our FRPs. Though this was previously an undesired side reaction to ATRP-mediated polymer synthesis, we thought it could be leveraged for perfluoroalkylations of alkenes, a crucial process in the chemical industry. There is some precedent for this, as Czekelius et al. demonstrated FLP-mediated alkene-perfluoroalkylations using a more Lewis basic tris(*tert*-butyl)phosphine at ambient temperature,^36^ although it was unclear if it involved a radical mechanism. We explored the catalytic conversion of 2-iodoheptafluoropropane and 1-octene in toluene using **Poly-1/Poly-3** systems (Figure 4e and Supporting Information Table S2). The reactions are promoted by both heat and light. Using blue LED irradiation and 5 mol % **Poly-1/Poly-3** gave 95.3% conversion in 24 h (Supporting Information Table S2 Entry 14). Small-molecule systems are faster, which was expected due to the slower molecular diffusion in the viscous polymer solution (Supporting Information Table S2 entries 3 and 14 and entries 2 and 13). The type of light source significantly affects the efficiency of catalysis: blue light yielded the highest conversion, green yielded intermediate conversions, and red light gave very little conversion (Supporting Information Table S2 entries 2, 4, and 5 and entries 13, 15, and 16). Control reactions without catalyst or without light irradiation each resulted in negligible conversions (Supporting Information Table S2 entries 6 and 11), further evidencing the importance of both light and our SET system for successful perfluoroalkylation.

Mediation by light suggests a radical reaction. Indeed, we can shut down reactivity with a radical trap (hydroquinone or TEMPO, 0% conversion, Supporting Information Table S2 entries 7 and 8), supporting our hypothesis that radicals are the effective intermediate species for this catalytic transformation. Although **Poly-3** alone can effect the reaction (37.4%), cocatalyst **Poly-1** significantly improves turnovers (Supporting Information Table S2 entries 13, 17, and 18). This was confirmed by EPR spectroscopy, where a significant drop in line intensity of boron radicals at 20 K occurred when adding the reaction substrate under blue LED irradiation (Supporting Information Figures S87 and S89). When the LED irradiation was ceased, a further decrease in the relative boron radical intensity compared to the phosphine radical species was observed (Supporting Information Figures S88 and S90). These observations indicate that the alkene/perfluoroalkyl iodide quickly consumes boron radicals, making phosphine reactivity the rate-determining step. While Czekelius et al. proposed substrate coordination to boron for their system, we can now show the direct involvement of the anionic boron radical in the alkene-perfluoroalkylation catalysis (Supporting Information Scheme S1). Tested with small-molecule mimics **1/3**, the lack of change to the chemical shifts of ^11^B and ^31^P NMR peaks suggest that substrate turnover is quicker than the NMR timescale (Supporting Information Figure S66). This lability could enable poly(FRP) catalyst recycling following the recovery by precipitation into hexane. Our next cycle of catalysis showed a slight decrease in the overall reactivity (82% compared to the first catalytic cycle), likely due to degradation of the boron functionalities (note that **Poly-3** showed no degradation, as shown in Supporting Information Figure S67). Further exploring these and other reactions, including modifying polymer structures and substrates, should help to further understand and then improve the performance of these unique SET polymeric catalysts.

## Conclusions

We have successfully synthesized a highly Lewis acidic borane and a sterically hindered phosphine, incorporated them onto styrenic copolymers using postpolymerization modification, and shown that this system exhibits remarkable reactivity. The new system enables classical FLP activations for small molecules (H_2_, DEAD) but also can be extended to a light-induced SET phenomenon, as confirmed by UV/vis and EPR spectroscopy. The ortho-substituted methyl groups on the triarylphosphine are essential to these SET processes. The system catalyzes both traditional hydrogenation reactions as well as the radical-mediated perfluoroalkylation of alkenes. This latter reaction is most efficient under blue LED light irradiation, with EPR spectroscopy confirming the involvement of both boryl and phosphorus radical species in the catalytic process. We demonstrate that these polymeric catalysts can be recycled and reused, albeit with a slight decrease in activity. This light-induced SET between polymeric-based FLPs opens new doors for catalysis and FLP activation and potential applications in polymeric light sensors, light-induced conductive copolymers, and photoelectronics.

## Supporting Information Available

Experimental details, synthetic protocols, and additional characterization data (PDF)
