# Supplementary material for: Light-Induced Polymeric Frustrated Radical Pairs as Building Blocks for Materials and Photocatalysts
Source: J Am Chem Soc. 2023 Oct 27;145(44):24294–301. doi: 10.1021/jacs.3c09075 (PMC10636756; doi:10.1021/jacs.3c09075)
Supplement: Supplementary file 1 — ja3c09075_si_001.pdf [file ja3c09075_si_001.pdf]

Supplementary Information for

Light-induced Polymeric Frustrated Radical Pairs as Building Blocks for Materials and Photocatalysts.

Meng Wang[†,‡] , Muralidharan Shanmugam[§], Eric McInnes[§] and Michael P. Shaver[†,‡,*]

[†] Department of Materials, School of Natural Sciences, University of Manchester, Manchester, UK.

[‡] Sustainable Materials Innovation Hub, Henry Royce Institute, University of Manchester, Manchester, UK.

[§] Photon Science Institute, Department of Chemistry, The University of Manchester, Manchester M13 9PL, U.K.

* michael.shaver@manchester.ac.uk

Table of contents

Experimental Procedures 3
General considerations 3
Materials 3
Instrumentation 4
Synthesis of Lewis acidic copolymer Poly-1 5
SYN.1 Synthesis of 2,3,5,6-tetrafluoro-3'-methoxy-1,1'-biphenyl 5
SYN.2 Synthesis of dimethyltin bis[4-(2,3,5,6-tetrafluoro-3'-methoxy-1,1'-biphenyl)] 6
SYN.3 Synthesis of 1 6
SYN.4 Synthesis of Poly-Si 7
SYN.5 Synthesis of Poly-1 8
Synthesis of Lewis Basic Copolymer Poly-2. 9
SYN.6 Synthesis of 2 9
SYN.7 Synthesis of Poly-2 10

Synthesis of Lewis Basic Copolymer Poly-3. 10
SYN.8 Synthesis of 3 10
SYN.9 Synthesis of Poly-3 11
Gutmann-Beckett Lewis Acidity Scale Determination. 12
SYN.10 NMR data of mixture of 1 and triethylphosphine oxide 12
SYN.11 NMR data of mixture of Poly-1 and triethylphosphine oxide 12
Small molecule activation by FLP/FRP 13
SYN.12 Dehydrohalogenation of (1-haloethyl)benzene by 1 and 3. 13
SYN.13 Activation of benzoyl peroxide by 1/3 and Poly-1/Poly-3. 14
SYN.14 Dihydrogen Cleavage by 1/3. 15
SYN.15 Dihydrogen Cleavage by Poly-1/Poly-3. 16
SYN.16 Activation of DEAD and Propylene Oxide by Poly-1/Poly-2 or Poly-1/Poly-3 17
SYN.17 Diaryl-ester Activation with Styrene by FRP or Poly(FRP) 20
SYN.18 Perfluoroalkylation by FRP or Poly(FRP) 20
SYN.19 Poly-1/Poly-3 Catalyzed Hydrogenation of N-benzylidene-*tert*-butylamine 23
NMR Spectroscopic Data 24
TGA, DSC and GPC Data 55
UV/Vis Spectroscopic Data 58
EPR Spectroscopic Data 60
Reference 66

Experimental Procedures

General considerations

All air-sensitive reactions were performed under an inert atmosphere, either within an argon-filled glovebox, or using a dual-manifold Schlenk lines equipped with an in-line gas purification column containing copper catalyst unless otherwise specified. All glassware used in air-sensitive operations were pre-dried in an oven (200 ℃) overnight prior to use.

Materials

Anhydrous solvent such as hexane, toluene, tetrahydrofuran (THF), diethyl ether, and dichloroform (DCM) were collected from an mBraun Solvent Purification System (SPS-7) containing alumina and copper catalysts. Ethylene glycol dimethyl ether (DME) was dried over 4Å molecular sieves. NMR deuterated solvents such as chloroform-*d*, DCM-d_2, and toluene-d_8 were dried over calcium hydride overnight and then distilled under inert condition/vacuum. Styrene was dried over calcium hydride and collected by vacuum transfer. All solvent and reagents were degassed by three freeze-pump-thaw cycles prior to use. Tetrakis(triphenylphosphine) palladium (0) was purchased from Fluorochem and purified by recrystallization from ethanol. The following chemicals were used as received without further purification: 4-Chlorostyrene, and sodium carbonate was purchased from Fisher Scientific UK Ltd. 3-Methoxyphenylboronic acid and 3-bromo-1,2,4,5-tetrafluobenzene was purchased from Apollo Scientific Ltd. n-BuLi, tert-BuLi hexane solution, and chlorodimethylsilane were purchased from Merck Life Science UK Limited. Dimethyltin dichloride was purchased from Tokyo Chemical Industry UK Ltd. Diethyl azodicarboxylate and propylene oxide were dissolve in toluene and stored over 4Å molecular sieves. Dimesitylphosphine halide (halide = Br and Cl) was synthesized from reported literature procedures.[1-2]

Instrumentation

Nuclear magnetic reconance (NMR) data (^{1}H, ^{11}B, ^{13}C, ^{19}F, ^{29}Si, ^{31}P and ^{119}Sn) were collected using either 400 MHz or 500 MHz Bruner AVIII spectrometers equipped with either BBFO 5 mm probe or BBO 5 mm probe at 295 K unless otherwise stated. Electron paramagnetic resonance (EPR) data were collected using Bruker X-band continuous wave spectrometers, located in Photon Science Institute of Henry Royce Institute, Alan Turning Building, University of Manchester. J. Young's tap modified NMR and EPR tubes were used for air sensitive substances. The extract molecular weights of samples were determined using a Thermo Orbitrap QExactive mass spectrometer in electrospray ionization (ESI) mode. The molecular weight and polydispersity of polymer samples were determined using Agilent 1260 Infinity II Multi-detector Gel Permeation Chromatography (GPC) systems. The stationary phase of GPC was PLgel 5 micrometers columns packed with PSDVB beads. The GPC trace was collected using THF as element at a flow rate of 1 mL/min at 35 ^{o}C. The GPC system was calibrated by both conventional methods (RI detector) and universal methods (RI, LS, VS detectors) using a series of narrow dispersing polystyrene standards. Glass transition temperatures were determined using a TA DSC250 instrument at 2nd heating cycle with 10 ^{o}C/min. TGA data was collected using a TA Discovery SDT 650 instrument under air. UV/Vis spectroscopic data was collected using a PerkinElmer Lambda 365 UV/Vis spectroscopy, which the samples were loaded by using PTFE cap sealed quartz cuvettes.

Synthesis of Lewis acidic copolymer Poly-1

The synthetic procedure of compound **1** was synthesized based on published procedures by Piers et al with modifications.[3]

SYN.1 Synthesis of 2,3,5,6-tetrafluoro-3'-methoxy-1,1'-biphenyl

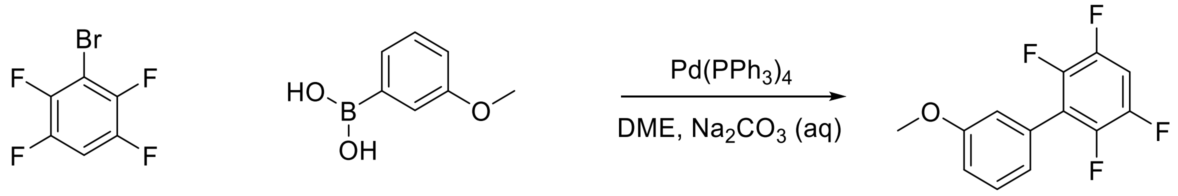

Under nitrogen, 3-methoxyphenylboronic acid (5.0 g, 32.9 mmol), 1M sodium carbonate aqueous solution (82 mL), 1,2-dimethoxyethane (165 mL) and tetrakis(triphenylphopshine)palladium (0) (0.934 g, 0.808 mmol) was added into a three neck round-bottomed flashed which was equipped with a condenser. Then 2,3,5,6-tetrafluoro-4-bromobenzene (6.26 g, 27.3 mmol) was added. The mixture was refluxed for 3-5 days under nitrogen. After cooling down, the product was washed with 2M sodium carbonate solution and the organic phase was extracted with diethyl ether. The solvent was removed under reduced pressure, and the product was passed through a silica flash column using hexane as eluent to remove palladium catalysts. The product was further purified by vacuum sublimation (45-50 ^{o}C at 0.009 mbar).

Yield: 3.54 g, 13.8 mmol, 50.6 %;

^{1}H NMR (400 MHz, $CDCl_3$), δ 7.41 (t, *J* 7.8 Hz, 1H), 7.11 – 6.99 (m, 4H), 3.85 (s, 3H);

^{19}F NMR (376 MHz, $CDCl_3$), δ -139.1 (m, 2F), -143.4 (m, 2F).

SYN.2 Synthesis of dimethyltin bis[4-(2,3,5,6-tetrafluoro-3'-methoxy-1,1'-biphenyl)]

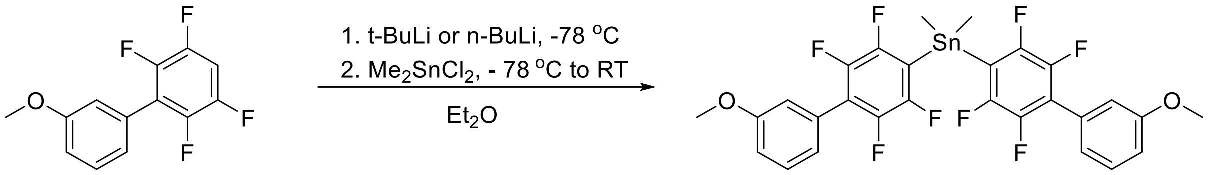

Under nitrogen at -78 °C, 2,3,5,6-tetrafluorophenyl-4-(3-methoxybenzene) (3.52 g, 13.8 mmol) was dissolved in anhydrous diethyl ether (140 mL). Then either *tert*-BuLi (8.11 mL, 1.7 M in heptane) or *n*-BuLi (8.63 mL, 1.6 M in hexane) solution was added dropwise in 1h. Upon addition, a pale gray solution was obtained. Then the mixture was stirred at this temperature for another 2 h. Solid dimethyltin dichloride (1.51 g, 6.87 mmol) was added under nitrogen at -78 °C in several portions. The mixture was then allowed to slowly warmed back to r.t. and stirred overnight. Untreated hexane was added to quench the reaction. Then the solid filtered off and the solvent was concentrated under reduced pressure. Hexane was used to further extract the filtered solid. Any impurities was removed by sublimation under dynamic vacuum at 50-55 °C. The product was obtained as a colorless crystal by recrystallisation from ether/hexane.

Yield: 3.99 g, 6.05 mmol, 88.1 %, in case of using *tert*-BuLi;

^{1}H NMR (400 MHz, $CDCl_3$), δ 7.40 (t, *J* 7.8 Hz, 2H), 7.05 (d, *J* 7.6 Hz, 2H), 6.99 (m, 4H), 3.84 (s, 6H), 0.94 (s, J_{Sn-Me} 64.0 Hz, 6H);

^{19}F NMR (376 MHz, $CDCl_3$), δ -122.7 (m, 4F), -142.1 (m, 4F).

SYN.3 Synthesis of 1

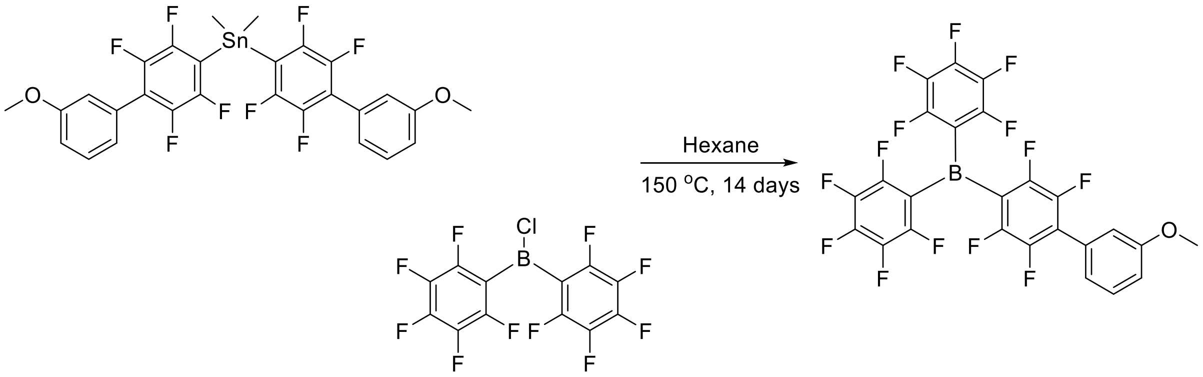

Under nitrogen, dimethyltin bis[4-(2,3,5,6-tetrafluoro-3'-methoxy-1,1'-biphenyl)] (1.34 g, 2.03 mmol) and chlorobis(pentafluorophenyl)borane (1.62 g, 4.27 mmol) was mixed in hexane (50 mL). The mixture was transferred into a high-press glass bomb and heated at 150 °C for 14 days. Upon cooling down, white solid started to precipitate out of the solvent. All contents in mixture were transferred into a sublimation kit and all volatiles was removed under reduced pressure. Dimethyltin dichloride was removed by vacuum sublimation (35 °C, 0.009 mbar). The collected **1** can be further purified by recrystallisation from DCM.

Yield: 1.94 g, 3.23 mmol, 79.7 %;

^{1}H NMR (400 MHz, $CDCl_3$), δ 7.44 (t, *J* 7.8 Hz, 1H), 7.11 (d, *J* 7.8 Hz, 1H), 7.05 (m, 2H), 3.87 (s, 3H);

^{11}B NMR (128 MHz, $CDCl_3$), δ 59.7 (s, br);

^{19}F NMR (376 MHz, $CDCl_3$), δ -127.7 (m, *ortho*-C_6F_5, 4F), -128.9 (m, *ortho*-C_6F_4, 2F), -142.2 (m, *para*-C_6F_5, 2F), -143.2 (m, *meta*-C_6F_4, 2F), -160.1 (m, *meta*-C_6F_5, 2F);

SYN.4 Synthesis of Poly-Si

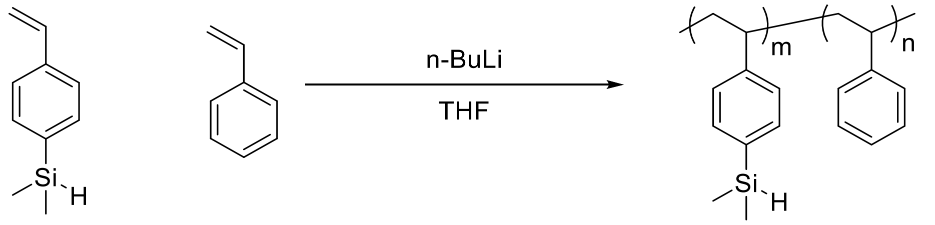

Under inert atmosphere, styrene (1.981 g, 19.0 mmol), 4-VPDMS (0.163 g, 1.00 mmol), and THF (7.0 mL) were introduced into an ampoule. The mixture was cooled down to -78 °C before injection of n-BuLi (0.05 mL, 2.5 M in hexane). The mixture was stirred at this temperature for 1h, then degassed methanol was added to quench the reaction. The mixture was dropwise poured into methanol, and the precipitated polymer product were collected by filtration and dried under vacuum overnight.

Styrene/4-VPDMS/ n-BuLi = 152.2/8/1;

Conversion: Styrene = 4-VPDMS = 100 %;

Theoretical molecular weight = $[mass_{4-VPDMS} + mass_{Styrene}]/n_{n-BuLi}$ + 58.08 = 17300;

GPC Mn = 19300, Đ = 1.13;

^{1}H NMR (400 MHz, $CDCl_3$), δ 7.46-6.22 (m, br, Ar*H*), 4.44 (s, br, Si-*H*), 2.38-1.20 (m, br, backbone-*H*), 0.35 (s, br, Si-*Me*);

^{29}Si DEPT 90 NMR (79 MHz, $CDCl_3$), δ -17.8 (s).

SYN.5 Synthesis of Poly-1

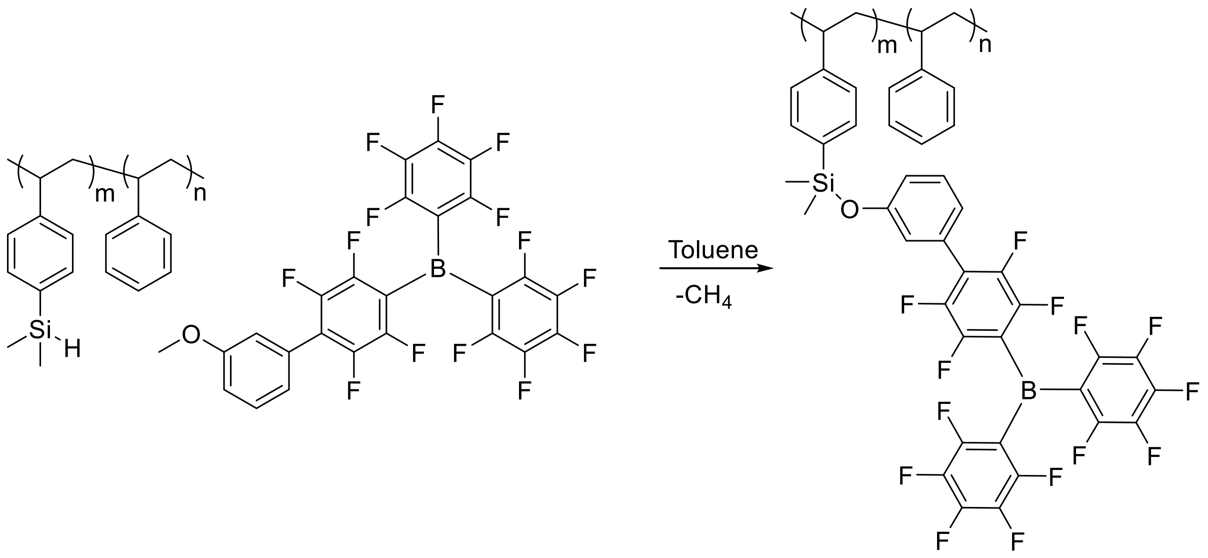

In a glovebox, **Poly-Si** (1.91 g, $M_{n,\ theo}$ = 17208) were dissolved in toluene (20 mL). Then a toluene solution (5 mL) of **1** (0.8 g, 1.33 mmol) was added dropwise, and gas bubbles generated upon addition. The mixture was stirred overnight to ensure complete conversion. The solution was concentrated under reduced pressure, it was then precipitated into cold hexane upon vigorous stirring. The product was collected by filtration, and dried under vacuum.

Yield: 1.41 g, $M_{n,\ theo}$ = 21880, DP = 160, 58.1 %; GPC Mn = 25900, Đ = 1.07;

^{1}H NMR (400 MHz, $CDCl_3$), δ 7.51-6.05 (m, br), 2.30-1.20 (m, br), 0.47 (s, br);

^{19}F NMR (376 MHz, $CDCl_3$), δ -127.8 (s, br, 4F), -128.9 (s, br, 2F), -142.3 (s, br, 2F), -143.2 (s, br, 2F), -160.1 (s, br, 4F);

^{29}Si DEPT90 NMR (79 MHz, $CDCl_3$), δ 9.3 (s).

Synthesis of Lewis Basic Copolymer Poly-2.

SYN.6 Synthesis of 2

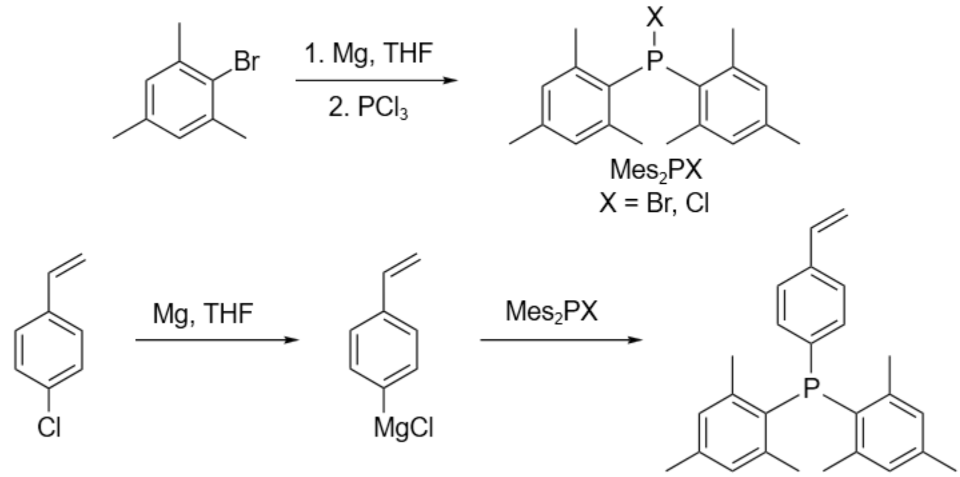

Under nitrogen, 2-bromomesitylene (20 mL) was dropwise added into the mixture of Mg tunings (5.77 g) and THF (200 mL) under reflux in 1 h. After addition the mixture was refluxed for anther 1 h, then stirred at r.t. for additional 3 h. The resultant Grignard solution was dropwise transferred into the THF solution (200 mL) of phosphorus trichloride (5.70 mL) at 0 ^{o}C and stirred at r.t. overnight. All solvent was vacuumed off, then dry hexane (150 mL) was added to extract the product three times. All volatile was removed, and the remaining solvent was transferred into a distillation kit to remove any mono-substituted by-product. The product Mes_2PX was collected as a yellow powder (Yield: 19.4 g, 93.7%, Mes_2PCl:Mes_2PBr = 74:26 by ^{31}P NMR, $MW_{average}$=316.357).

Then under nitrogen, 4-chlorostyrene was added dropwise to a mixture of THF (200 mL) and magnesium tunings (1.84 g) under reflux in 1 h. The mixture was further reflux for 45 min and then stirred at r.t. for 30 min. Then the obtained Grignard solution was dropwise transferred into THF solution of Mes_2PX (19.4 g) under ice bath. The mixture was stirred at r.t. overnight. Saturated brine water was added, and then the mixture was extracted by ether. The combined organic phase was dried over $MgSO_4$, and all volatiles was removed under reduced pressure. The product was further purified by flushing through an neutral Al_2O_3 plug using hexane to remove any oxidized product. The product was recrystallized from methanol, and collected as a white solid.

Yield: 16.6 g, 44.6 mmol, 72.8 %;

^{1}H NMR (400 MHz, C_6D_6), δ 7.41 (t, *J* 8.0 Hz, 2H), 7.08 (d, *J* 7.8 Hz, 2H), 6.75 (s, 4H), 6.49 (dd, *J* 17.6, 10.8, 1H), 5.56 (d, *J* 17.6 Hz, 1H), 5.03 (d, *J* 10.9 Hz, 1H), 2.27 (s, 12H), 2.10 (s, 6H);

^{31}P NMR (162 MHz, C_6D_6), δ -22.3 (s).

SYN.7 Synthesis of Poly-2

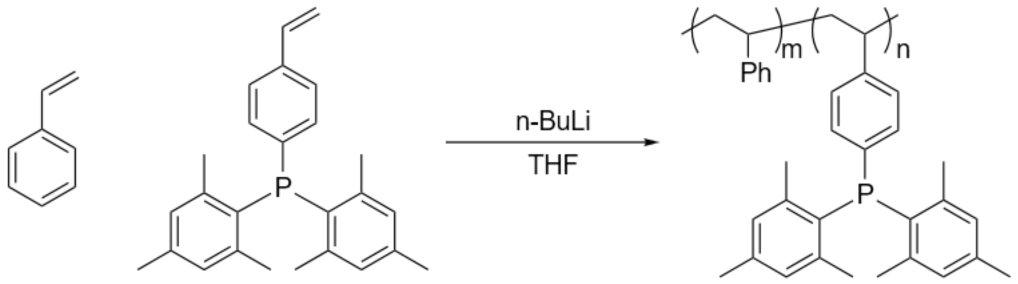

Under inert atmosphere, styrene (1.981 g, 19.0 mmol), **2** (0.372 g, 1.00 mmol), and THF (7.0 mL) were introduced into an ampoule. The mixture was cooled down to -78 ºC before injection of n-BuLi (0.05 mL, 2.5 M in hexane). The mixture was stirred at this temperature for 1h, then degassed methanol was added to quench the reaction. The mixture was dropwise poured into methanol, and the precipitated polymer product were collected by filtration and dried under vacuum overnight.

Styrene/**2**/ n-BuLi = 152.2/8/1;

Conversion: Styrene = **2** = 100 %;

Theoretical molecular weight = $[mass_{\mathbf{2}} + mass_{Styrene}]/n_{n-BuLi}$ + 58.08 = 18900;

GPC Mn = 17800, Đ = 1.08;

^{1}H NMR (400 MHz, $CDCl_3$), δ 7.24-6.87 (m, br, Ar*H*), 6.87-6.18 (m, br, Ar*H*), 2.40-1.68 (m, br, backbone-*H*), 1.48 (m, br, backbone-*H*);

^{31}P NMR (162 MHz, $CDCl_3$), δ -23.0 (s, br).

Synthesis of Lewis Basic Copolymer Poly-3.

SYN.8 Synthesis of 3

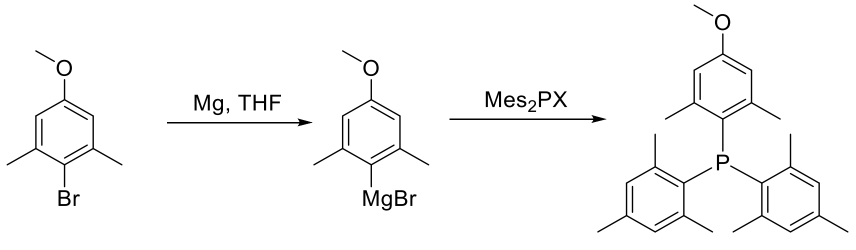

Under inert atmosphere, 4-bromo-3,5-dimethylanisole (6.68 g, 32.5 mmol) was dropwise added into THF (50 mL) suspension of magnesium turnings (0.946 g, 38.9 mmol) in 1h under reflux. After addition, the mixture was refluxed for additional 1 h, the mixture was stirred at r.t. for another 1 h. The synthesized Grignard solution was dropwise transferred into a THF solution (130 mL) of dimesitylphosphorous halide

(Mes_2PCl:Mes_2PBr = 48.9:51.1, $MW_{average}$ = 327.5, 10.6 g, 32.4 mmol) at 0 ^oC. After addition, the mixture was allowed to return to r.t. and stirred overnight. Saturated NaCl aqueous solution was added to quench the reaction. The aqueous phase was extracted by ether (50 mL X 3). The combined organic phase was dried over $MgSO_4$. All volatiles was removed under reduced pressure. The product was later recrystaillsed from methanol, any oxidized product was removed by flush the product through a neutral alumina plug using hexane. The product was obtained as a colorless needle.

Yield: 9.10 g, 22.5 mmol, 69.4 %;

^{1}H NMR (400 MHz, C_6D_6), δ 6.73 (d, *J* 2.9 Hz, 4H), 6.62 (d, *J* 2.4 Hz, 2H), 3.31 (s, 3H), 2.26 (s, br, 18H), 2.10 (s, 6H);

^{31}P NMR (162 MHz, C_6D_6), δ -37.2 (s).

SYN.9 Synthesis of Poly-3

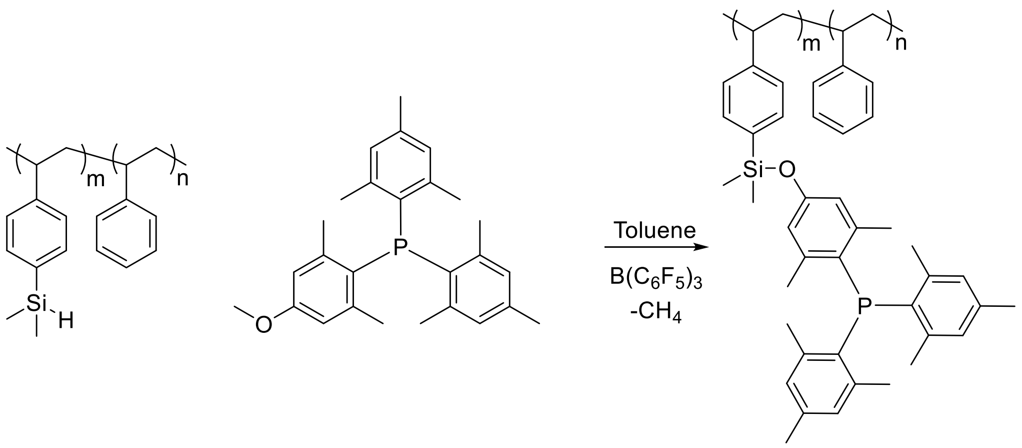

In a glovebox, **Poly-Si** (1.95 g, $M_{n,\ theo}$ = 17327) and tris(pentafluorophenyl)borane (0.05 g, 0.0977 mmol) were dissolved in toluene (20 mL). Then a toluene solution (5 mL) of **2** (0.549 g, 1.36 mmol) was added dropwise, and gas bubbles generated upon addition. The mixture was stirred overnight to ensure complete conversion. The solution was concentrated under reduced pressure, it was then precipitated into methanol upon vigorous stirring. The product was collected by filtration, and then dried under vacuum.

Yield: 1.35 g, $M_{n,\ theo}$ = 20434, DP = 160, 58.7 %; GPC Mn = 22600, Đ = 1.71;

^{1}H NMR (400 MHz, $CDCl_3$), δ 7.39-6.23 (m, br), 2.30-2.14 (m, br), 2.14-1.66 (m, br), 1.66-1.16 (m, br), 0.44 (s, br);

^{31}P NMR (162 MHz, $CDCl_3$), δ -37.2 (s, br);

^{29}Si DPET90 NMR (79 MHz, $CDCl_3$), δ 7.8 (s)

Gutmann-Beckett Lewis Acidity Scale Determination.

To deuterated chloroform dissolved **M1** or **poly-1** solution, stochiometric amount of triethylphosphine oxide was added. The mixture was then characterized by ^{31}P NMR spectroscopy. The Gutmann-Beckett Scale of Lewis acidity was defined as:[4]

$$\text{Acceptor Number (AN)} = (\delta_{Et_3P=O \rightarrow Lewis\ Acid} - \delta_{Et_3P=O\ in\ hexane}) \times 2.21$$

$$= (\delta_{Et_3P=O \rightarrow Lewis\ Acid} - 41.0) \times 2.21$$

SYN.10 NMR data of mixture of 1 and triethylphosphine oxide

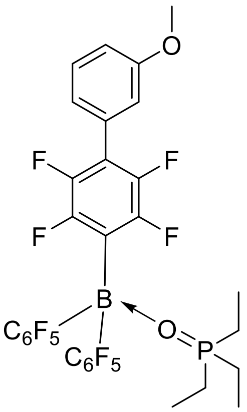

^{1}H NMR (400 MHz, $CDCl_3$), δ 7.38 (dt, *J* 8.3, 2.7 Hz, 1H), 7.11-6.91 (m, 3H), 3.85 (s, 3H), 1.83 (m, 6H), 1.14 (m, 9H);

^{11}B NMR (128 MHz, $CDCl_3$), δ -2.0 (s, br);

^{19}F NMR (376 MHz, $CDCl_3$), δ -134.0 (m, *ortho*-C_6F_5, 4F), -134.7 (m, *ortho*-C_6F_4, 2F), -146.1 (m, *meta*-C_6F_4, 2F), -158.2 (m, *para*-C_6F_5, 2F), -164.2 (m, *meta*-C_6F_5, 2F);

^{31}P NMR (162 MHz, $CDCl_3$), δ 75.6 (s);

AN = (75.6 – 41.0) × 2.21 = 76.5.

SYN.11 NMR data of mixture of Poly-1 and triethylphosphine oxide

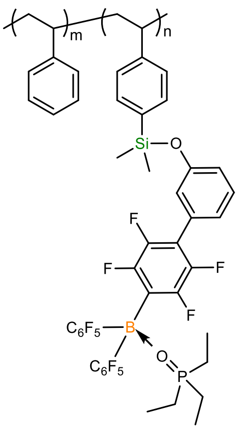

^{1}H NMR (400 MHz, $CDCl_3$), δ 7.39-6.84 (m, br), 6.84-6.25 (m, br), 2.20-0.95 (m, br), 0.84 (s, br);

^{11}B NMR (128 MHz, $CDCl_3$), No detectable peaks;

^{19}F NMR (376 MHz, $CDCl_3$), δ -133.9 (m, *ortho*-C_6F_5, br, 4F), -134.6 (m, *ortho*-C_6F_4, br, 2F), -146.1 (m, *meta*-C_6F_4, br, 2F), -158.2 (m, *para*-C_6F_5, br, 2F), -164.2 (m, *meta*-C_6F_5, br, 2F);

^{31}P NMR (162 MHz, $CDCl_3$), δ 75.6 (s, br);

AN = (75.6 – 41.0) × 2.21 = 76.5.

Small molecule activation by FLP/FRP

SYN.12 Dehydrohalogenation of (1-haloethyl)benzene by 1 and 3.

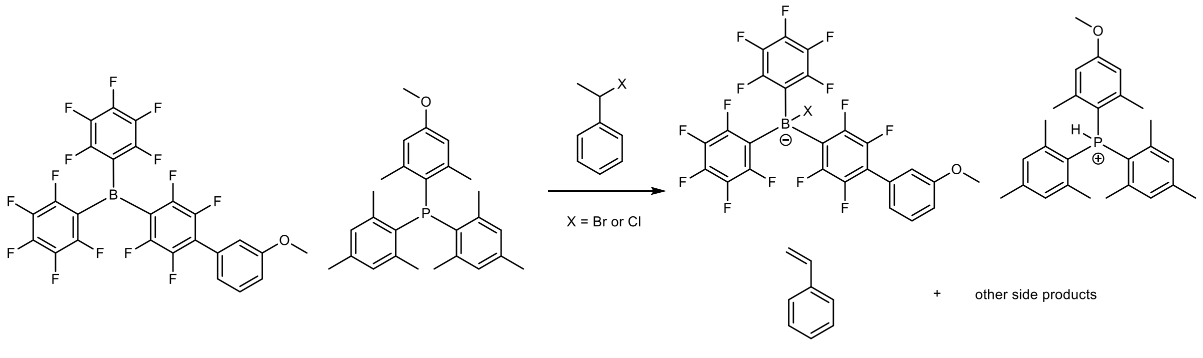

Stochiometric amount of **1** (40 mg, 0.0667 mmol), **3** (27 mg, 0.0667 mmol), and (1-bromoetheyl)benzene (12.3 mg, 0.0667 mmol) or (1-chloroethyl)benzene (9.4 mg, 0.0667 mmol) was added in toluene-d_8 (0.75 mL). The mixture was transferred into a Young's NMR tube.

In the case of (1-chloroethyl)benzene, a reaction can happen overnight at room temperature. Generation of vinyl peaks at was observed in ^{1}H NMR spectrum.

^{1}H NMR (400 MHz, Toluene-d_8), vinyl peaks of styrene was observed: δ 6.54 (dd, *J* 17.4, 11.0 Hz, 1H), 5.57 (d, *J* 17.6, 1.1 Hz, 1H), 5.06 (dd, *J* 10.9, 1.1 Hz, 1H);

^{11}B NMR (128 MHz, $CDCl_3$), δ -6.5 (s, br);

^{19}F NMR (376 MHz, $CDCl_3$), δ -131.7 (ddd, *J* 48.6, 24.2, 8.1 Hz, 4F), -132.5 (dd, *J* 23.2, 12.5 Hz, 2F), -148.1 (dd, *J* 22.8, 12.2 Hz, 2F), -162.0 (t, *J* 20.6 Hz, 2F), -166.6 (tdd, *J* 21.3, 9.2, 4.1 Hz, 4F);

^{31}P NMR (162 MHz, Toluene-d_8) δ -27.6 (d, *J* 482.4 Hz). The spectrum for the crude showed that 70.7 % of phosphorus has been protonated.

In the case of (1-bromoethyl)benzene, no reaction was observed at room temperature. Then the mixture was heated at 80 ^{o}C overnight. The boron and phosphine product was purified by precipitated into hexane and filtered.

^{1}H NMR (400 MHz, Toluene-d_8), vinyl peaks of styrene was observed: δ 6.54 (dd, *J* 17.5, 10.8 Hz, 1H), 5.57 (d, *J* 17.6, 1.0 Hz, 1H), 5.06 (dd, *J* 10.9, 1.0 Hz, 1H);

^{11}B NMR (128 MHz, $CDCl_3$), δ -8.9 (s, br);

^{19}F NMR (376 MHz, $CDCl_3$), δ -130.2 (d, *J* 23.0 Hz, 4F), -131.1 (dd, *J* 23.5, 12.4 Hz, 2F), -148.1 (dd, *J* 23.4, 12.3 Hz, 2F), -161.5 (t, *J* 21.1 Hz, 2F), -166.6 (t, *J* 21.1 Hz, 4F);

^{31}P NMR (162 MHz, Toluene-d_8) δ -27.4 (d, *J* 483.6 Hz). The spectrum for the crude showed that 60.8% of phosphorus has been protonated.

SYN.13 Activation of benzoyl peroxide by 1/3 and Poly-1/Poly-3.

FRP or **Poly(FRP)** was dissolved in solvent. Then 0.5 eqv. of benzoyl peroxide was added, and the solution turned into deep blue colour immediately. The solution was characterized by UV/Vis, EPR and NMR spectroscopy.

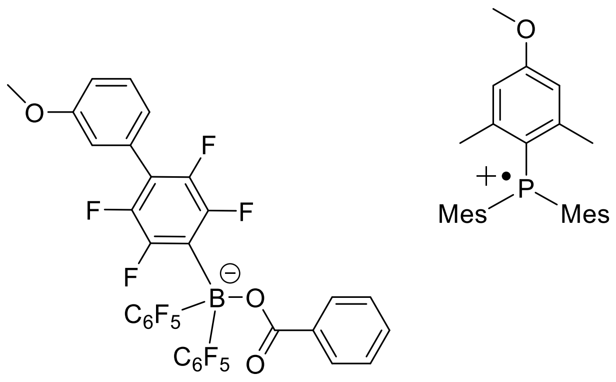

UV/Vis analysis: **1** (5.0 mg, 0.00833 mmol) and **3** (3.4 mg, 0.00840 mmol), benzoyl peroxide (1 mg, 0.00413 mmol), toluene (2.5 mL): λ_{max} = 620 nm;

EPR analysis: **1** (0.800 mg, 0.00133 mmol) and **3** (0.539 mg, 0.00133 mmol), benzoyl peroxide (0.16 mg, 0.000661 mmol), DCM (0.2 mL);

NMR analysis: **1** (20 mg, 0.0333 mmol of boron) and **3** (13.4 mg, 0.0331 mmol), NMR spectra was taken before and after addition of benzoyl peroxide (4.0 mg, 0.0165 mmol), $CDCl_3$ (1.0 mL).

^{11}B NMR (128 MHz, $CDCl_3$), δ -4.3 (s);

^{19}F NMR (376 MHz, $CDCl_3$), δ -132.5 (br), -133.6 (br), -147.3 (br), -160.0 (br), -162.5 (br), -165.6 (br);

^{31}P NMR (162 MHz, $CDCl_3$) Silent.

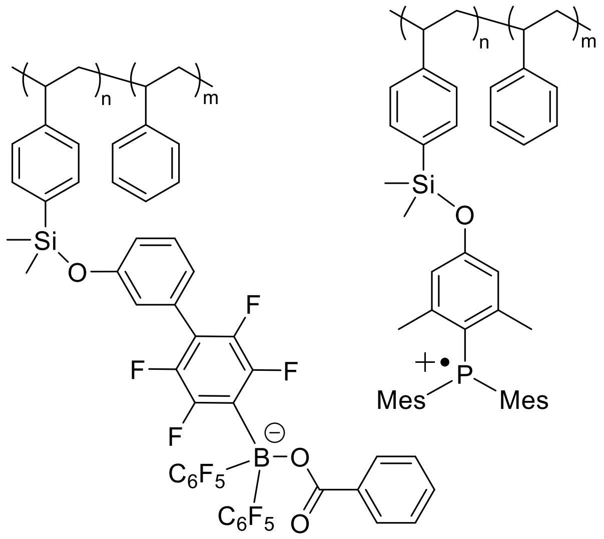

UV/Vis analysis: **Poly-1** (22.4 mg, 0.00819 mmol of boron) and **Poly-3** (20.9 mg, 0.00822 mmol of Phosphorus), benzoyl peroxide (1 mg, 0.00413 mmol), toluene (2.5 mL) :λ_{max} = 596 nm;

EPR analysis: **1** (7.5 mg, 0.00274 mmol of boron) and **3** (6.9 mg, 0.00271 mmol of phosphorus), benzoyl peroxide (0.32 mg, 0.00132 mmol), DCM (0.2 mL);

NMR analysis: **Poly-1** (8.9 mg, 0.00325 mmol of boron) and **Poly-3** (8.3 mg, 0.00326 mmol), benzoyl peroxide (0.4 mg, 0.00165 mmol), DCM-d_2 (1.0 mL).

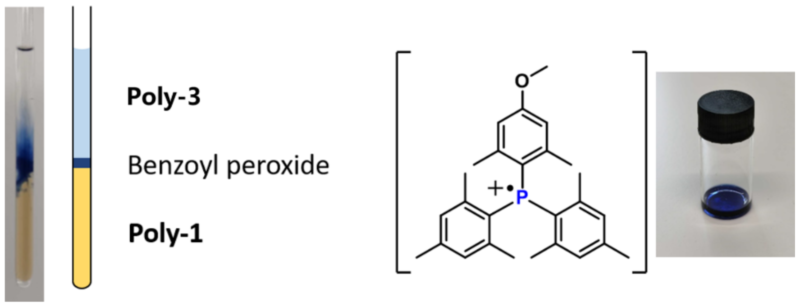

Fig.S1 Images to show the slow formation of polymeric radicals between **Poly-1**/**Poly-3** and benzoyl peroxide (BP). Inside the EPR tube, the reagents were added in sequence as three layers of **Poly-3**, BP and **Poly-1** from top to bottom. The formation of polymeric phosphine radical cations were observed as generation of blue coloured species which slowly diffuse in both directions inside the tube.

SYN.14 Dihydrogen Cleavage by 1/3.

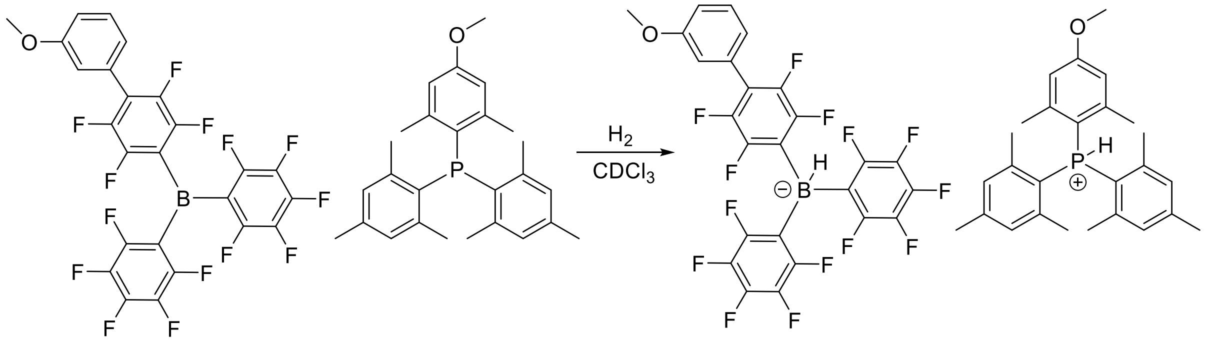

1 (40 mg, 0.0667 mmol) and **3** (27 mg, 0.0667 mmol) were mixed in toluene-*d*8 and the solution was degassed by 3 freeze-pump-thaw cycles. After that dihydrogen gas (1 bar) was introduced and the mixture was stirred at room temperature for 1 h. The solution was poured into could hexane and the product was collected by filtration.

Conversion: determined by ^{31}P NMR of the crude: 93.3%;

^{1}H NMR (400 MHz, $CDCl_3$), δ 8.24 (d, $^{1}J_{H\text{-}P}$ 479.6 Hz, 1H), 7.30 (t, *J* 6.8 Hz, 1H), 7.16-6.94 (m, 6H), 6.89 (ddd, *J* 8.3, 2.6, 1.0 Hz, 1H), 6.80 (s, 1H), 6.70 (s, 1H), 3.82 (s, 3H), 3.80 (s, 3H), 2.34 (s, 6H), 2.24 (s, 9H), 1.96 (s, 9H);

^{11}B {^{1}H} NMR (128 MHz, $CDCl_3$), δ -25.1 (s);

^{11}B NMR (128 MHz, $CDCl_3$), δ -25.1 (d, *J* 90.7 Hz);

^{31}P {^{1}H} NMR (162 MHz, $CDCl_3$) δ -27.2 (s);

^{31}P NMR (162 MHz, $CDCl_3$) δ -27.2 (d, $^{1}J_{P\text{-}H}$ 479.1 Hz).

SYN.15 Dihydrogen Cleavage by Poly-1/Poly-3.

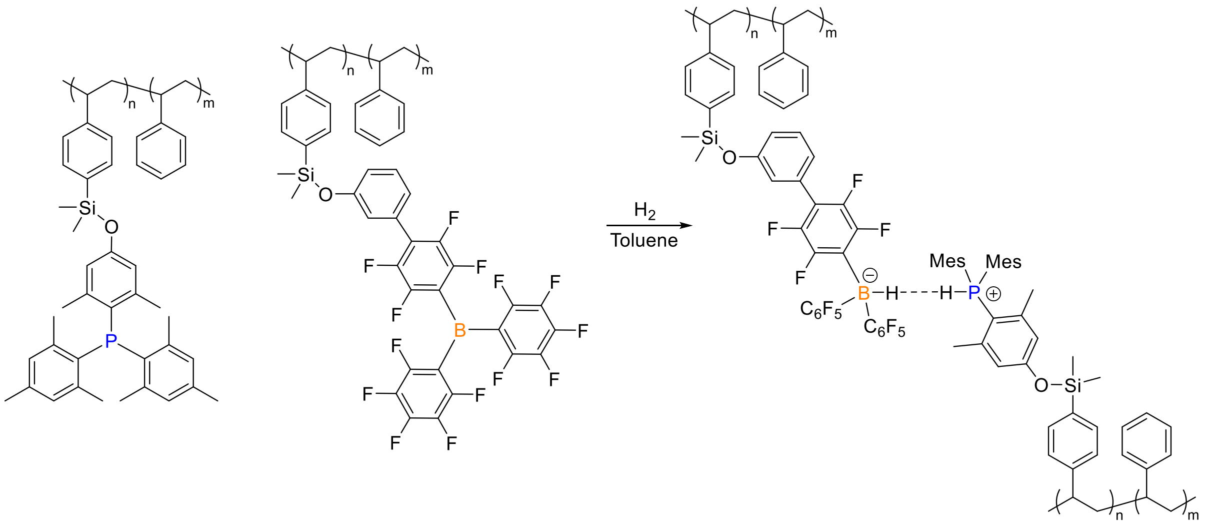

Poly-1 (50 mg, $M_{n,theo}$ = 33306, 0.0270 mmol of boron moities) and **Poly-3** (46 mg, $M_{n,theo}$ = 29805, 0.0272 mmol of phosphorus moities) were mixed in toluene-*d*8 and the solution was degassed by 3 freeze-pump-thaw cycles. After that dihydrogen gas (1 bar) was introduced and the mixture was stirred at room temperature overnight for complete reaction. The mixture was grinded into small particles and then transferred into a Young's NMR tube and tested by NMR spectroscopy.

^{1}H NMR (400 MHz, Toluene-*d*8), δ 7.35-6.02 (m, br), 2.51-1.85 (m, br), 1.85-0.99 (m, br), 0.49 (s, br);

^{31}P {^{1}H} NMR (162 MHz, Toluene-*d*8) δ -28.0 (s, br, [**Poly-1**-H]$^{-}$/[**Poly-3**-H]$^{+}$, 83.1%), -37.1 (s, br, unreacted **Poly-3**, 16.9%);

^{31}P NMR (162 MHz, Toluene-d_8) δ -28.1 (d, ^{1}J $_{P\text{-}H}$ 485.2 Hz, br, [**Poly-1**-H]$^-$/[**Poly-3**-H]$^+$, 83.1%), -37.1 (s, br, unreacted **Poly-3**, 16.9%).

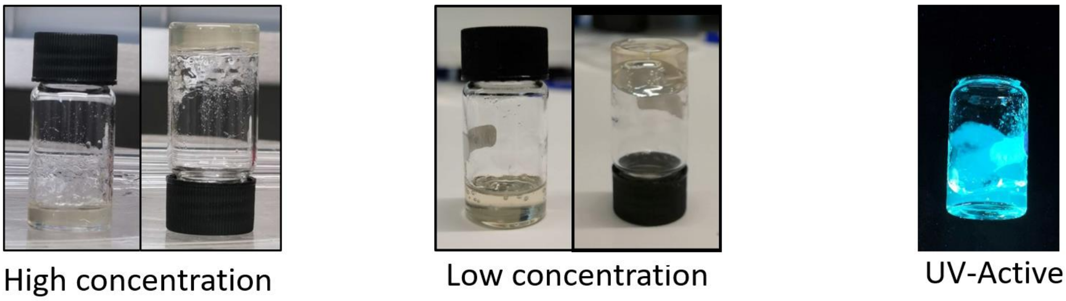

Fig.S2 Poly(FRP) solution without addition of any activable molecules. At high concentration of boron/phosphorus moieties (67.6 mM, 10 mol% loading on polymer chains, 18 B/P per chain, **Poly-1** $M_{n,theo}$=33300, **Poly-3** $M_{n,theo}$=29800), the polymer solution behave like a 'gel', while at low concentration (33.8 mM of B/P moieties) it was more fluidic. Monitoring the sample with long-range UV lamp indicating non-coordinating boron, hence the interactions between polymer chains come from the weak interactions between Lewis acids and Lewis bases.

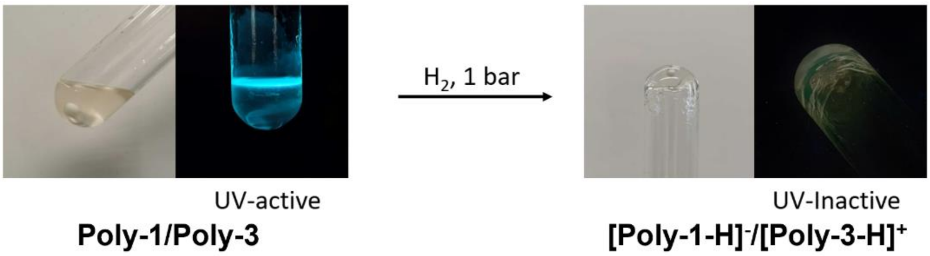

Fig.S3 Transformation of **Poly(FRP)** solution into **Poly(FRP) gel** by dihydrogen activation. Before introducing of hydrogen gas, the boron moieties remain UV-active (long-range UV). After activation, the boron moieties become no longer UV-active due to the coordination by hydride anion.

SYN.16 Activation of DEAD and Propylene Oxide by Poly-1/Poly-2 or Poly-1/Poly-3

Poly-1/Poly-2 or **Poly-1/Poly-3** was dissolved in toluene (0.5 mL) at concentration of 0.0312 mM (by B/P functionalities). Then 2.5 eqv. of diethyl azodicarboxylate (DEAD) or propylene oxide was added, and the mixture was allow to stand overnight at r.t.

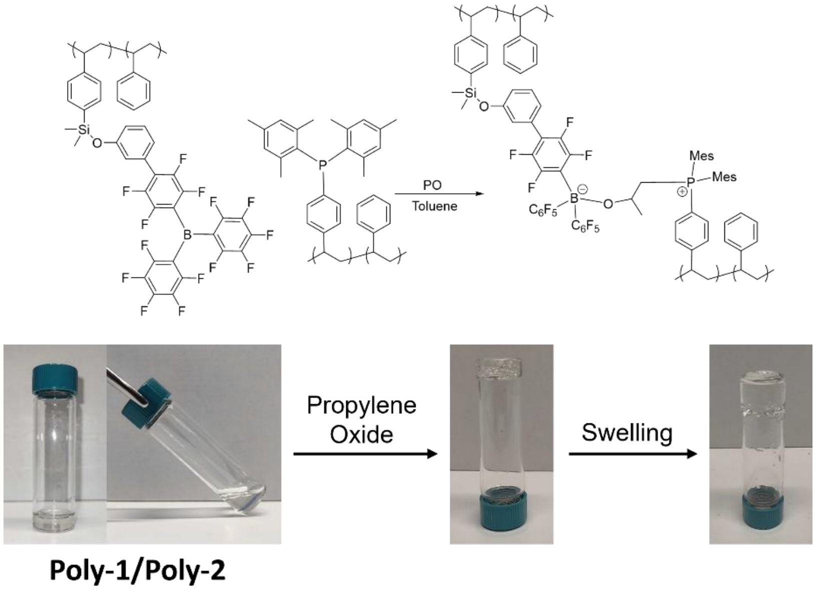

Fig.S4 Gelation between **Poly-1** and **Poly-2** by propylene oxide in toluene.

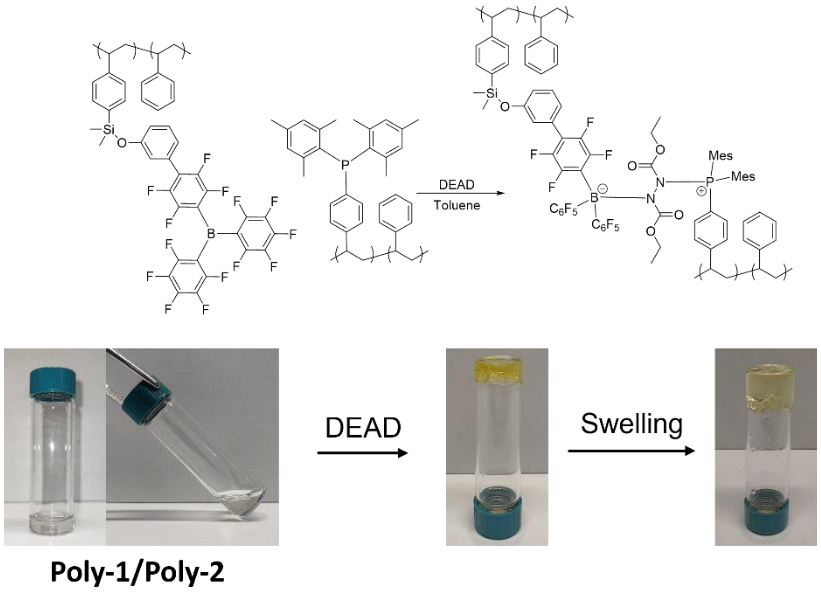

Fig.S5 Gelation between **Poly-1** and **Poly-2** by DEAD in toluene.

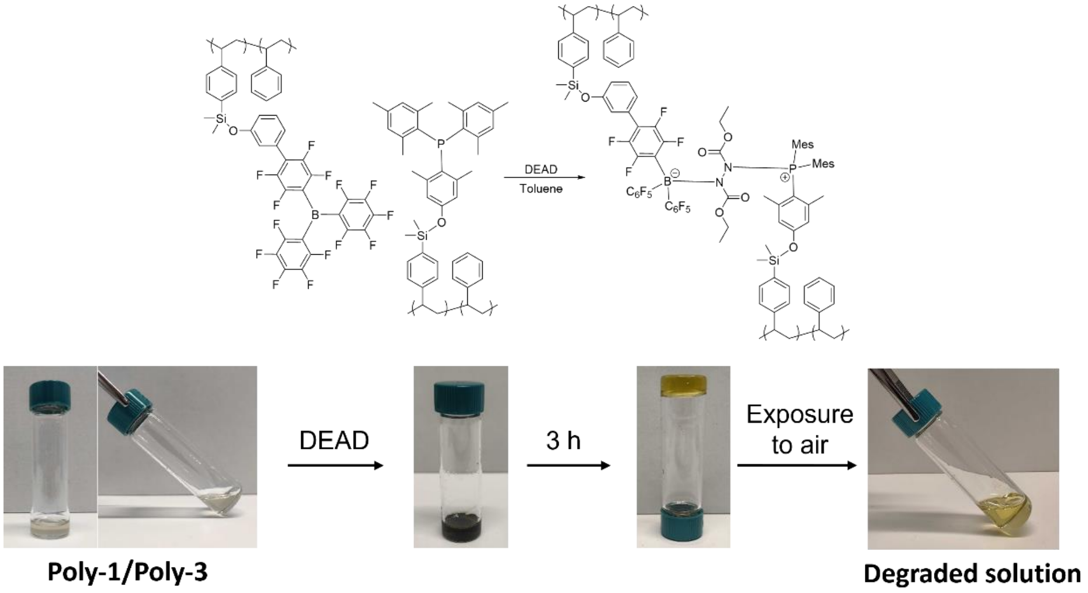

Fig.S6 Gelation between **Poly-1** and **Poly-3** by DEAD in toluene.

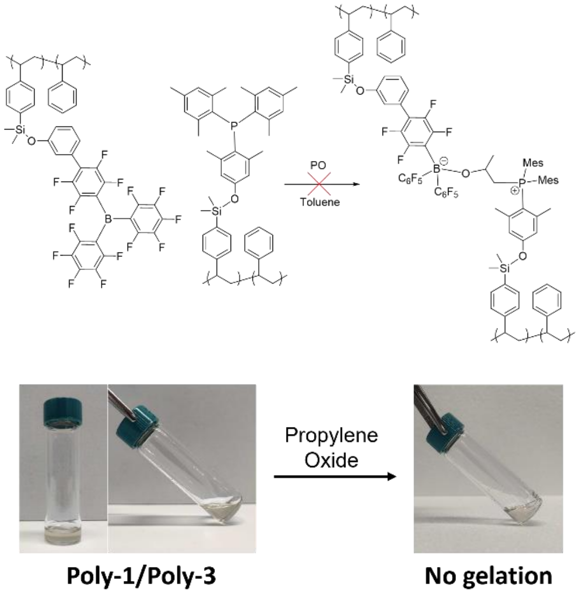

Fig.S7 Gelation Attempts between **Poly-1** and **Poly-3** by propylene oxide in toluene.

SYN.17 Diaryl-ester Activation with Styrene by FRP or Poly(FRP)

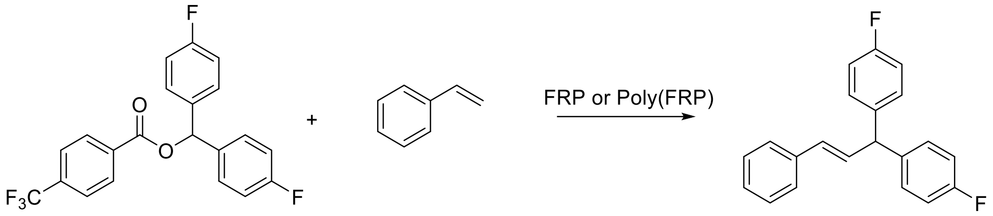

Styrene (53 mg, 5 eqv.) were mixed with **1/3** or **Poly-1/Poly-3** (1 eqv. of FRP moieties) in anhydrous solvent in glovebox. Then Bis(4-fluorophenyl)methyl 4-(trifluoromethyl)benzoate (40 mg, 1 eqv.) was added. The mixture was heated for desired temperature and time and conversion was determined by addition of an internal standard 1,3,5-triispropyl-2,4,6-trioxane before NMR testing.

Table.S1 Reaction conditions for diaryl-ester activation coupling reaction with styrene by FRP or Poly(FRP)

Entry	Catalyst	Solvent	Temperature / ^oC	Time / h	Conversion / %
1	**1/3**	THF	70	7	41.0
2	**Poly-1/Poly-3**	THF	70	7	10.3
3	**Poly-1/Poly-3**	THF	80	7	9.0
4[a]	**Poly-1/Poly-3**	THF	70	7	5.7
5[b]	**Poly-1/Poly-3**	Styrene	70	7	5.8

[a] Stirred overnight at RT before heating to check if the conversion is limited due to slow initiation.

[b] Bulk styrene was used as solvent to check if the conversion is limited by the slow diffusion of reagents in the viscous polymer solution.

SYN.18 Perfluoroalkylation by FRP or Poly(FRP)

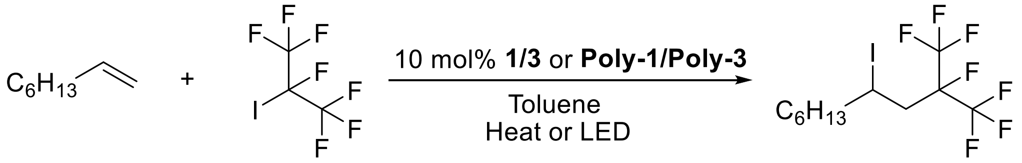

1-Octene, 2-iodoheptafluoropropane, 10 mol% of either FRP **1/3** or Poly(FRP) **Poly-1/Poly-3** was mixed in deuterated toluene. The mixture was either heated or radiated by LED source with desired time. After reaction, the mixture was either transferred into a NMR tube for analysis directly, or purified by passing through a flash silica

column followed by removal of volatiles under reduced pressure. The product was obtained as a colourless/pale yellow oil.

^{1}H NMR (400 MHz, $CDCl_3$), δ 4.33 (tt, *J* 8.9, 4.6 Hz, 1H), 2.87 (dpd, *J* 33.1, 16.2, 6.6 Hz, 2H), 1.76 (dtd, *J* 20.9, 9.5, 4.9 Hz, 2H), 1.59-1.17 (m, 8H), 0.99-0.78 (m, 3H);

^{13}C NMR (101 MHz, $CDCl_3$), δ 41.0, 39.8, 31.7, 29.8, 28.3, 23.1, 22.7, 14.2;

^{19}F NMR (376 MHz, $CDCl_3$), δ -76.0 (p, J 9.0 Hz, 3F), -77.4 (qd, *J* 9.3, 6.6 Hz, 3F), -185.5 (ddq, *J* 33.8, 14.1, 7.1 Hz, 1F).

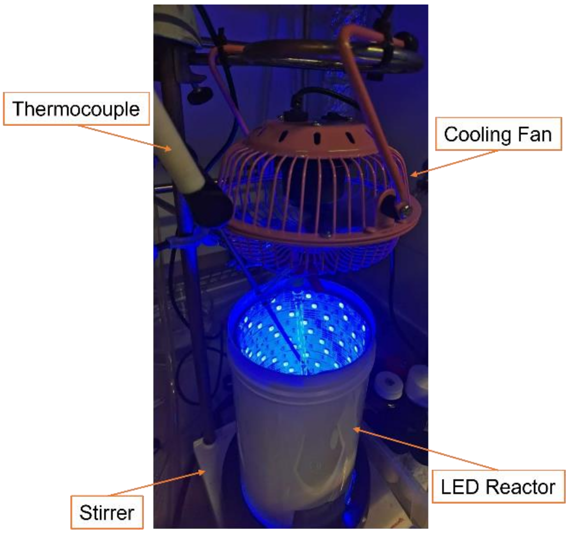

The reaction was performed using a LED device shown above. The inner side of the reactor was composed of LED arrays. On top of the reactor there is an electric fan on top to maintain the temperature of the inner space of the reactor. The temperature was monitored using the thermocouple. The reaction mixture can be placed either in a colorless transparent Pyrex glass-made Young's NMR tube, or a well-sealed sample vial.

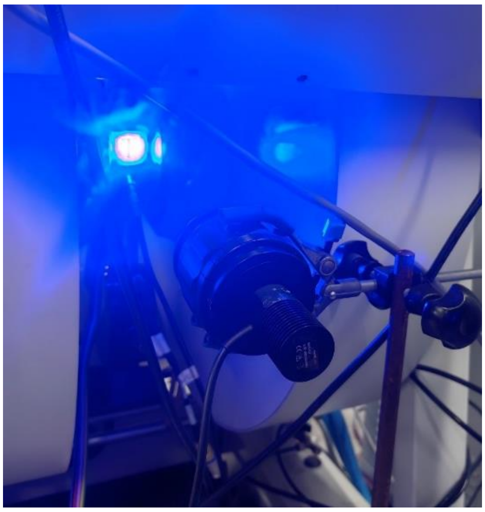

The light irradiated EPR analysis was performed as shown above. The light was irradiated directly to the sample cell of the EPR spectrometer. The light source used was Thorlabs M455L3 (455 nm, Bandwidth (FWHM): 18 nm).

Table.S2 Perfluoroalkylation of alkenes catalysed by FRP or poly(FRP) under visible light irradiation.

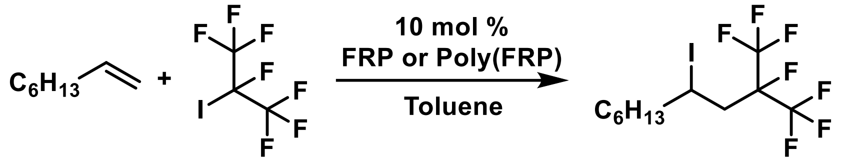

Entry	Catalyst	Light Source	Reaction Time/ h	Conversion / %
1	**1/3**	Heat 80 °C	10	42.5
2	**1/3**	Blue LED	10	79.2
3	**1/3**	Blue LED	24	> 99
4	**1/3**	Red LED	10	7.6
5	**1/3**	Green LED	10	36.5
6[a]	**1/3**	None	10	< 1.0
7[b]	**1/3**	Blue LED	10	0
8[c]	**1/3**	Blue LED	10	0
9	**1**	Blue LED	10	32.3
10	**3**	Blue LED	10	62.2
11	None	Blue LED	10	3.5
12	**Poly-1**/**Poly-3**	Heat 80 °C	10	7.1
13	**Poly-1**/**Poly-3**	Blue LED	10	58.9
14	**Poly-1**/**Poly-3**	Blue LED	24	95.3
15	**Poly-1**/**Poly-3**	Red LED	10	2.1
16	**Poly-1**/**Poly-3**	Green LED	10	22.6
17	**Poly-1**	Blue LED	10	22.8
18	**Poly-3**	Blue LED	10	37.4

Reaction time: 10 h, ambient temperature. [a] reaction time: 40 h. [b] TEMPO was added. [c] hydroquinone was added.

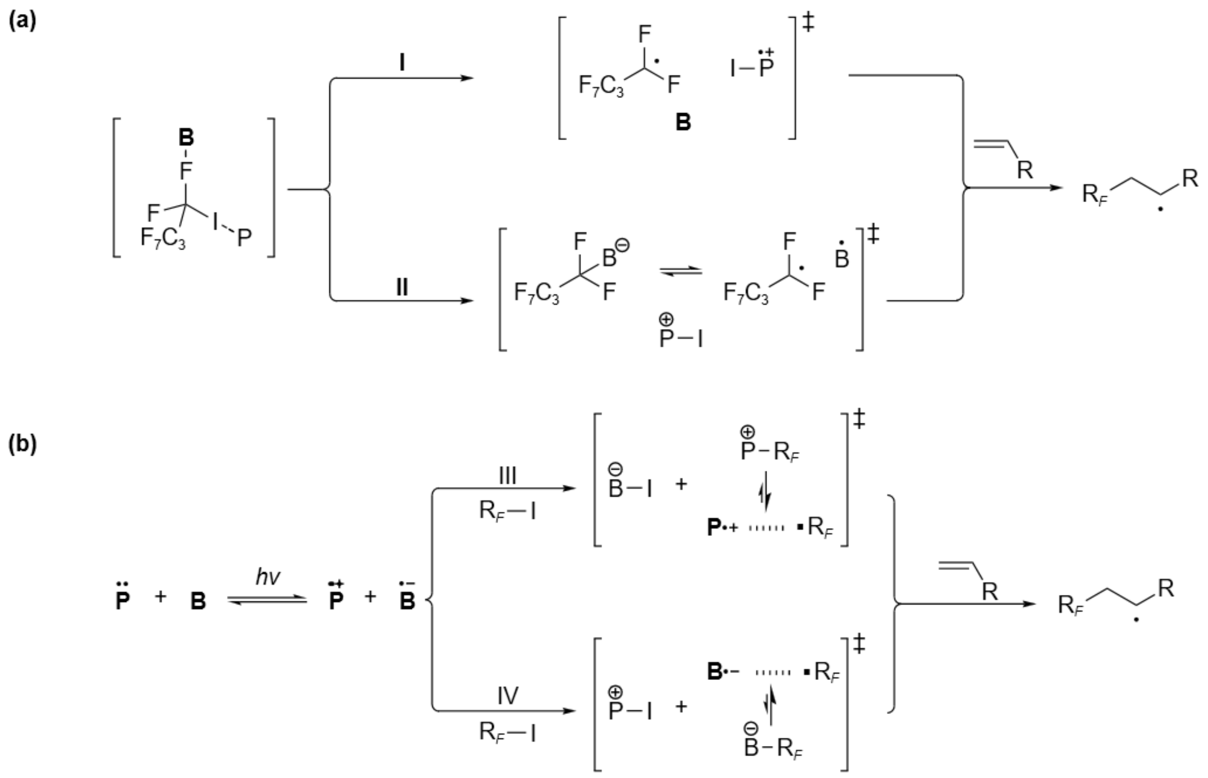

Scheme.S1: (a) Czekelius-proposed mechanism using a stronger LB $^tBuP_3$ ([5], Copyright 2016, reproduced with permission from John Wiley & Sons) and (b) Proposed mechanism for FRP-mediated alkene-perfluoroalkylation by this work. As no interactions/reactions was observed between **1**, **3**, 1-octene, and 2-iodoheptafluoropropane without heating or light irritation, hence the reaction was proposed to start only upon formation of radical pairs. Evidenced by EPR spectra, both boron and phosphine radicals were observed during the reaction, which the boron radicals were consumed at a faster rate, hence it was deduced that both pathways III and IV can occur (i.e. boron species participate into reaction in a similar manner to phopshine, rather than simple coordination), which pathway III is faster.

SYN.19 Poly-1/Poly-3 Catalyzed Hydrogenation of N-benzylidene-*tert*-butylamine

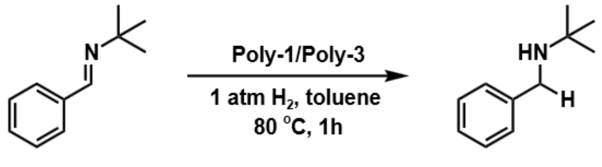

Poly-1 (10 mol% of boron loading, 78 mg, 0.024 mmol of boron), **Poly-3** (10 mol% of phosphine loading, 74 mg, 0.025 mmol of phosphorus), N-benzylidene-tert-butylamine (0.0816 g, 0.5 mmol), and toluene (3 mL) was mixed in an ampoule inside a glovebox. Then solution was degassed three times by freeze-pump-thaw. Then 1 bar of dihydrogen gas was introduced into the ampule and the mixture was stirred at 80 ^oC for 1h. An aliquot of this reaction mixture was analyzed by NMR spectroscopy.

NMR Spectroscopic Data

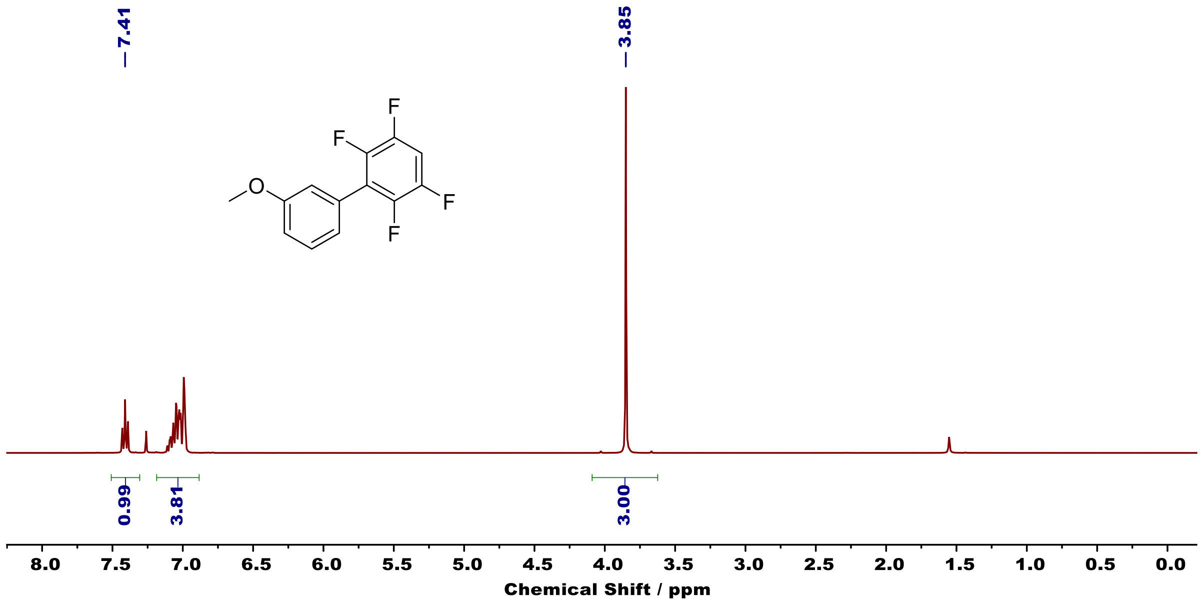

Fig.S8 ^{1}H NMR (400 MHz, $CDCl_3$) spectrum of 2,3,5,6-tetrafluorophenyl-4-(3-methoxybenzene).

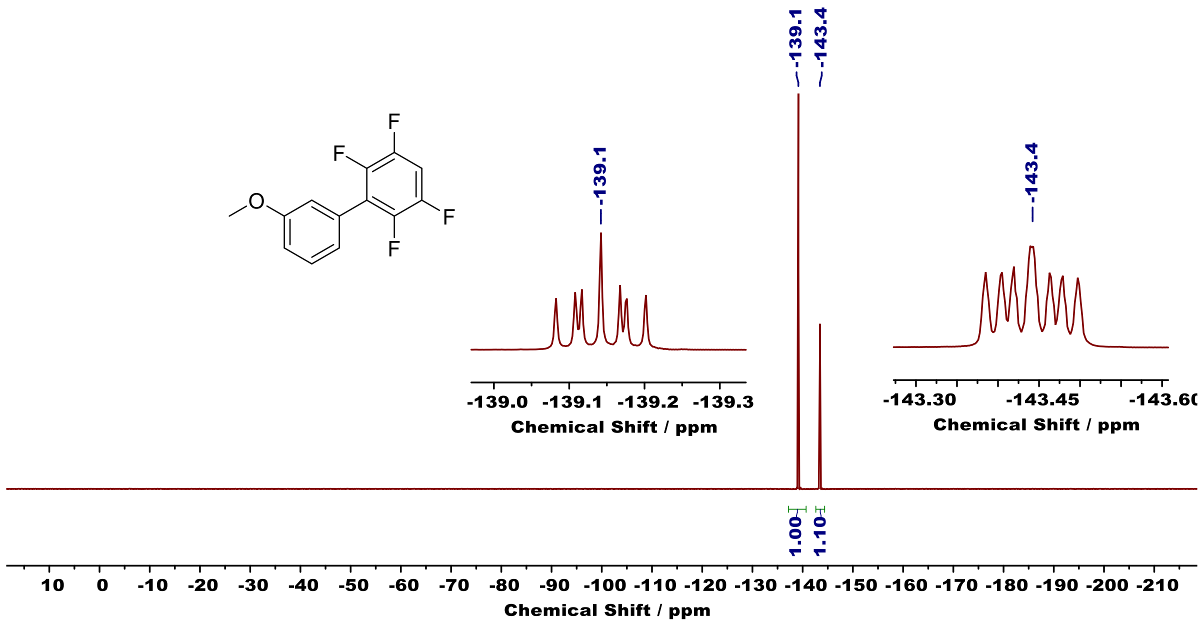

Fig.S9 ^{19}F NMR (376 MHz, $CDCl_3$) spectrum of 2,3,5,6-tetrafluorophenyl-4-(3-methoxybenzene).

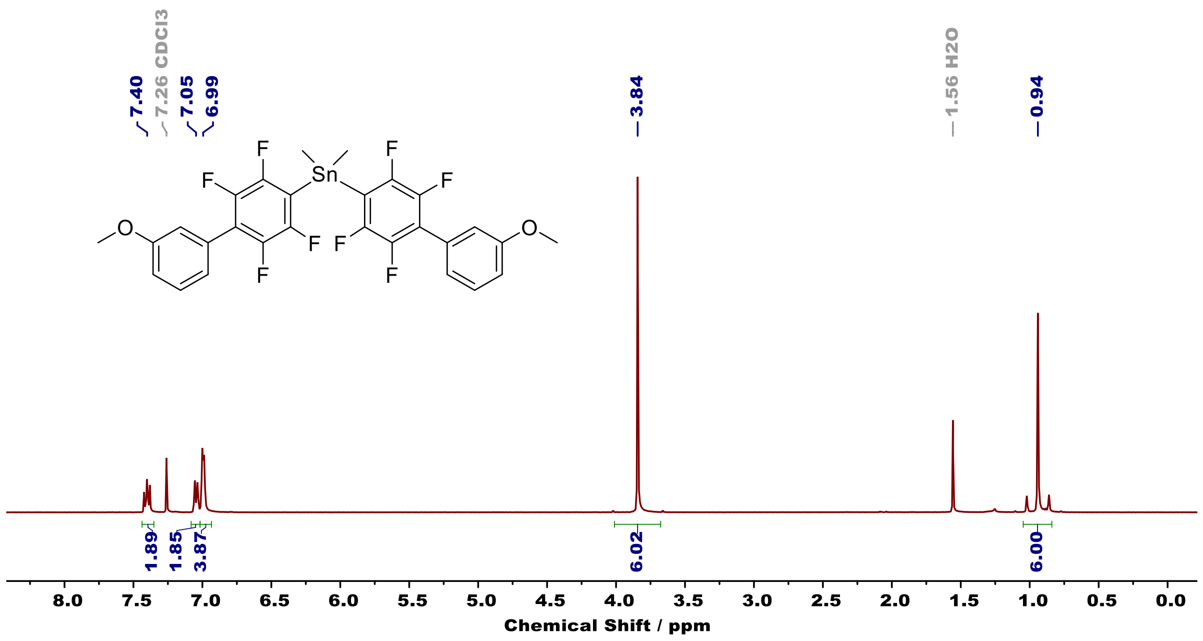

Fig.S10 ^{1}H NMR (400 MHz, $CDCl_3$) spectrum of dimethyltin bis(2,3,5,6-tetrafluorophenyl-4-(3-methoxybeznene)).

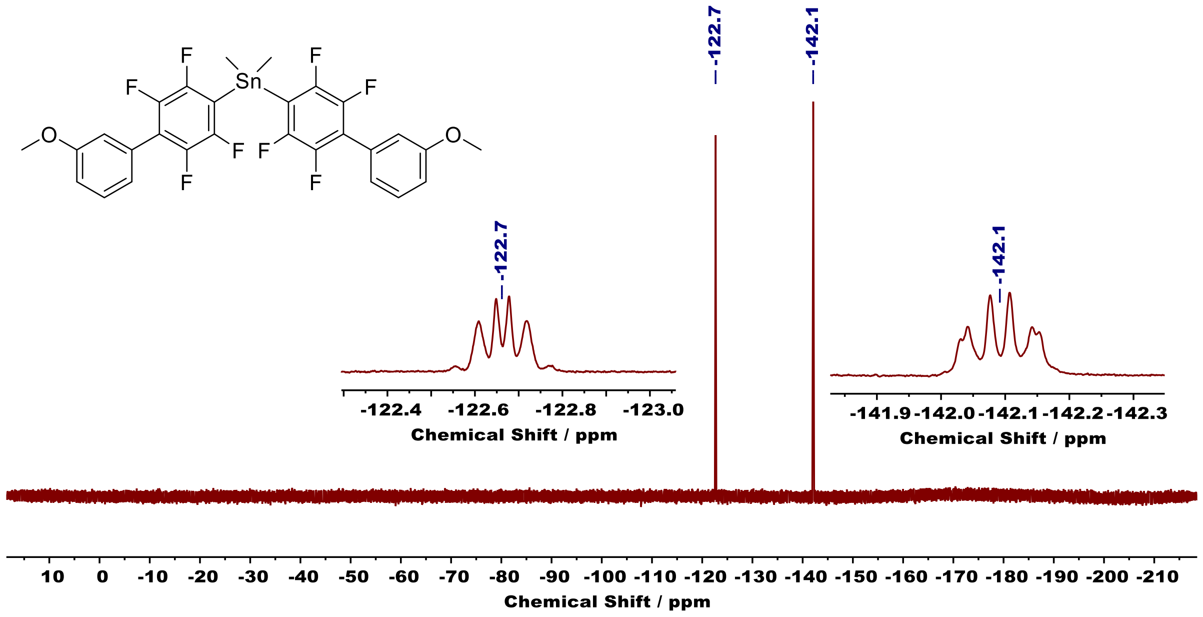

Fig.S11 ^{19}F NMR (376 MHz, $CDCl_3$) spectrum of dimethyltin bis(2,3,5,6-tetrafluorophenyl-4-(3-methoxybeznene)).

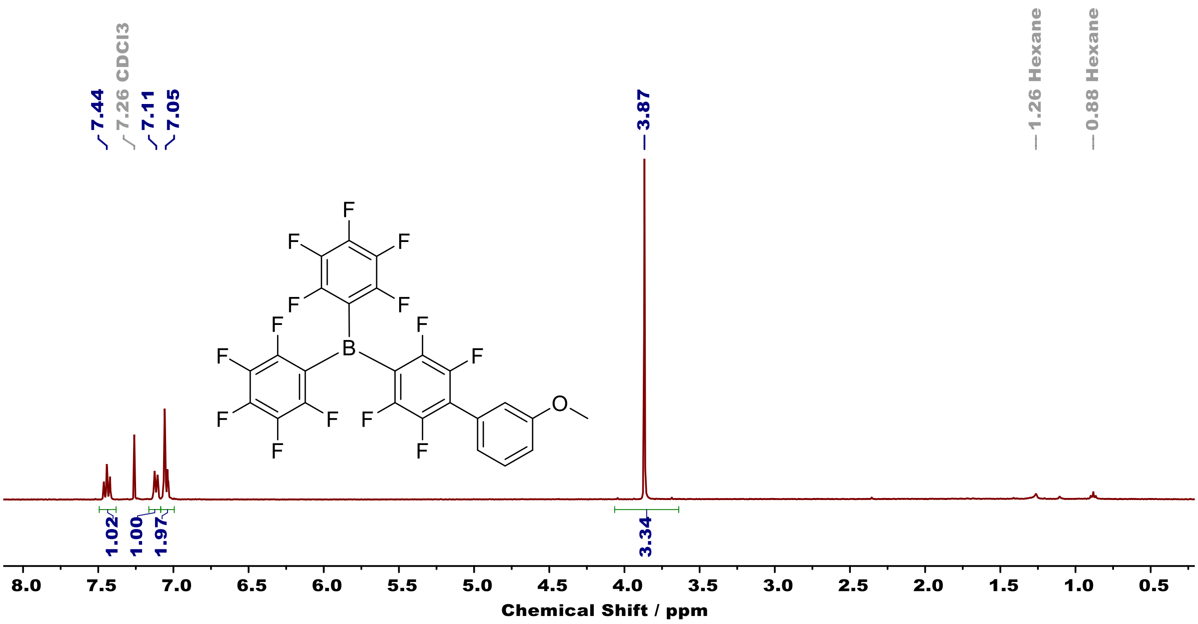

Fig.S12 ^{1}H NMR (400 MHz, $CDCl_3$) spectrum of **1**.

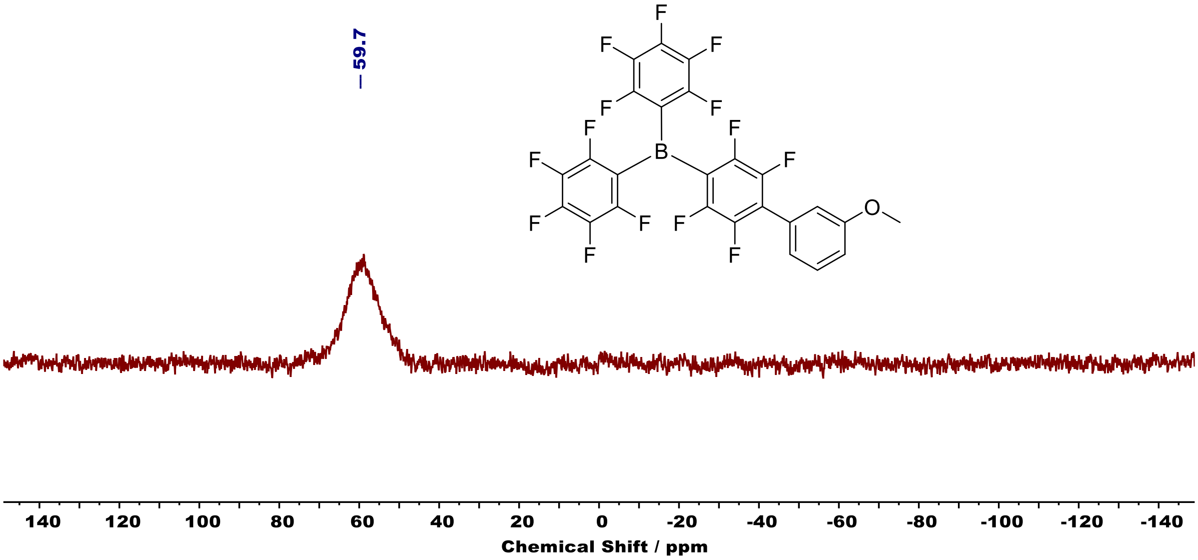

Fig.S13 ^{11}B NMR (128 MHz, $CDCl_3$) spectrum of **1**.

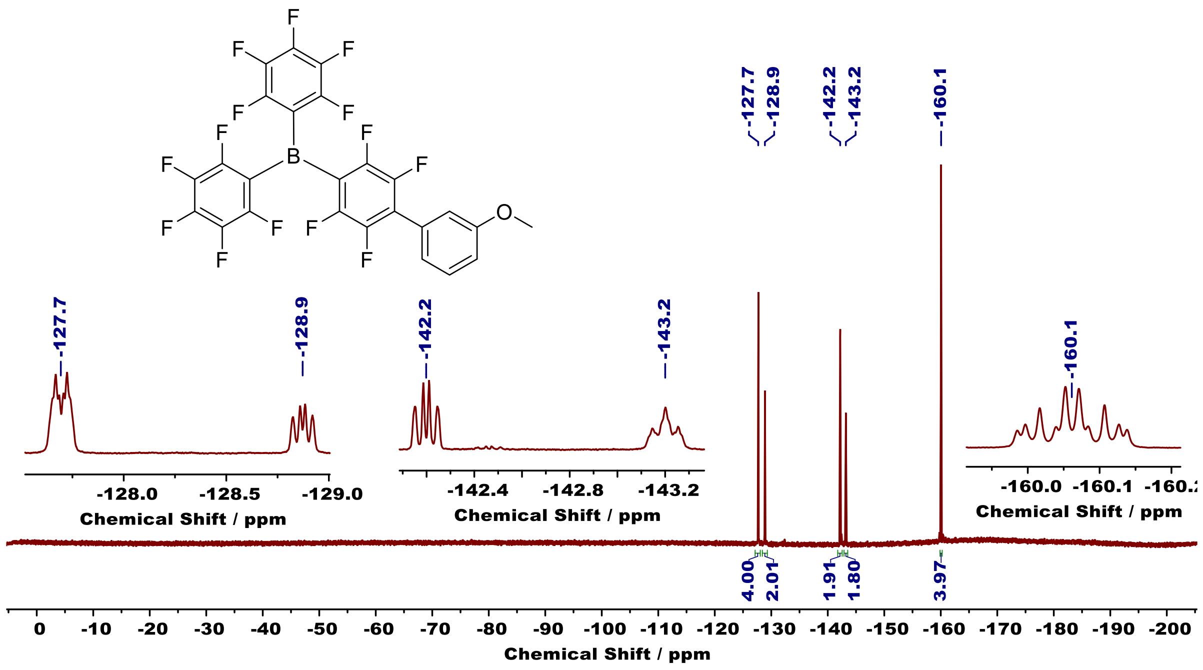

Fig.S14 ^{19}F NMR (376 MHz, $CDCl_3$) spectrum of **1.**

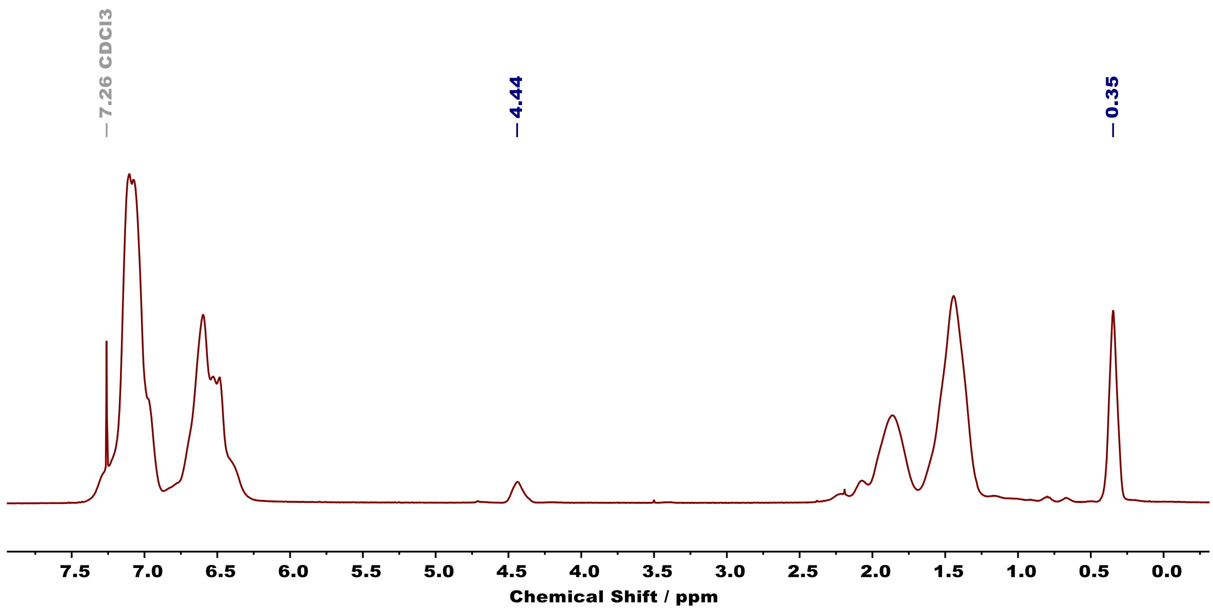

Fig.S15 ^{1}H NMR (400 MHz, $CDCl_3$) spectra of **Poly-Si.**

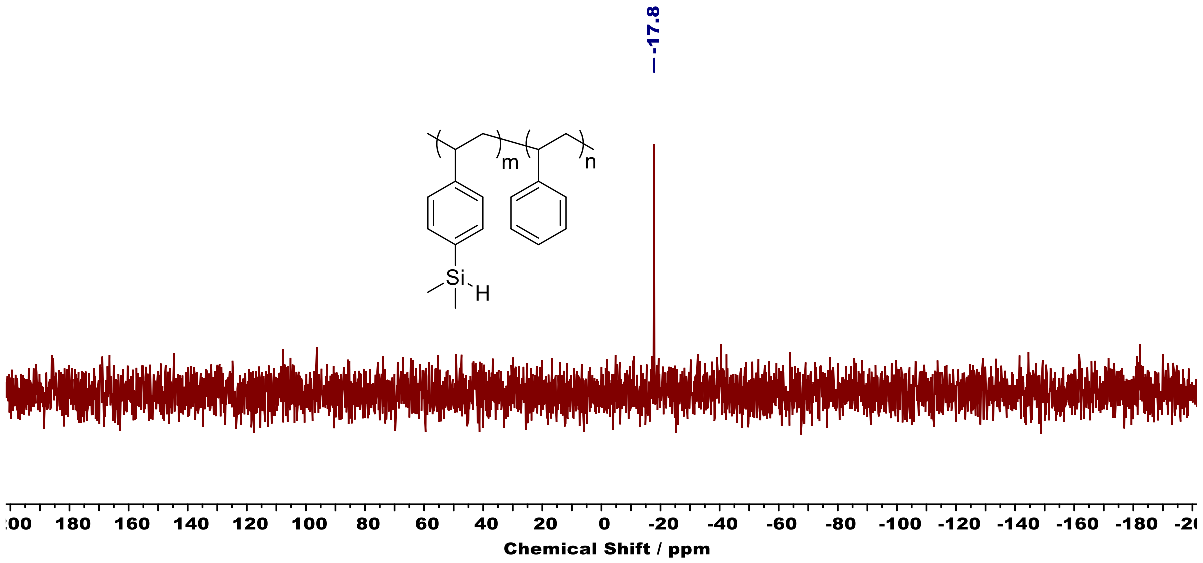

Fig.S16 ^{29}Si DEPT 90 NMR (79 MHz, $CDCl_3$) spectra of **Poly-Si.**

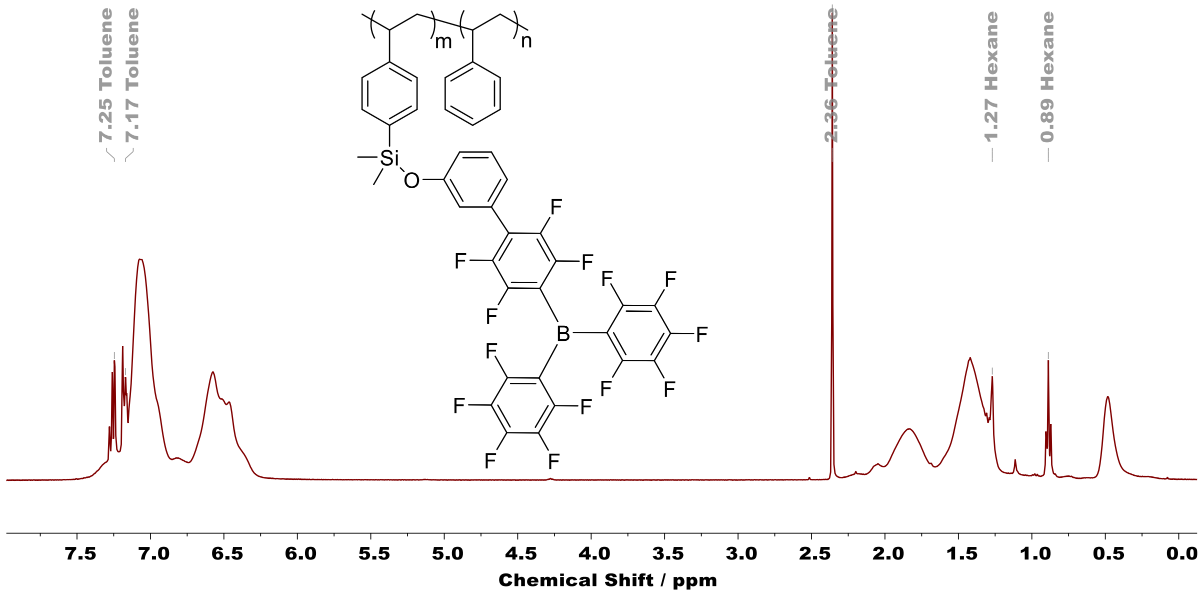

Fig.S17 ^{1}H NMR (400 MHz, $CDCl_3$) spectra of **Poly-1.**

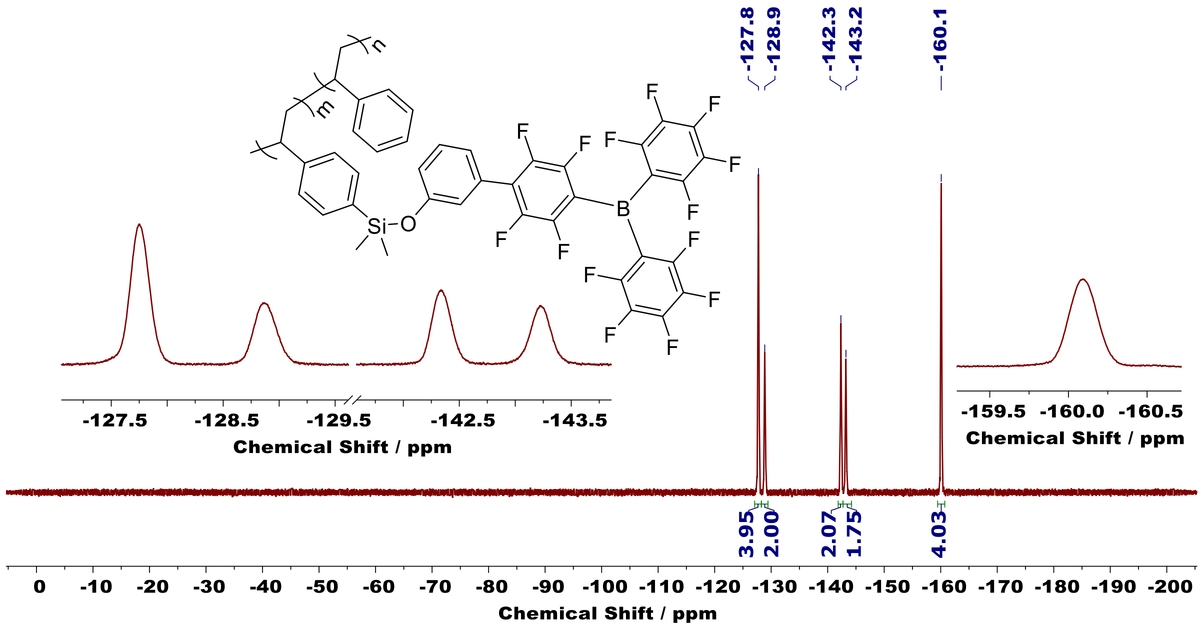

Fig.S18 ^{19}F NMR (376 MHz, $CDCl_3$) spectra of **Poly-1.**

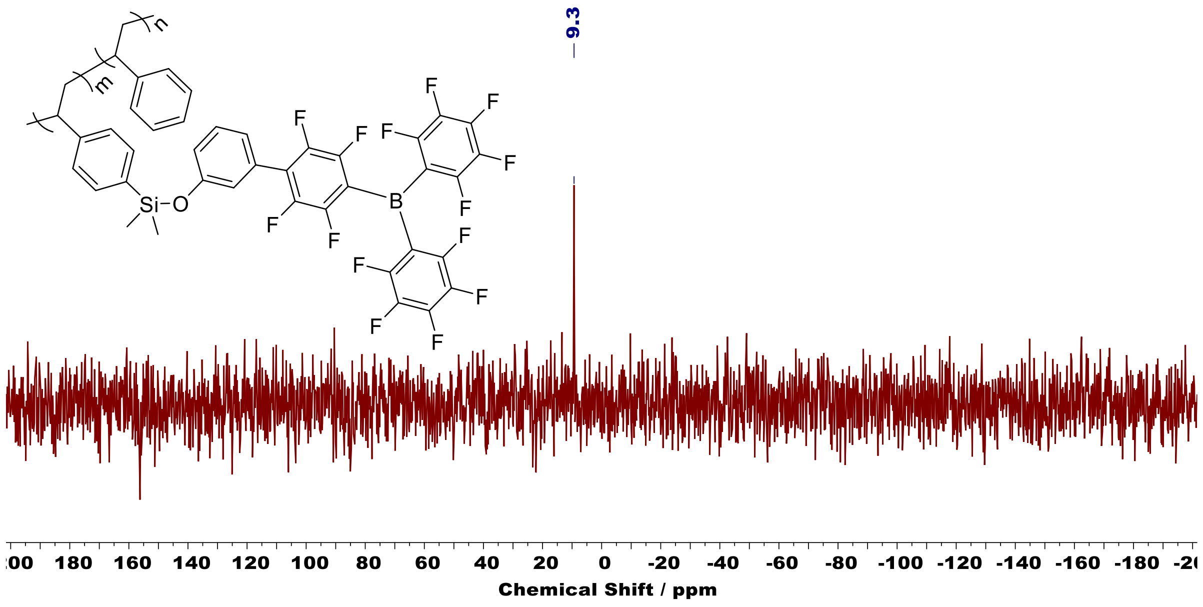

Fig.S19 ^{29}Si DEPT90 NMR (79 MHz, $CDCl_3$) spectra of **Poly-1.**

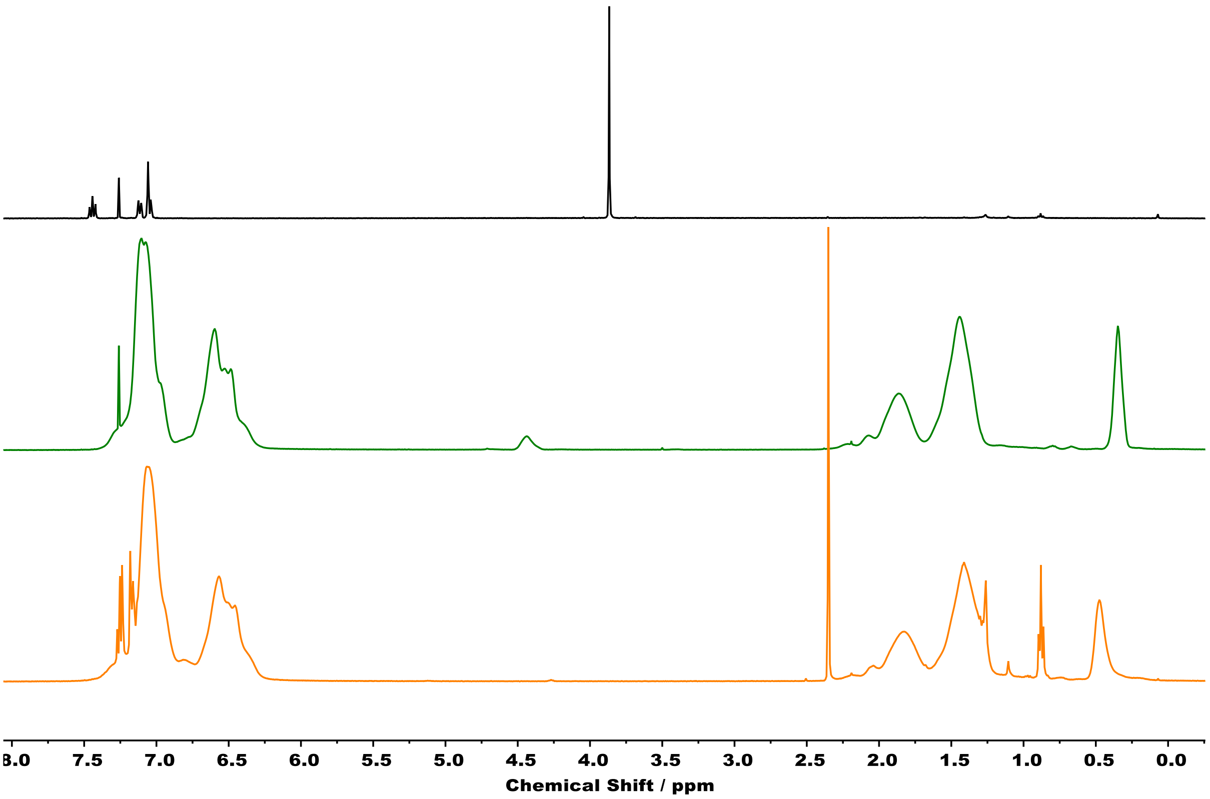

Fig.S20 Stacked ^{1}H NMR (500 MHz, $CDCl_3$) spectra of post-polymerisation modification reaction between **Poly-Si** and **1**. Top: **1**; middle: **Poly-Si**; bottom: **Poly-1**.

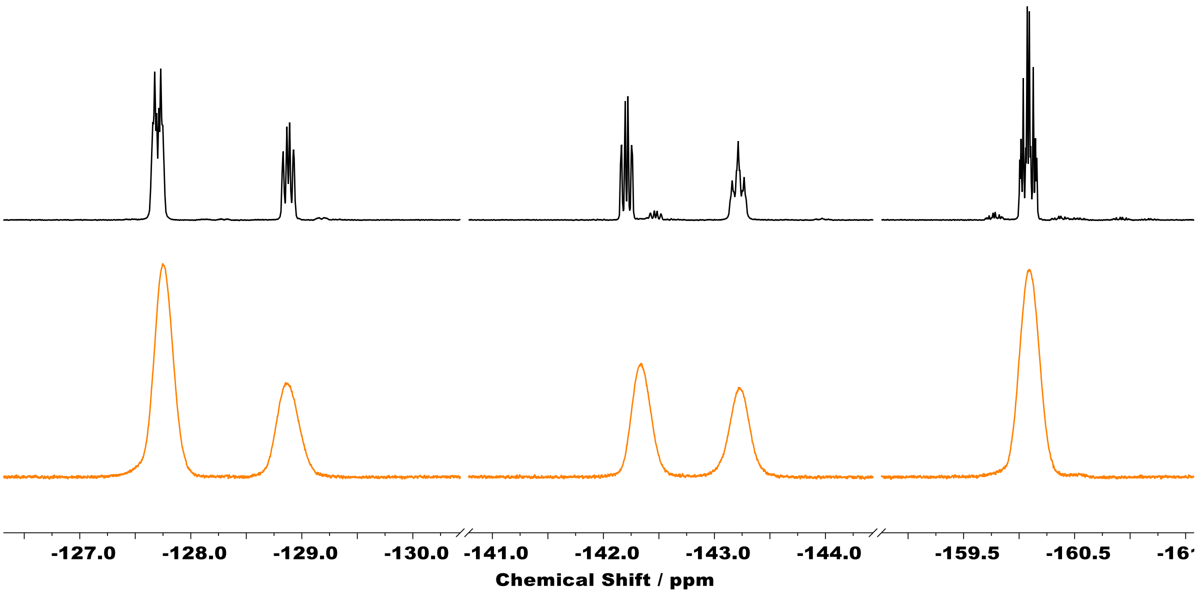

Fig.S21 Stacked ^{19}F NMR (376 MHz, $CDCl_3$) spectra of post-polymerisation modification reaction between **Poly-Si** and **1**. Top: **1**; bottom: **Poly-1**.

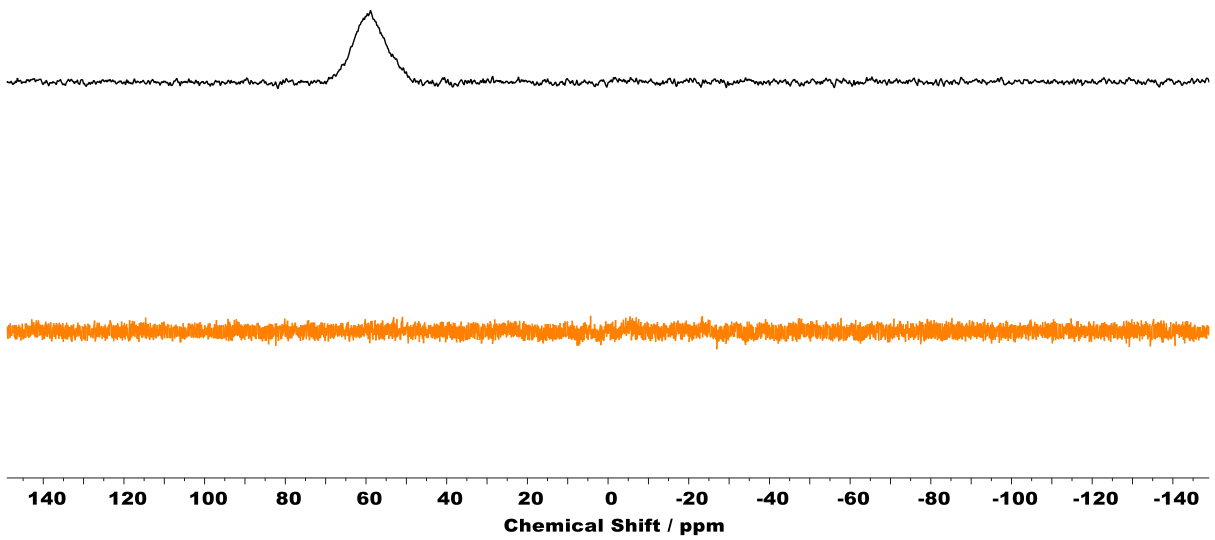

Fig.S22 Stacked ^{11}B NMR (128 MHz, $CDCl_3$) spectra of post-polymerisation modification reaction between **Poly-Si** and **1**. Top: **1**; bottom: **Poly-1**.

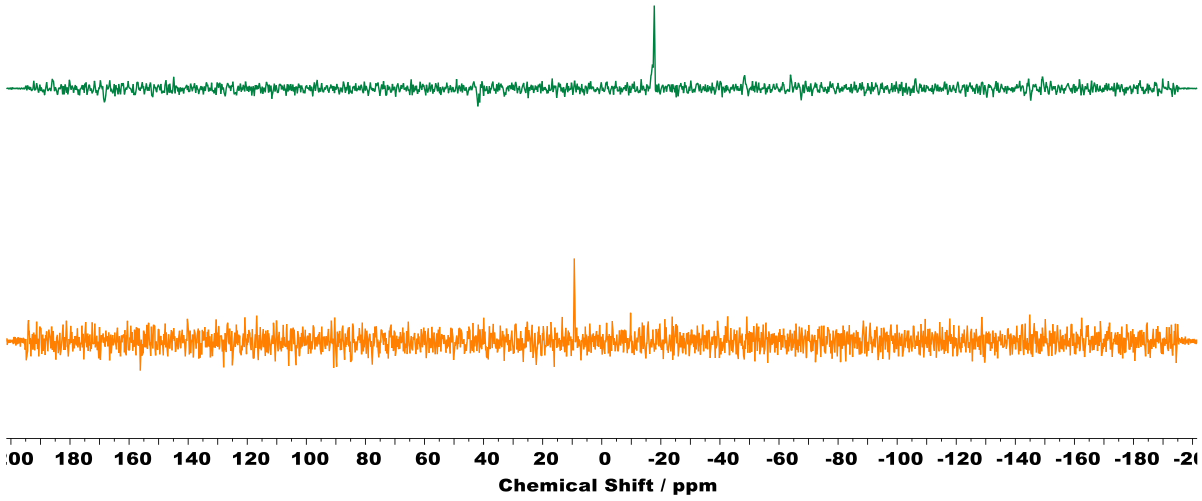

Fig.S23 Stacked ^{29}Si DEPT 90 NMR (97 MHz, $CDCl_3$) spectra of post-polymerisation modification reaction between **Poly-Si** and **1**. Top: **1**; bottom: **Poly-1**.

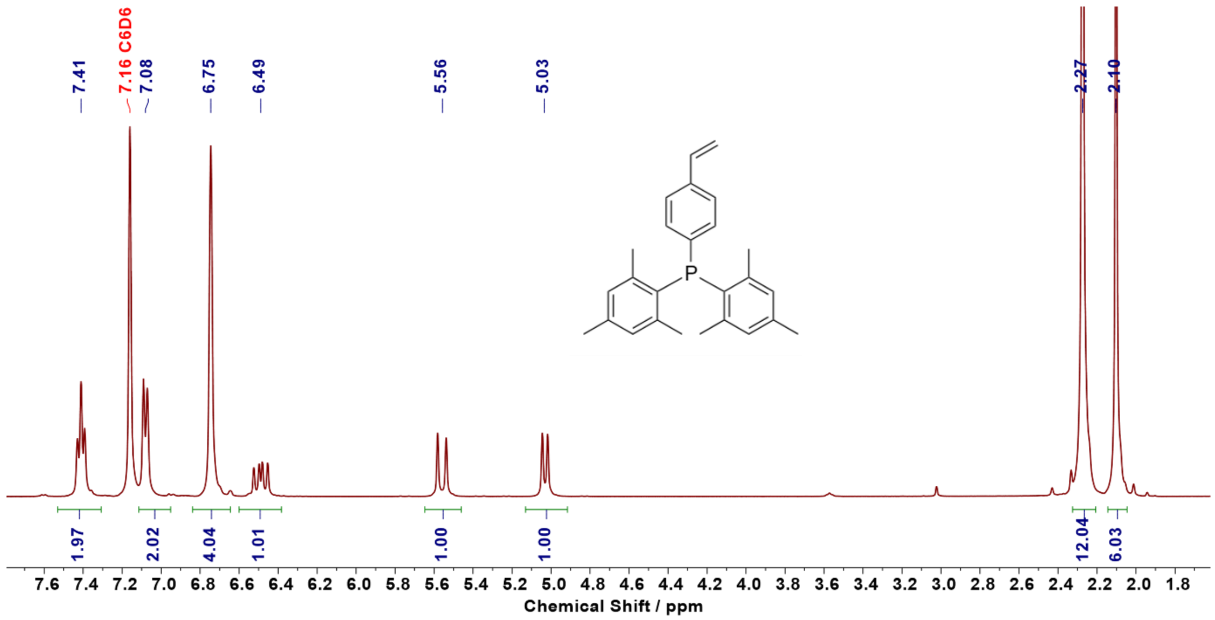

Fig.S24 ^{1}H NMR (400 MHz, C_6D_6) spectra of **2**.

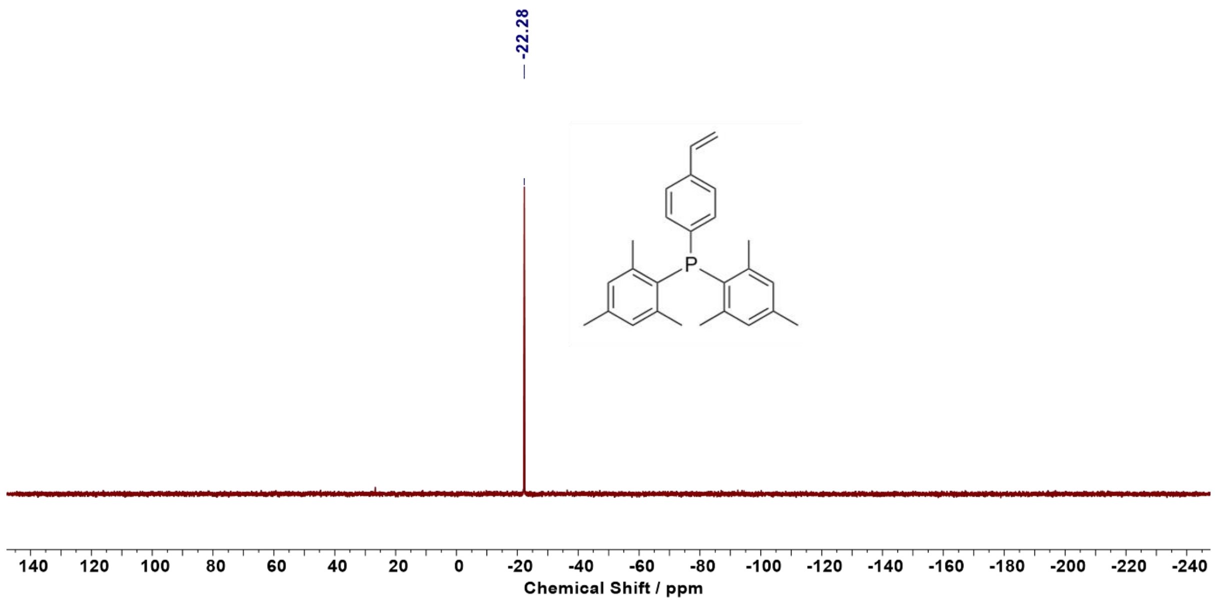

Fig.S25 ^{31}P NMR (162 MHz, C_6D_6) spectra of **2**.

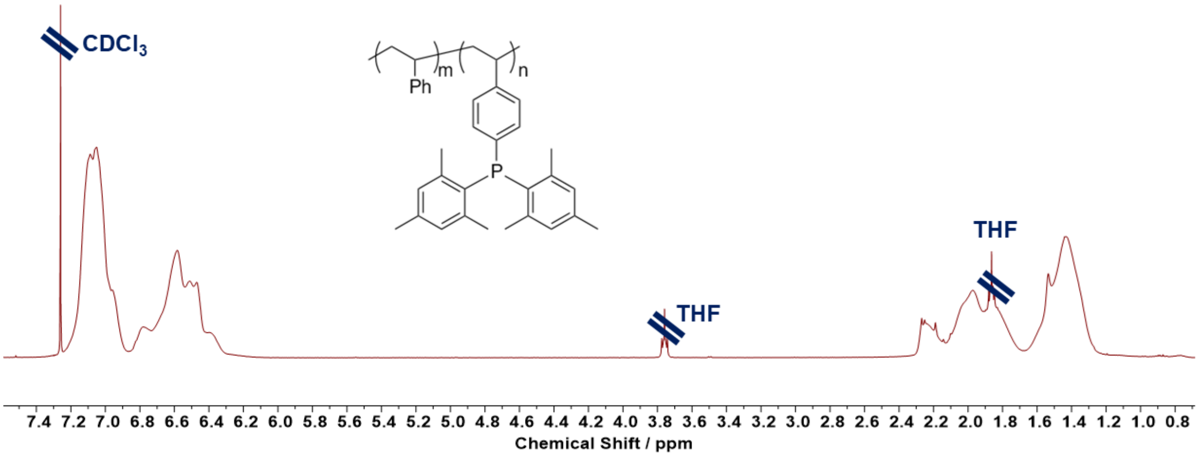

Fig.S26 ^{1}H NMR (400 MHz, $CDCl_3$) spectra of **Poly-2.**

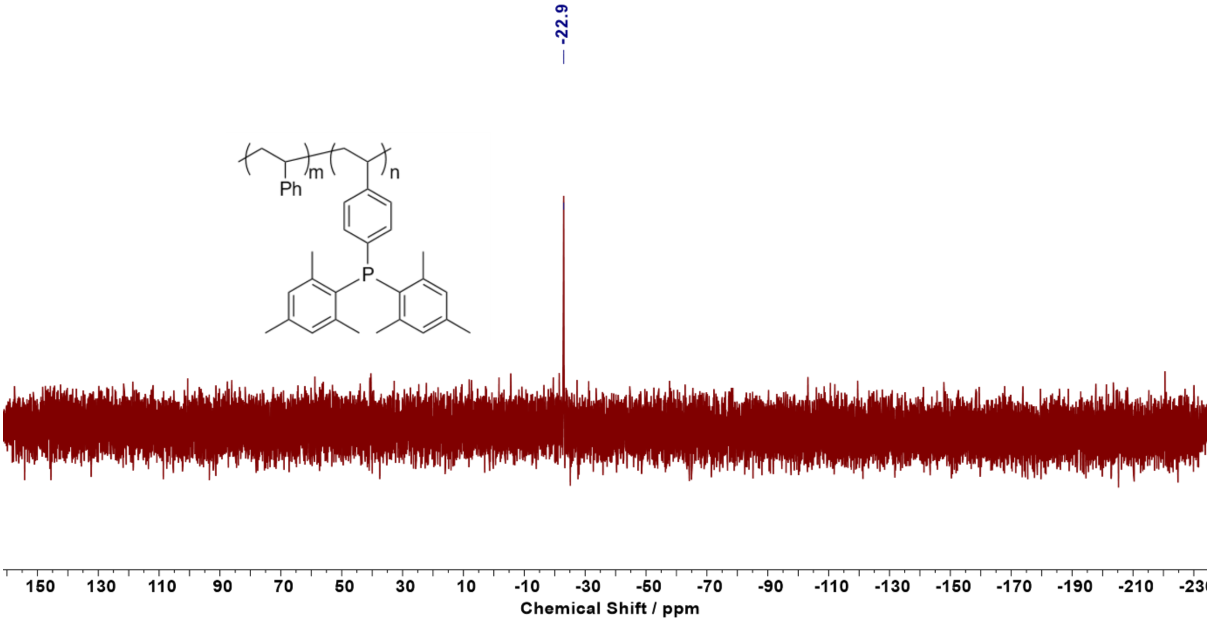

Fig.S27 ^{31}P NMR (162 MHz, $CDCl_3$) spectra of **Poly-2**.

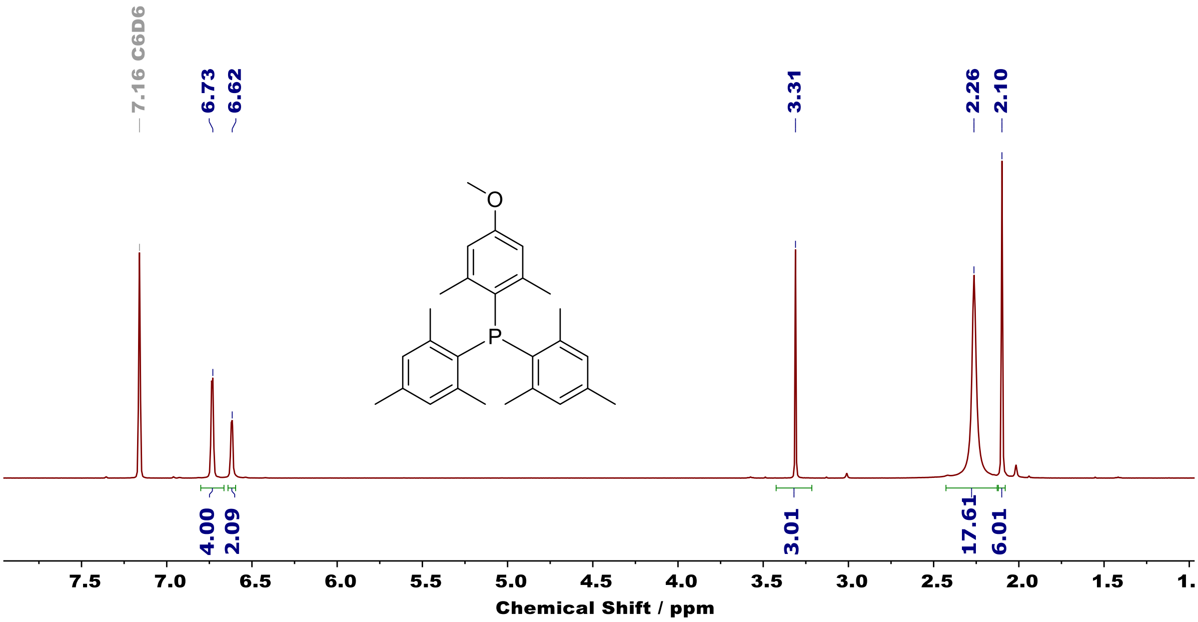

Fig.S28 ^{1}H NMR (400 MHz, C_6D_6) spectra of **3**.

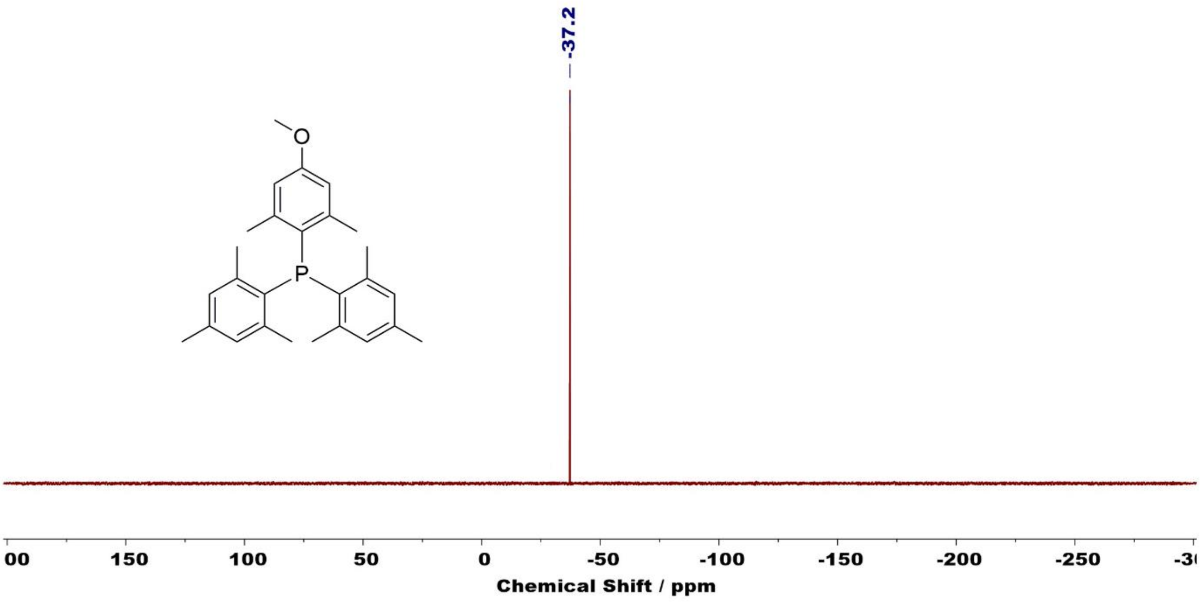

Fig.S29 ^{31}P NMR (162 MHz, C_6D_6) spectra of **3**.

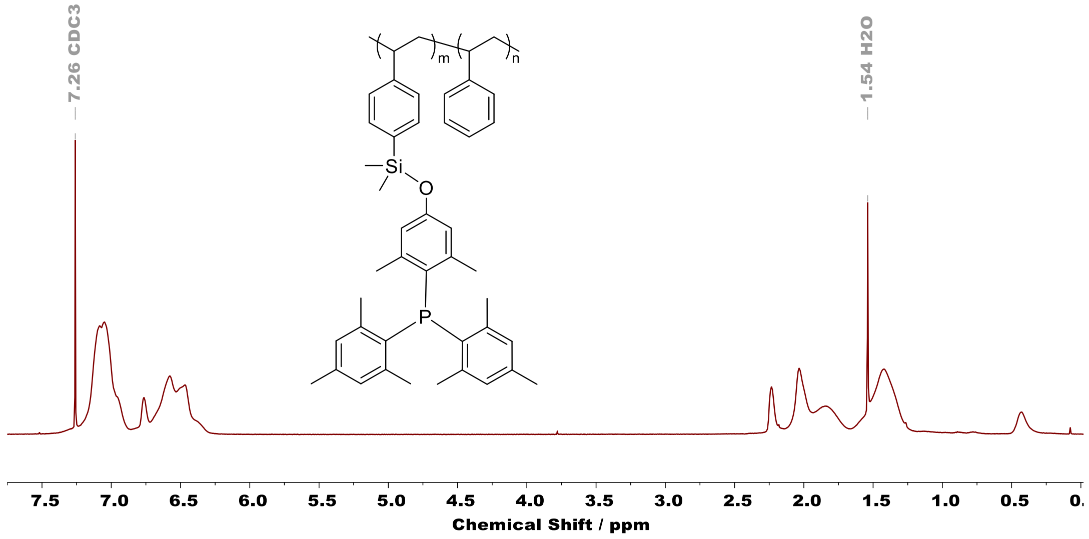

Fig.S30 ^{1}H NMR (400 MHz, $CDCl_3$) spectra of **Poly-3.**

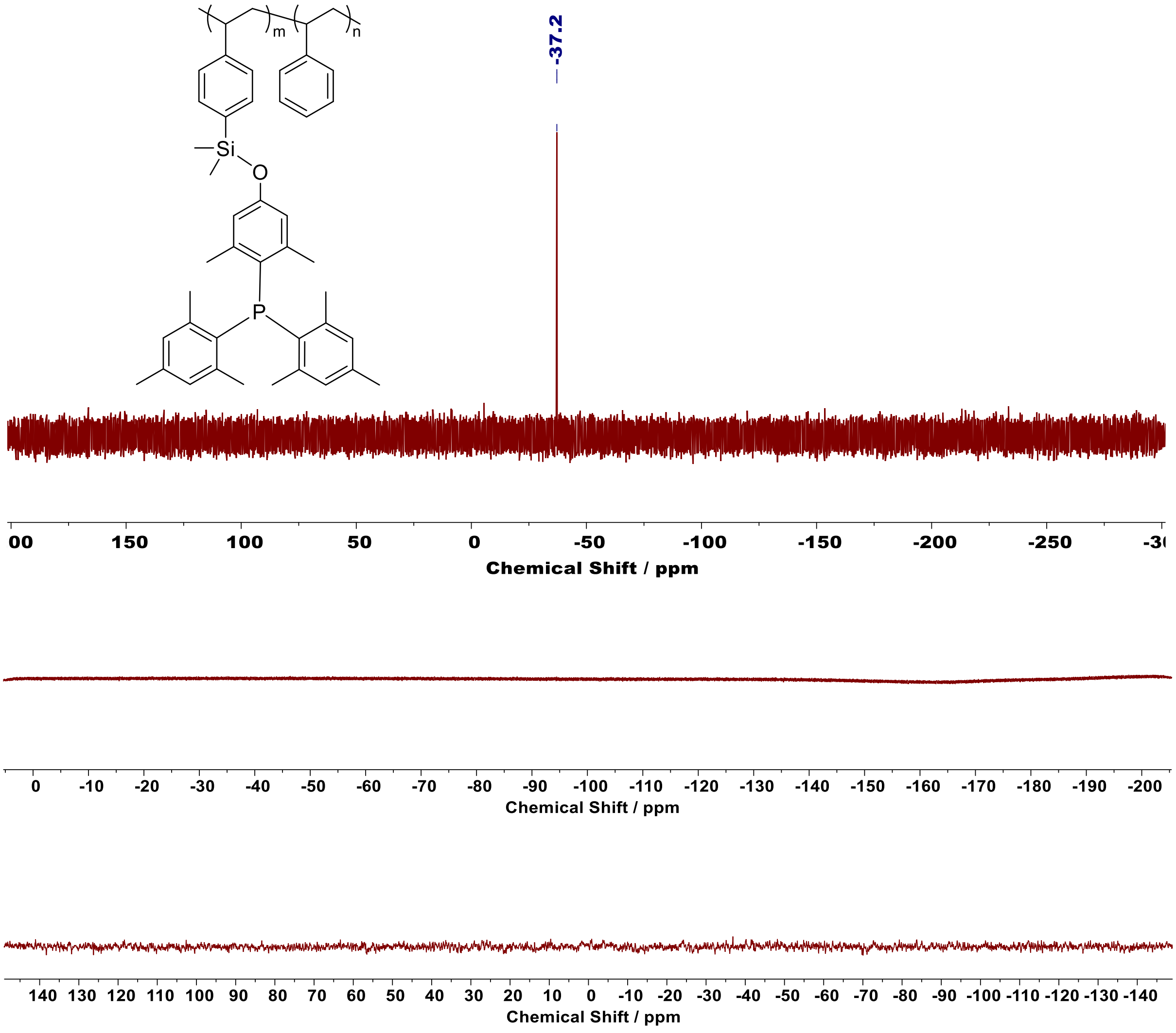

Fig.S31 Top: ^{31}P NMR (400 MHz, $CDCl_3$), **middle:** ^{19}F NMR (376 MHz, $CDCl_3$) and **bottom:** ^{11}B NMR (128 MHz, $CDCl_3$) spectra of purified **Poly-3.**

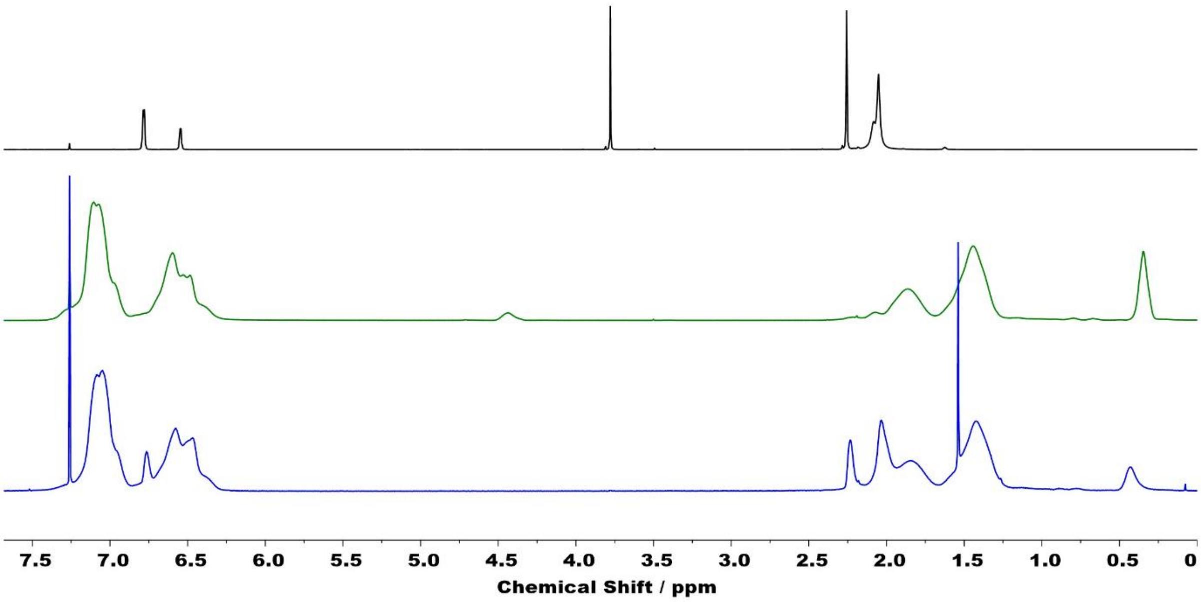

Fig.S32 Stacked ^{1}H NMR (400 MHz, $CDCl_3$) spectra of post-polymerisation modification reaction between **Poly-Si** and **3**. Top: **3**; middle: **Poly-Si**; bottom: **Poly-3**.

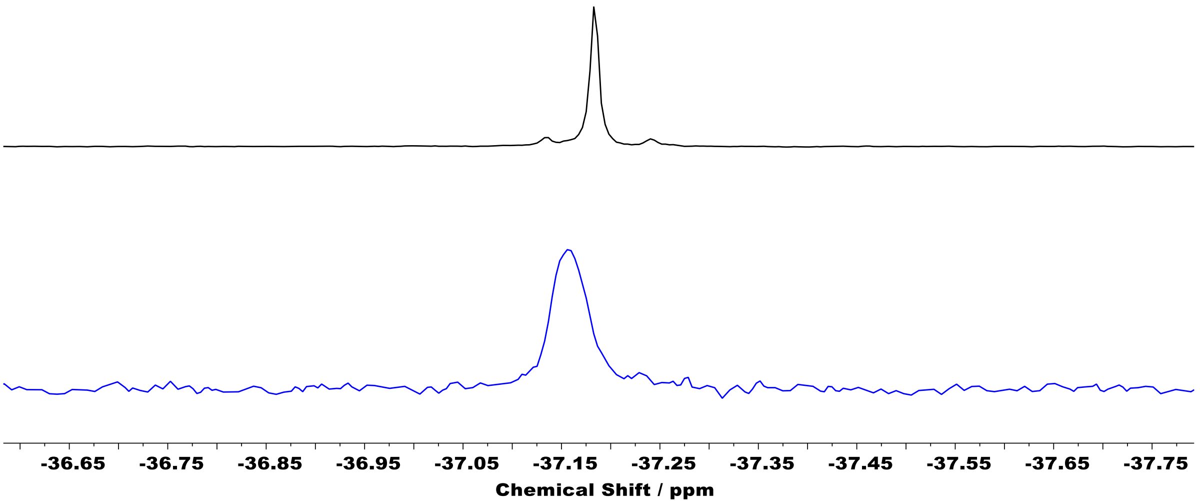

Fig.S33 Stacked ^{1}H NMR (400 MHz, $CDCl_3$) spectra of post-polymerisation modification reaction between **Poly-Si** and **3**. Top: **3**; bottom: **Poly-3**.

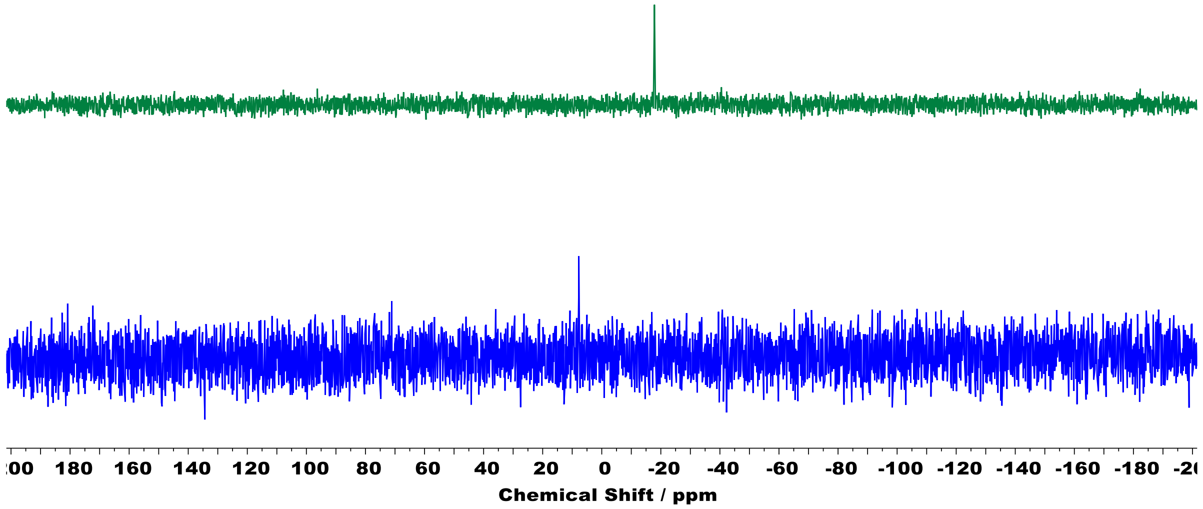

Fig.S34 Stacked ^{29}Si NMR (79 MHz, $CDCl_3$) spectra of post-polymerisation modification reaction between **Poly-Si** and **3**. Top: **3**; bottom: **Poly-3**.

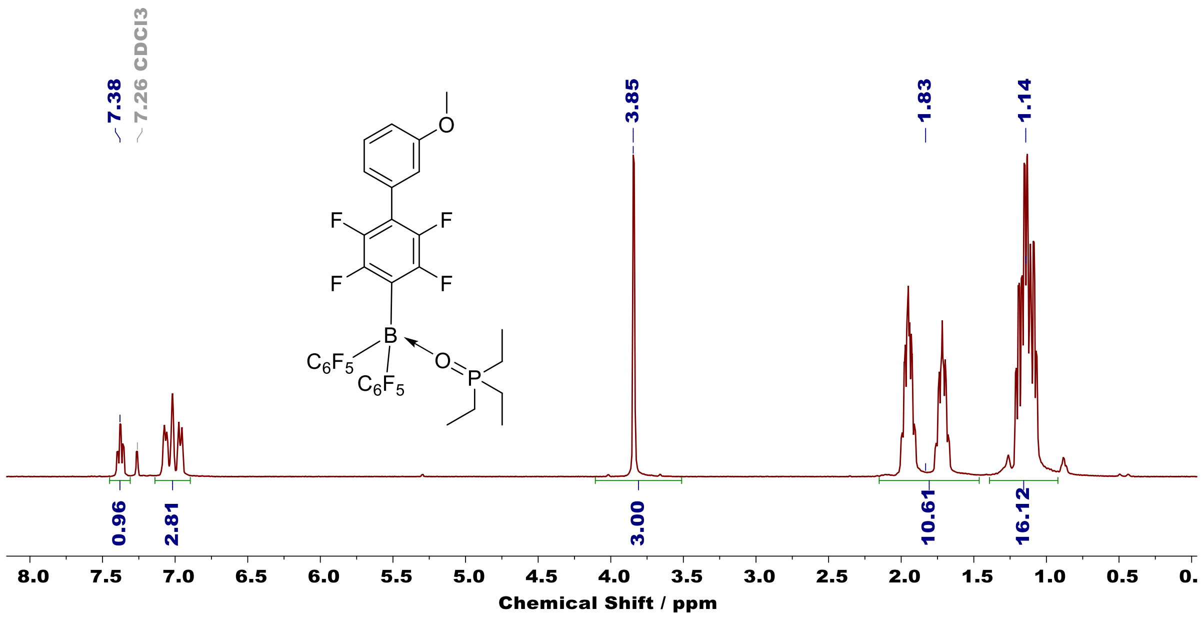

Fig.S35 ^{1}H NMR (400 MHz, $CDCl_3$) spectrum of mixture of **1** and triethylphosphine oxide.

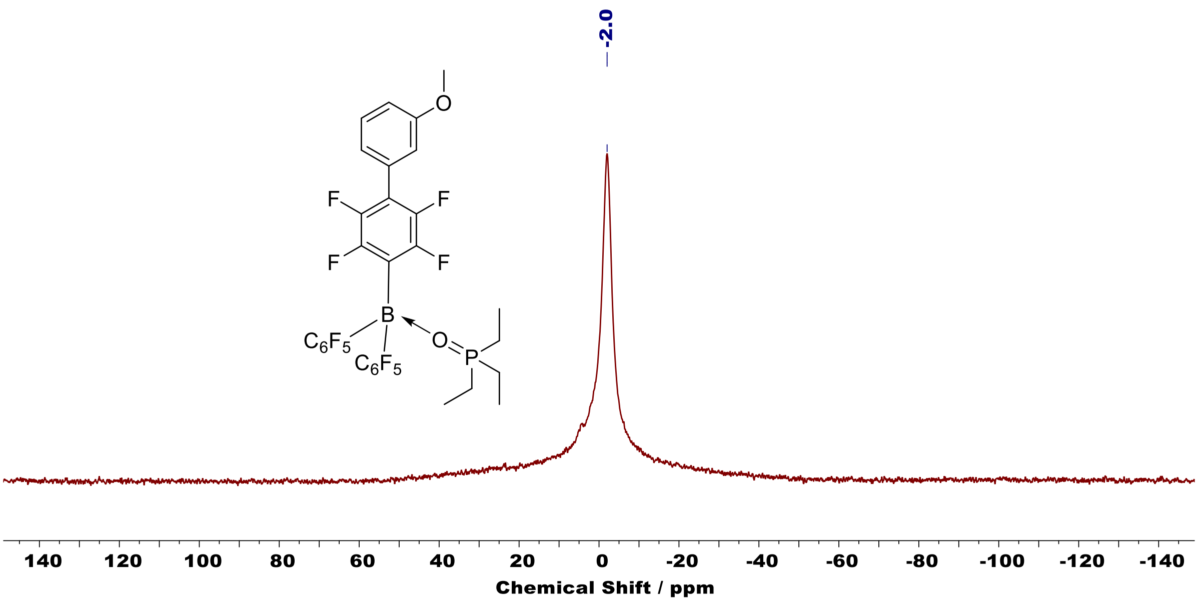

Fig.S36 ^{11}B NMR (128 MHz, $CDCl_3$) spectrum of mixture of **1** and triethylphosphine oxide.

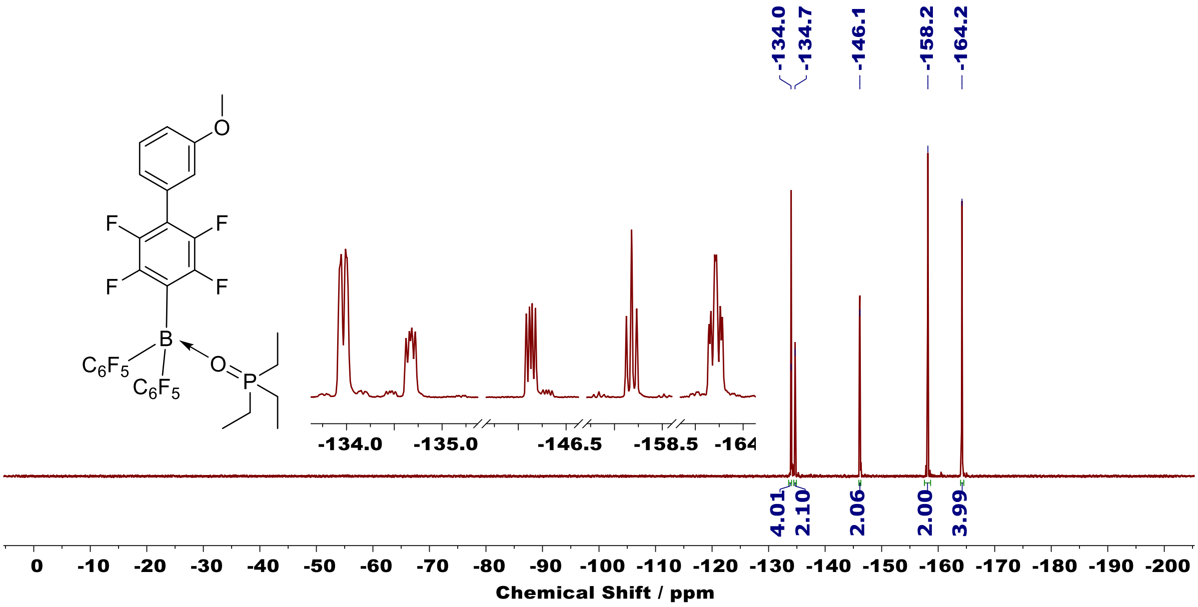

Fig.S37 ^{19}F NMR (376 MHz, $CDCl_3$) spectrum of mixture of **1** and triethylphosphine oxide.

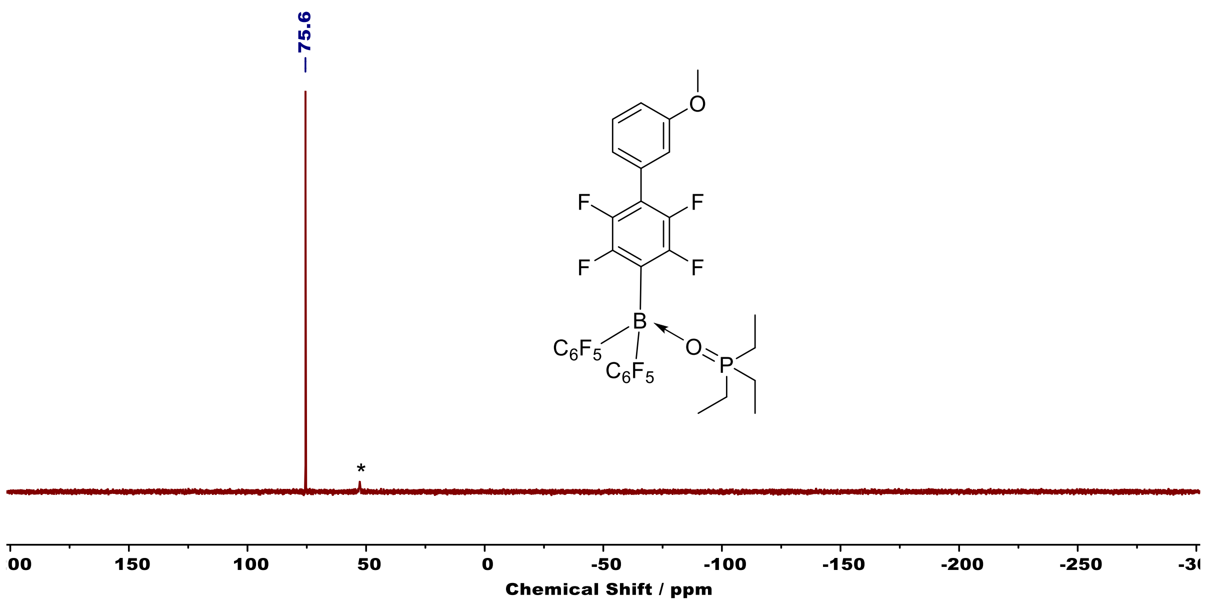

Fig.S38 ^{31}P NMR (162 MHz, $CDCl_3$) spectrum of mixture of **1** and triethylphosphine oxide. * slightly excess of free triethylphosphine oxide.

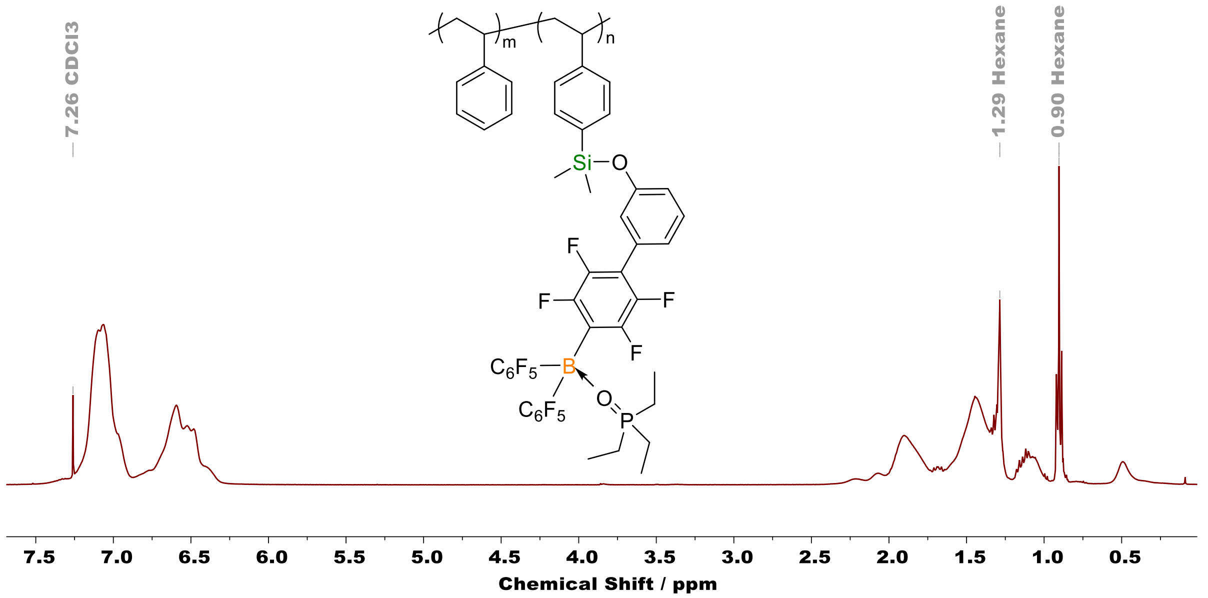

Fig.S39 ^{1}H NMR (400 MHz, $CDCl_3$) spectrum of mixture of **Poly-1** and triethylphosphine oxide.

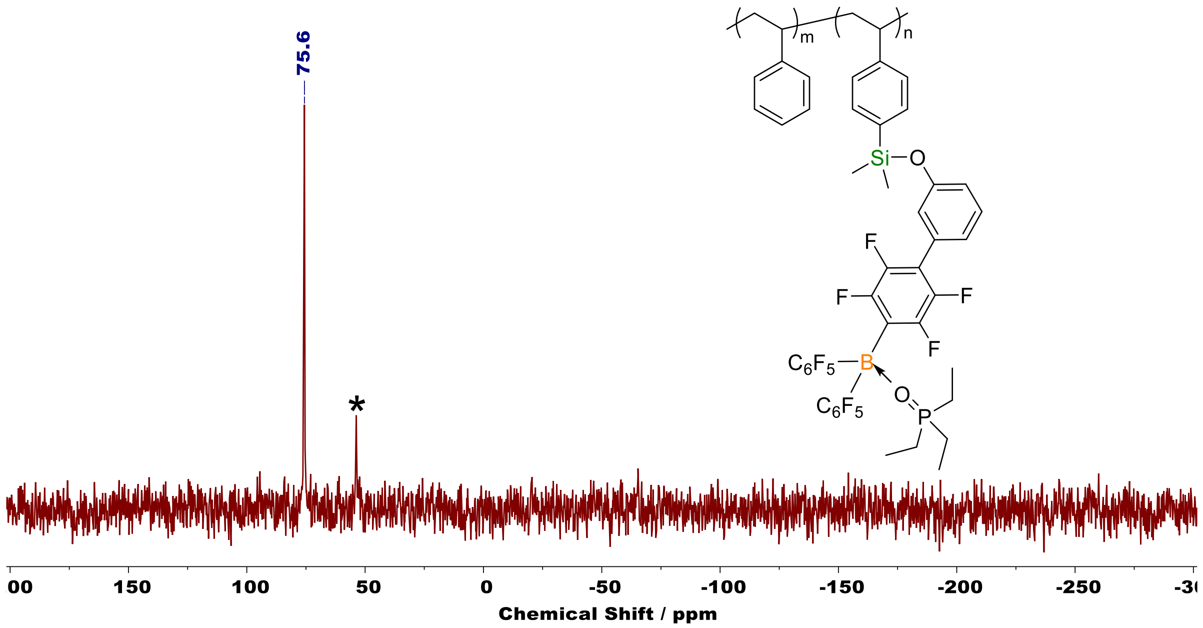

Fig.S40 ^{31}P NMR (162 MHz, CDCl$_3$) spectrum of mixture of **Poly-1** and triethylphosphine oxide. * slightly excess of free triethylphosphine oxide.

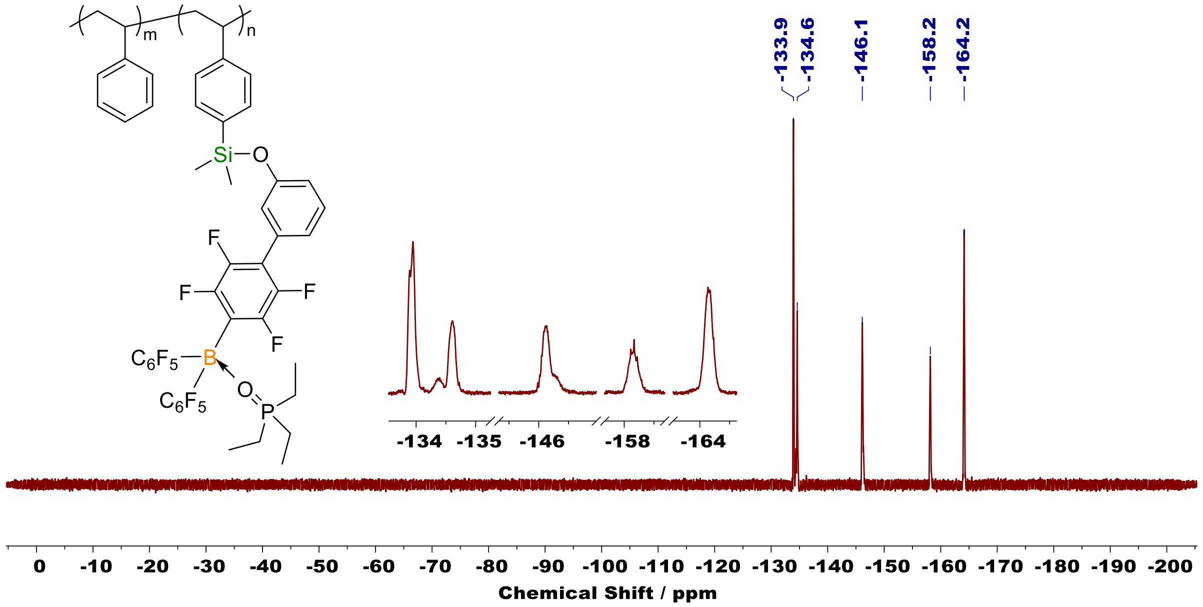

Fig.S41 ^{19}F NMR (376 MHz, CDCl$_3$) spectrum of mixture of **Poly-1** and triethylphosphine oxide.

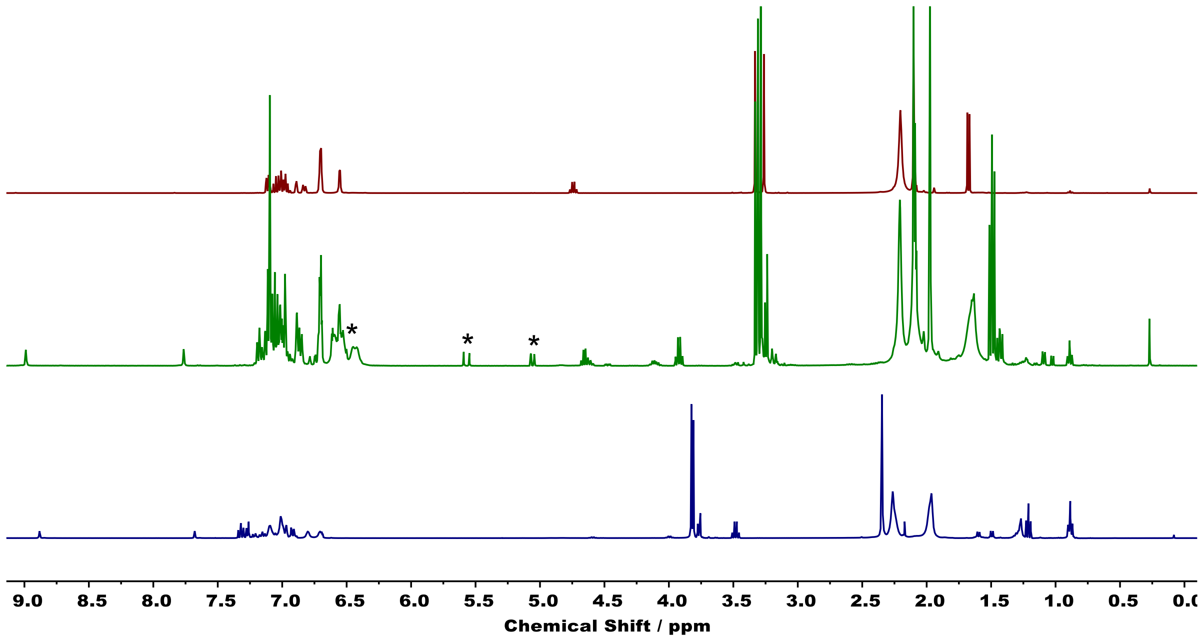

Fig.S42 Stacked ^{1}H NMR (400 MHz, top & middle: toluene-d_8, bottom: $CDCl_3$) spectra of dehydrohalogenation of (1-chloroetheyl)benzene by **1** and **3**. Top: mixture of **1** and **3**; Middle: after RT overnight. * vinyl peaks of generated styrene; Bottom: purified $[Mes_3PH]^+[ClB(C_6F_5)_3]^-$.

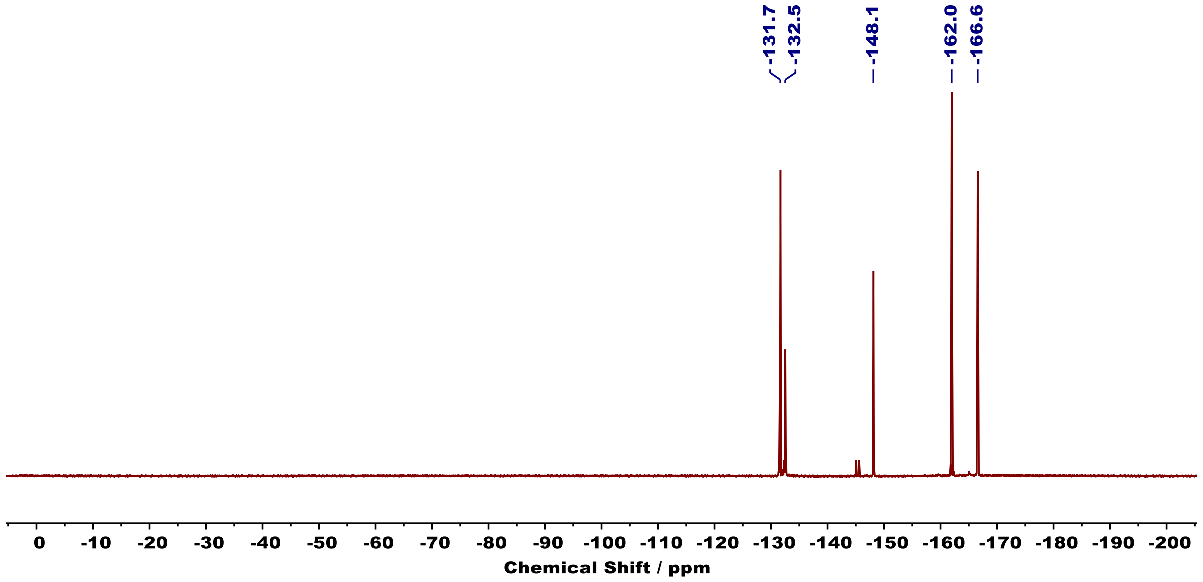

Fig.S43 ^{19}F NMR (376 MHz, $CDCl_3$) spectra of the purified product ($[Mes_3PH]^+[ClB(C_6F_5)_3]^-$) from dehydrohalogenation of (1-chloroetheyl)benzene by **1** and **3**.

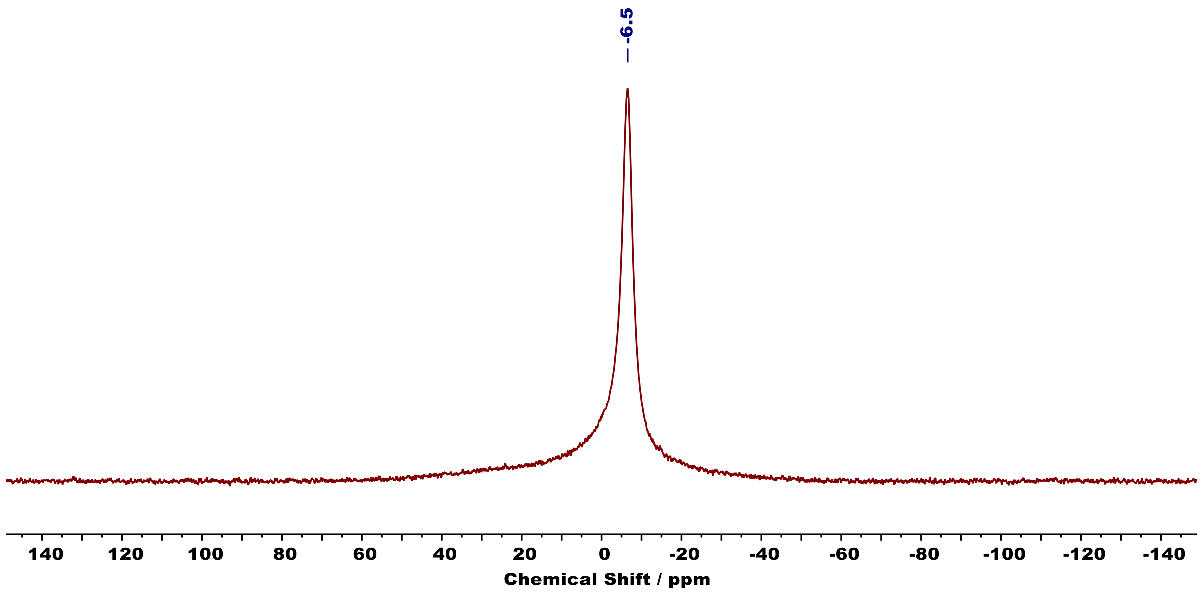

Fig.S44 ^{11}B NMR (128 MHz, $CDCl_3$) spectra of the purified product ($[Mes_3PH]^+[ClB(C_6F_5)_3]^-$) from dehydrohalogenation of (1-chloroetheyl)benzene by **1** and **3**.

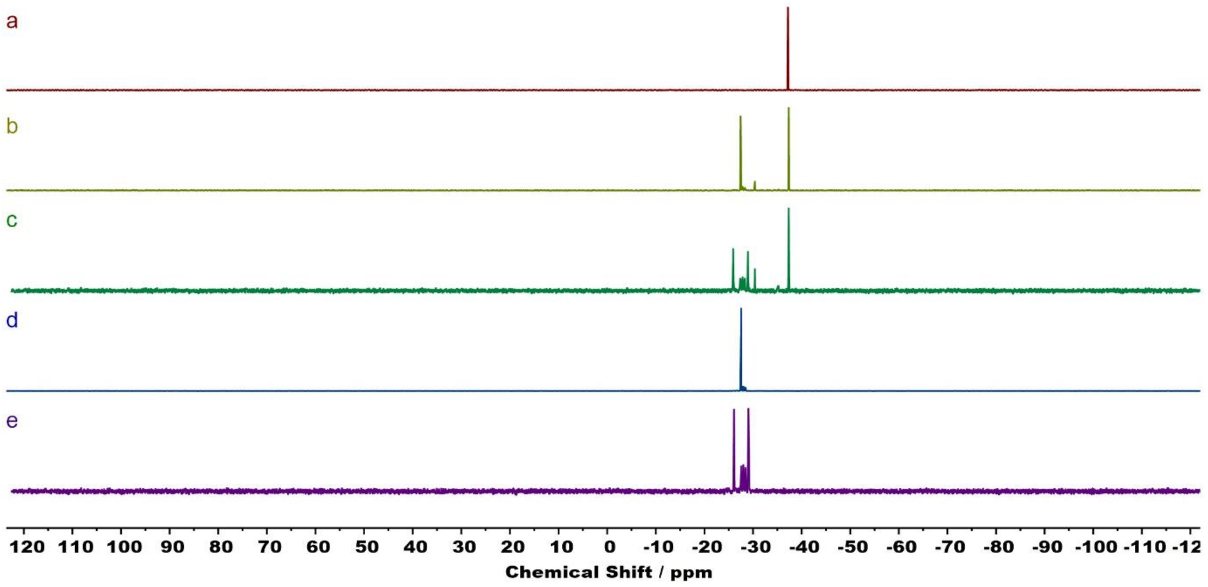

Fig.S45 Stacked ^{31}P NMR (162 MHz, **a-c**: toluene-d_8, **d-e**: $CDCl_3$) spectra of dehydrohalogenation of (1-chloroetheyl)benzene by **1** and **3**. **a**: mixture of **1** and **3**; **b** and **c**: proton decoupled and coupled spectra after RT overnight. Protonated product was 70.7 % by integral; **d** and **e**: proton decoupled and coupled spectra of the purified $[Mes_3PH]^+[ClB(C_6F_5)_3]^-$.

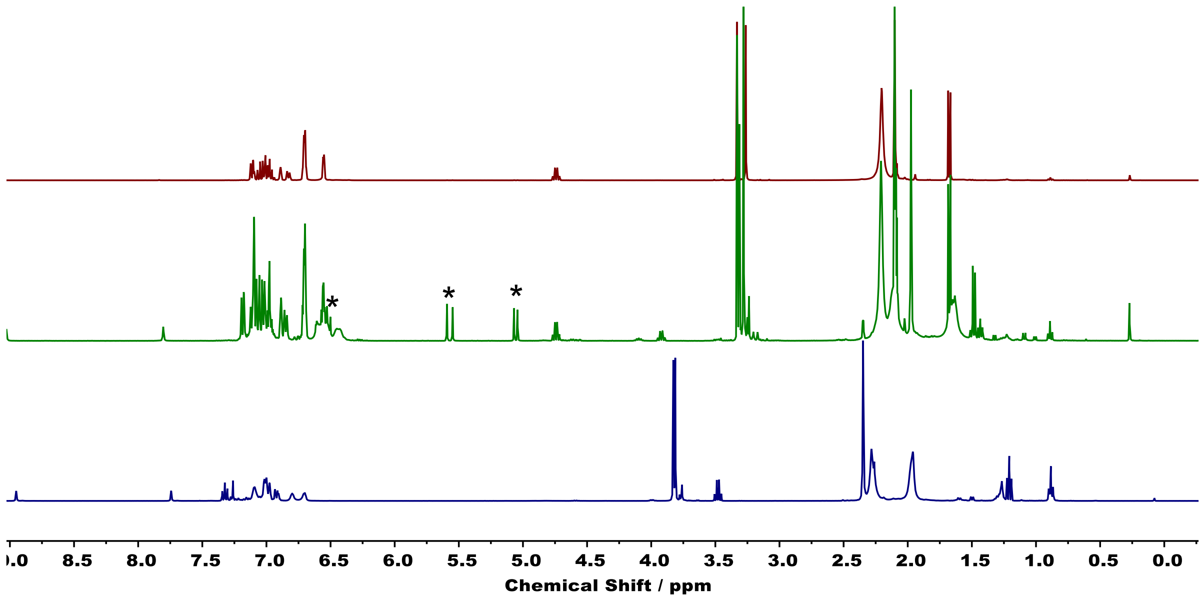

Fig.S46 Stacked ^{1}H NMR (400 MHz, top & middle: toluene-d_8, bottom: $CDCl_3$) spectra of dehydrohalogenation of (1-bromoetheyl)benzene by **1** and **3**. Top: before heating; Middle: after heating at 80 ^{o}C overnight. * vinyl peaks of generated styrene; Bottom: purified $[Mes_3PH]^+[BrB(C_6F_5)_3]^-$.

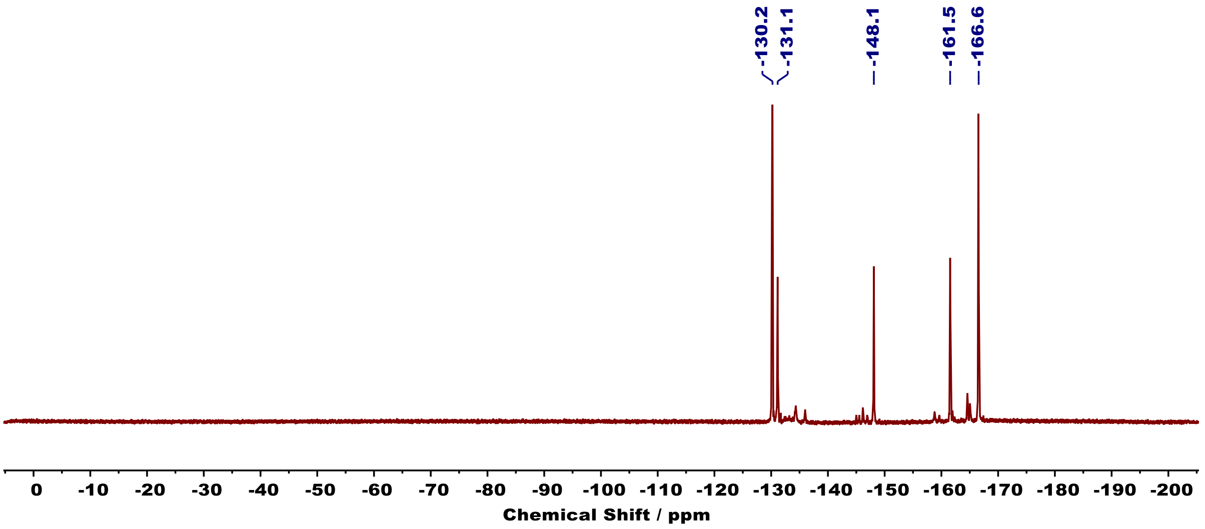

Fig.S47 ^{19}F NMR (376 MHz, $CDCl_3$) spectra of the purified product ($[Mes_3PH]^+[BrB(C_6F_5)_3]^-$) from dehydrohalogenation of (1-bromoetheyl)benzene by **1** and **3**.

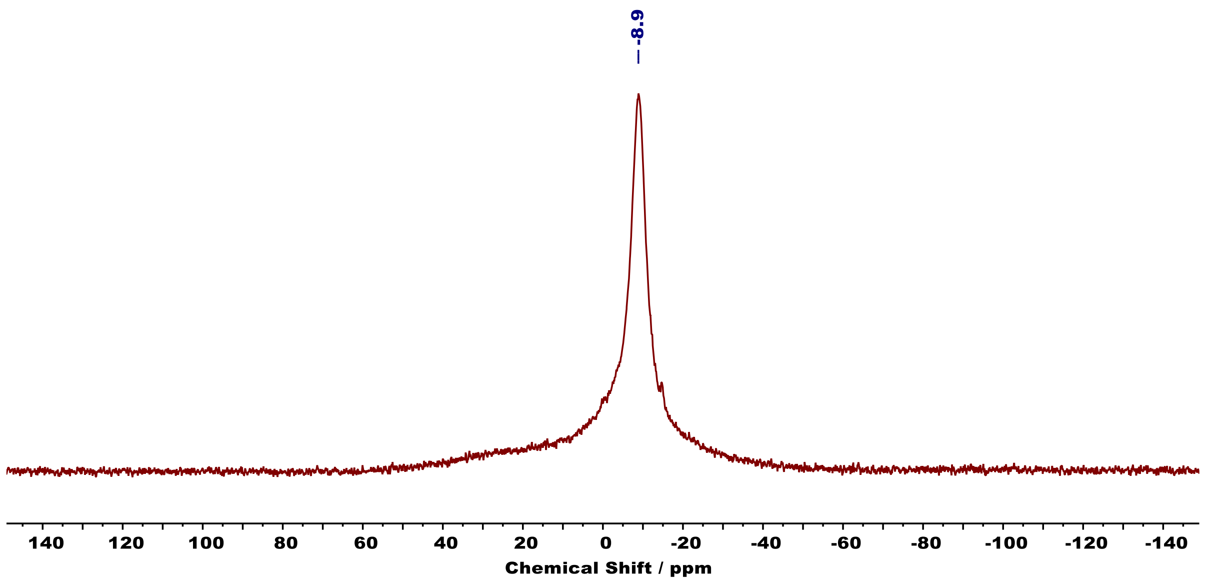

Fig.S48 ^{11}B NMR (128 MHz, $CDCl_3$) spectra of the purified product ($[Mes_3PH]^+[BrB(C_6F_5)_3]^-$) from dehydrohalogenation of (1-bromoetheyl)benzene by **1** and **3**.

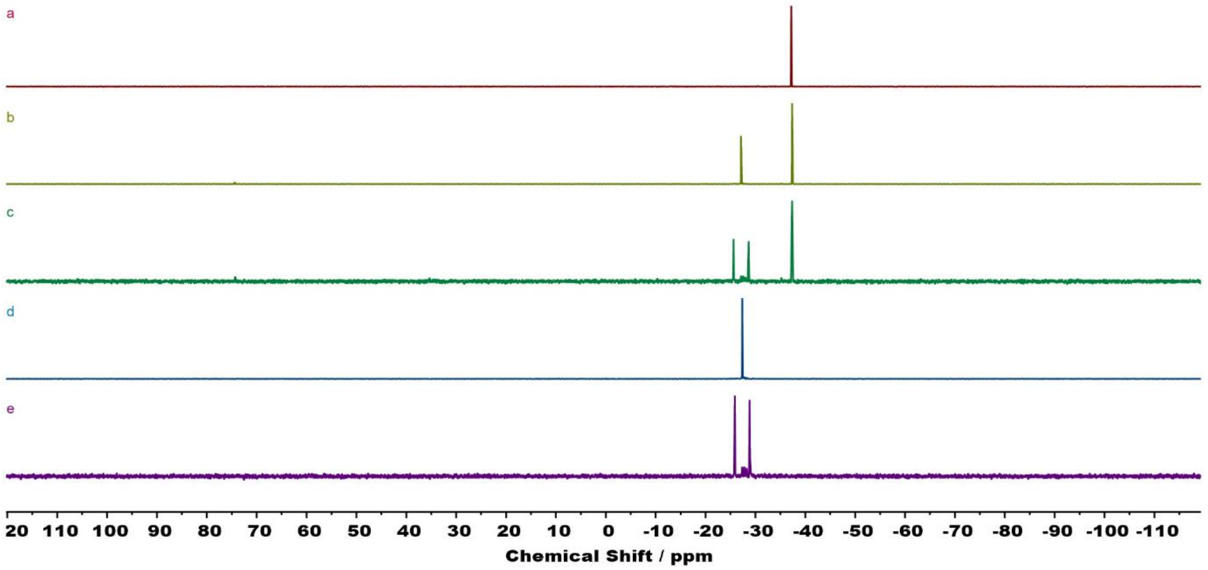

Fig.S49 Stacked ^{31}P NMR (162 MHz, **a-c**: toluene-d_8, **d-e**: $CDCl_3$) spectra of dehydrohalogenation of (1-bromoetheyl)benzene by **1** and **3**. **a**: before heating; **b** and **c**: proton decoupled and coupled spectra after heating at 80 °C overnight. Protonated product was 60.8 % by integral; **d** and **e**: proton decoupled and coupled spectra of the purified $[Mes_3PH]^+[BrB(C_6F_5)_3]^-$.

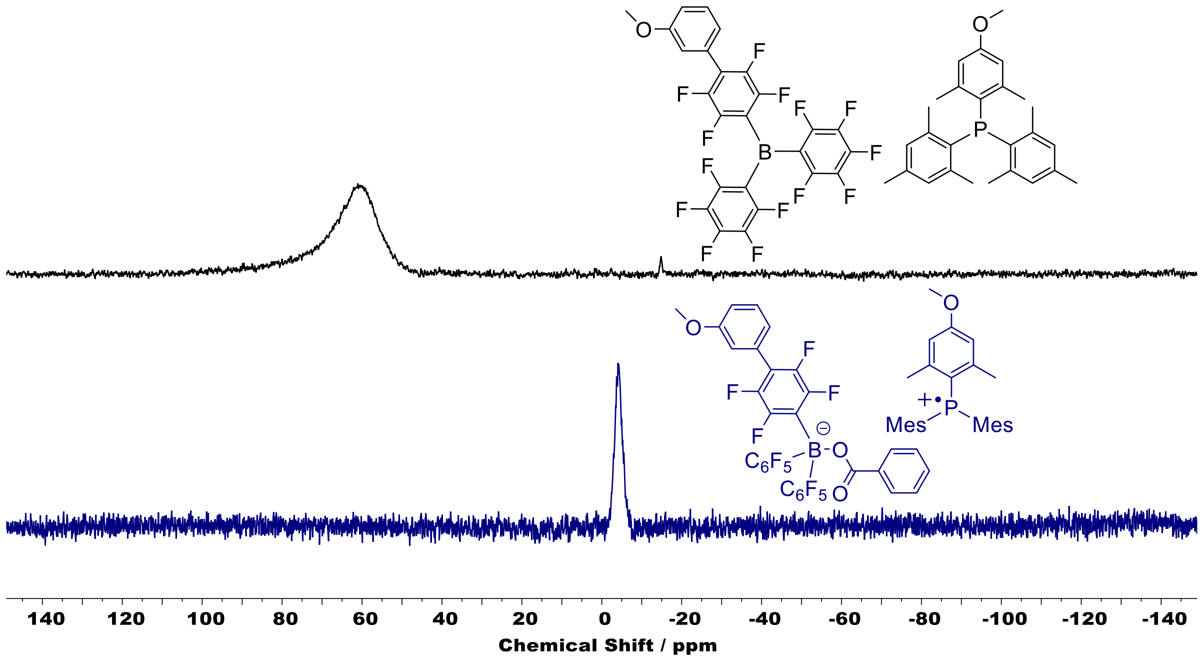

Fig.S50 Stacked ^{11}B NMR (128 MHz, $CDCl_3$) spectra of the activation of benzoyl peroxide by **1/3**. Top: Mixture of **1** and **3**; Bottom: after addition of benzoyl peroxide.

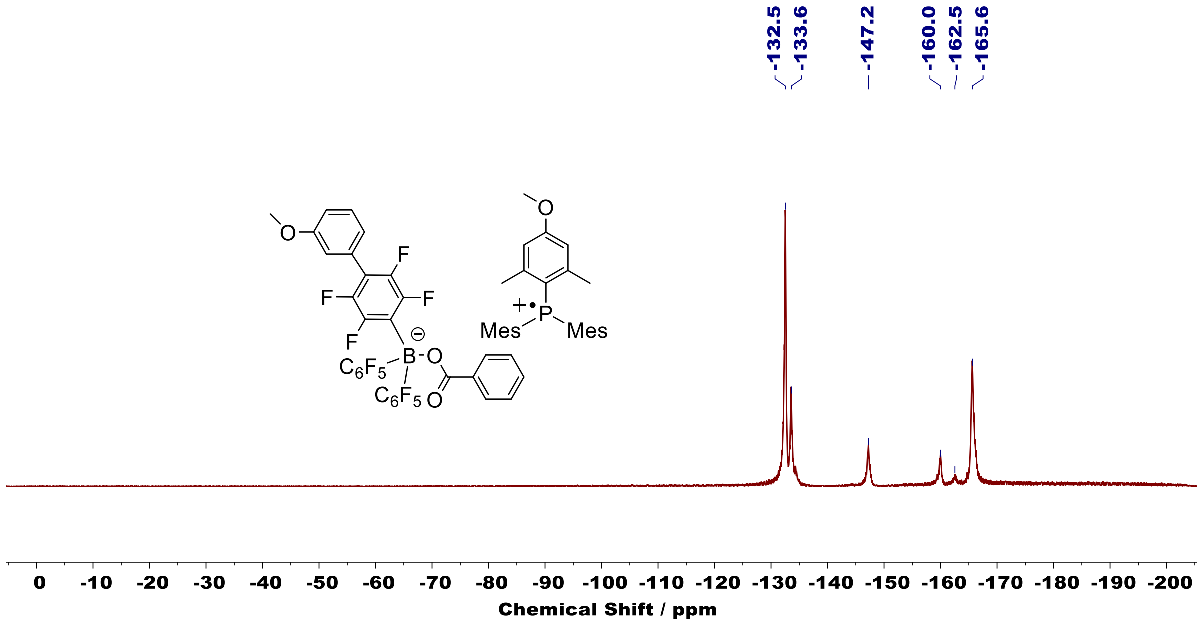

Fig.S51 Stacked ^{19}F NMR (376 MHz, $CDCl_3$) spectra of the activation of benzoyl peroxide by **1/3**.

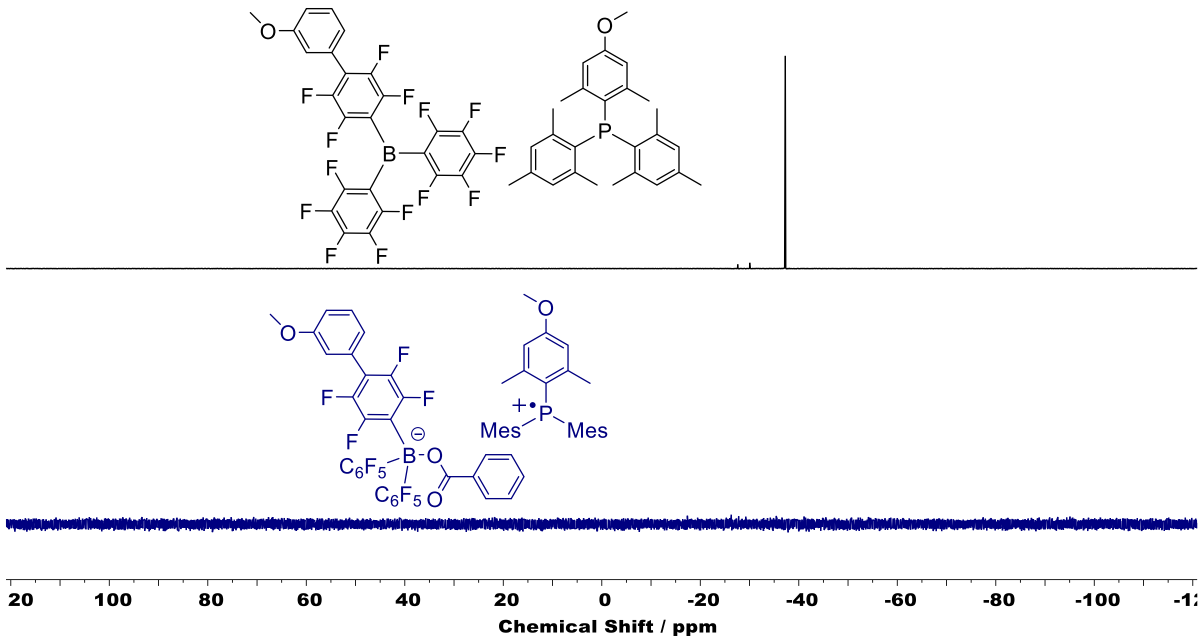

Fig.S52 Stacked ^{31}P NMR (162 MHz, $CDCl_3$) spectra of the activation of benzoyl peroxide by **1/3**. Top: Mixture of **1** and **3**; Bottom: after addition of benzoyl peroxide.

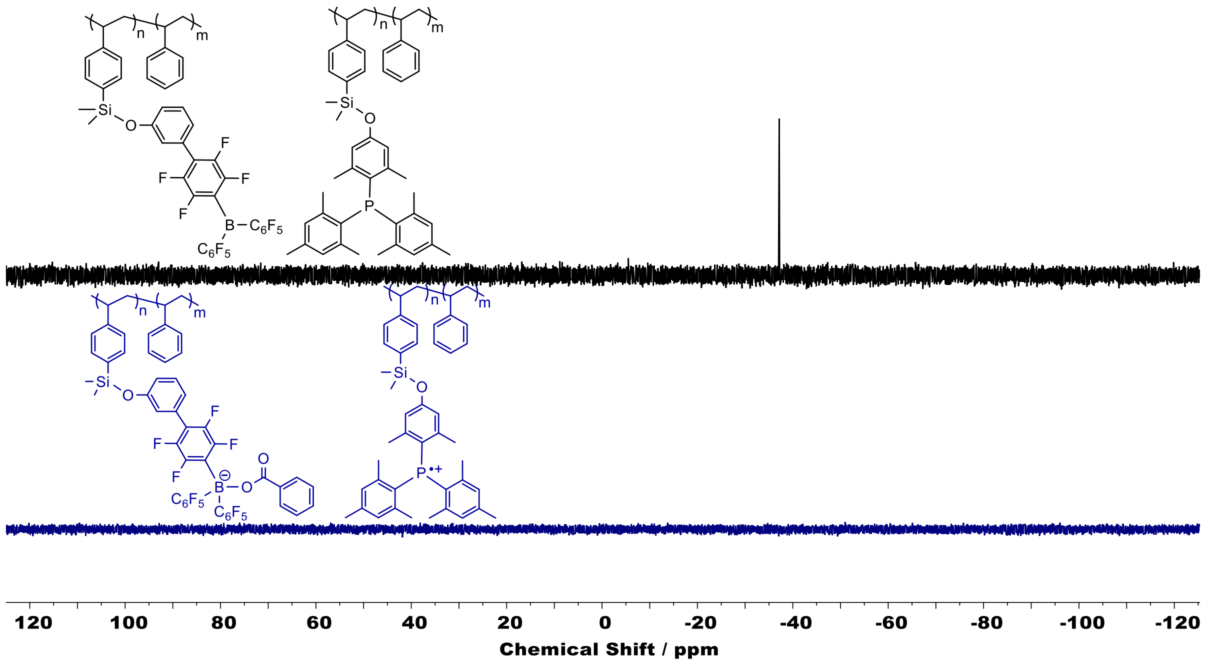

Fig.S53 Stacked ^{31}P NMR (162 MHz, $CDCl_3$) spectra of the activation of benzoyl peroxide by **Poly-1/Poly-3**. Top: Mixture of **Poly-1** and **Poly-3**; Bottom: after addition of benzoyl peroxide.

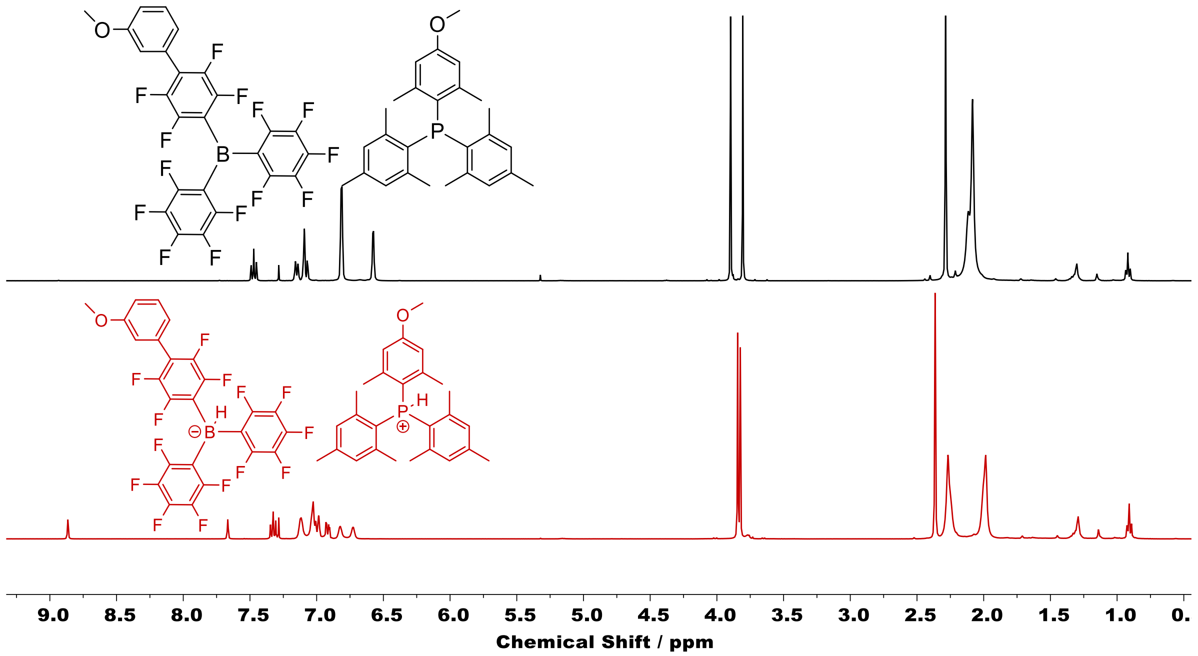

Fig.S54 Stacked ^{1}H NMR (400 MHz, $CDCl_3$) spectra of the dihydrogen cleavage by **1/3**. Top: Mixture of **1** and **3**; Bottom: purified product [**1**-H]$^-$/[**3**-H]$^+$.

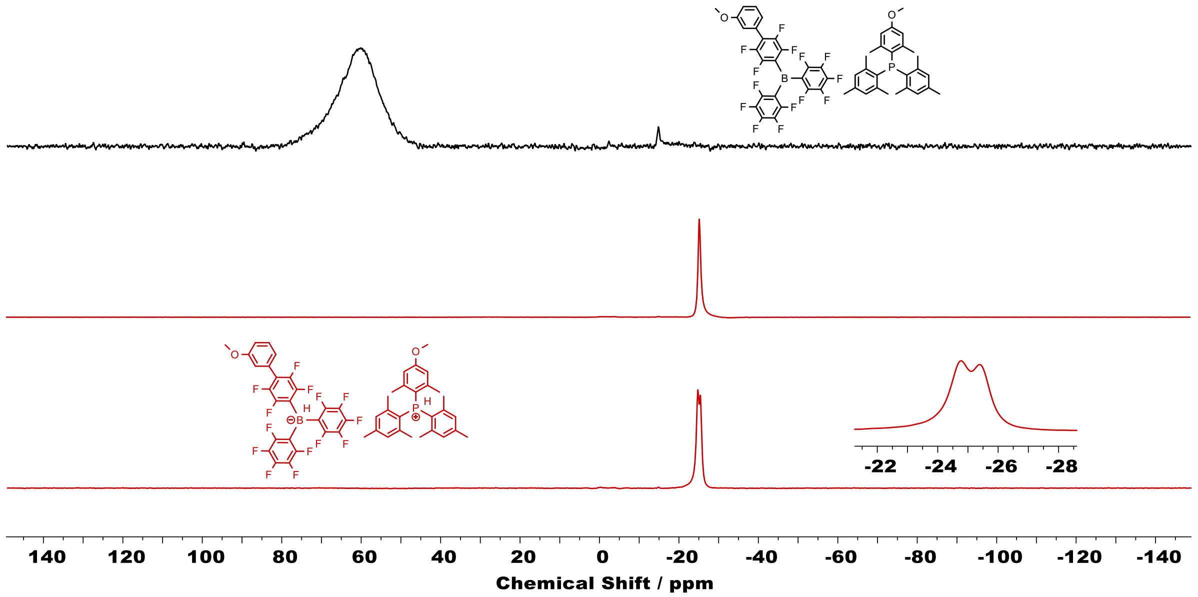

Fig.S55 Stacked ^{11}B NMR (128 MHz, $CDCl_3$) spectra of the dihydrogen cleavage by **1/3**. Top: Mixture of **1** and **3**; Middle: proton-decoupled spectrum and Bottom: proton-coupled spectrum of the purified product [**1**-H]$^-$/[**3**-H]$^+$.

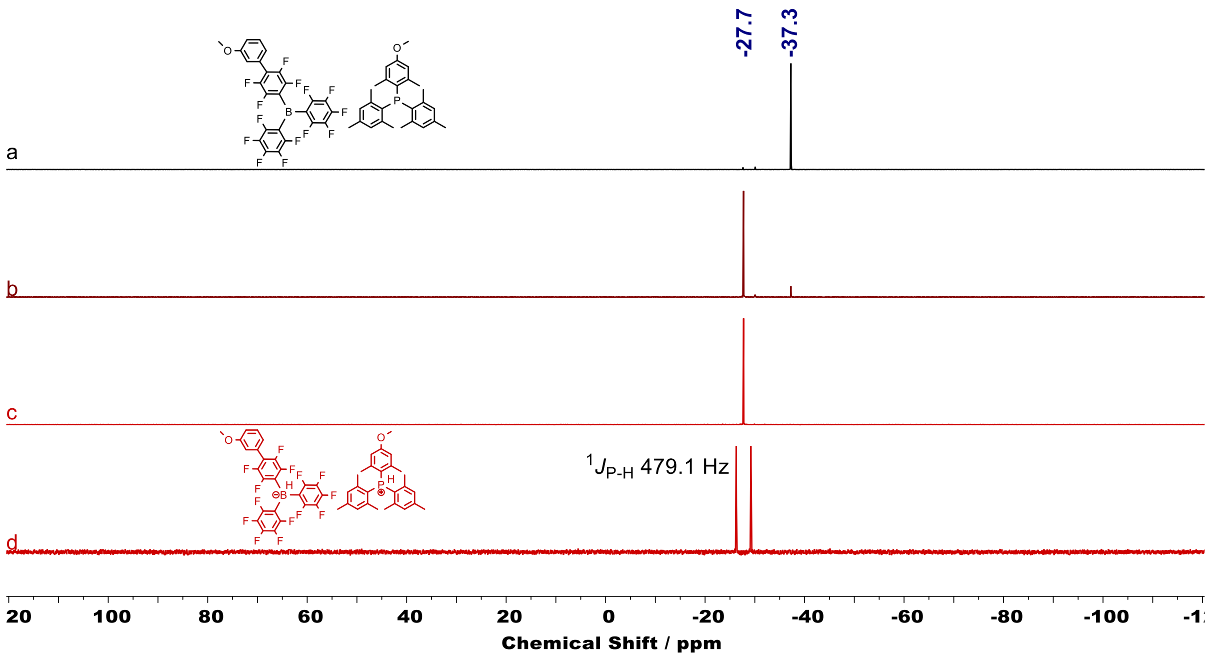

Fig.S56 Stacked ^{31}P NMR (162 MHz, $CDCl_3$) spectra of the dihydrogen cleavage by **1/3**. a: mixture of **1** and **3**; b: crude product; c: proton-decoupled and d: proton-coupled spectra of the purified product [**1**-H]$^-$/[**3**-H]$^+$.

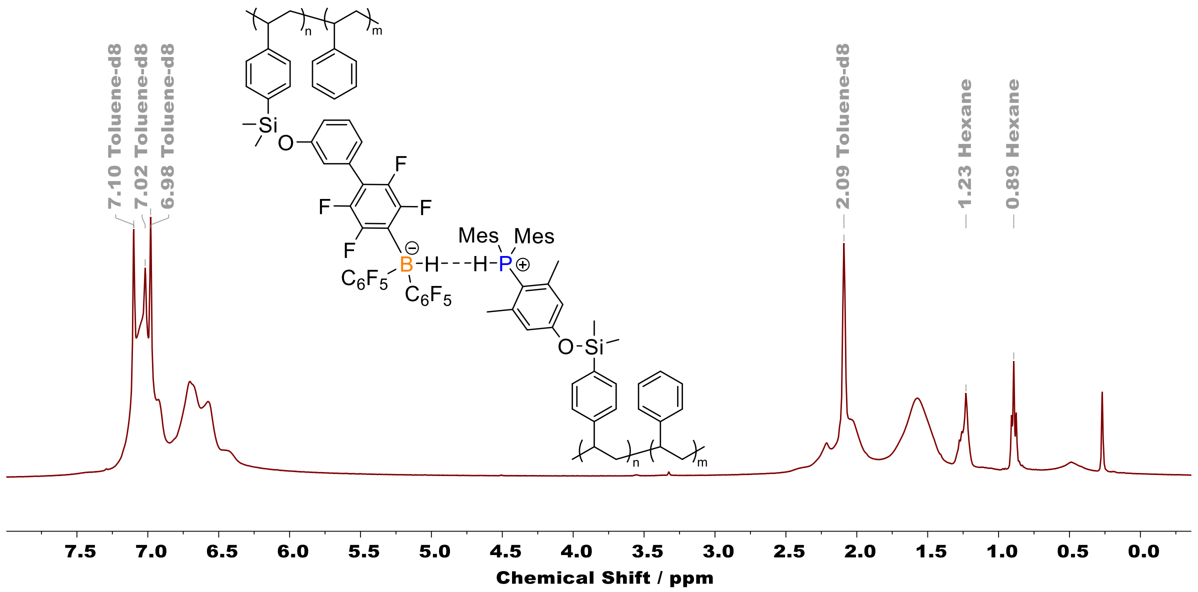

Fig.S57 ^{1}H NMR (400 MHz, toluene-d_8) spectra of the crude product from dihydrogen cleavage by **Poly-1/Poly-3**.

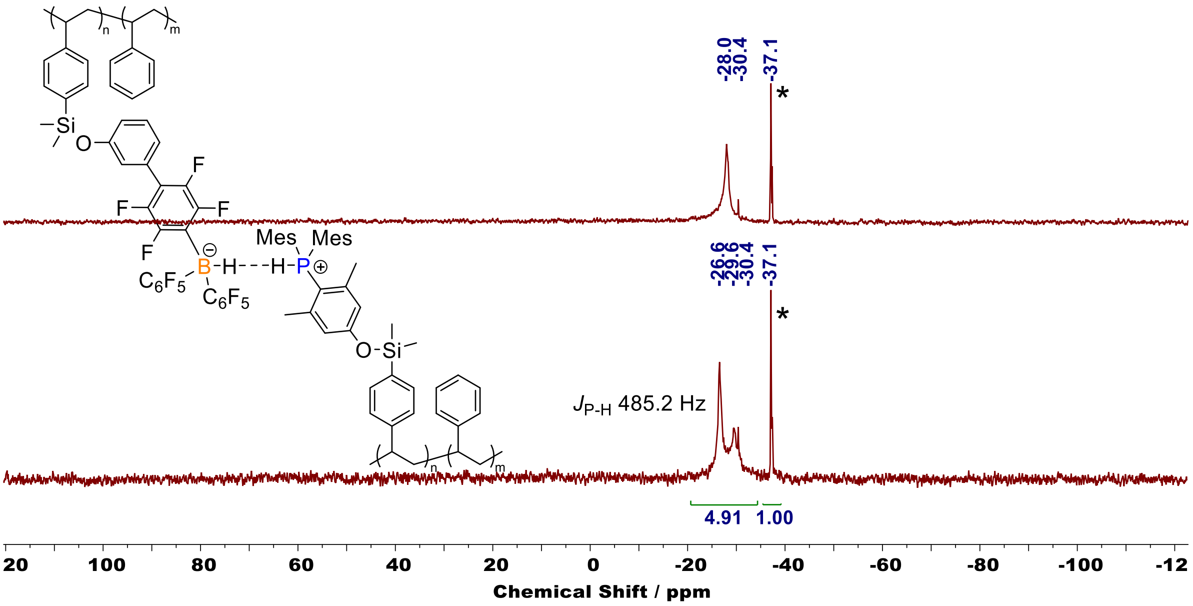

Fig.S58 Stacked proton-decoupled (top) and proton-coupled (bottom) ^{31}P NMR (162 MHz, toluene-d_8) spectra of the crude product from dihydrogen cleavage by **Poly-1/Poly-3**. * Unreacted **Poly-3.**

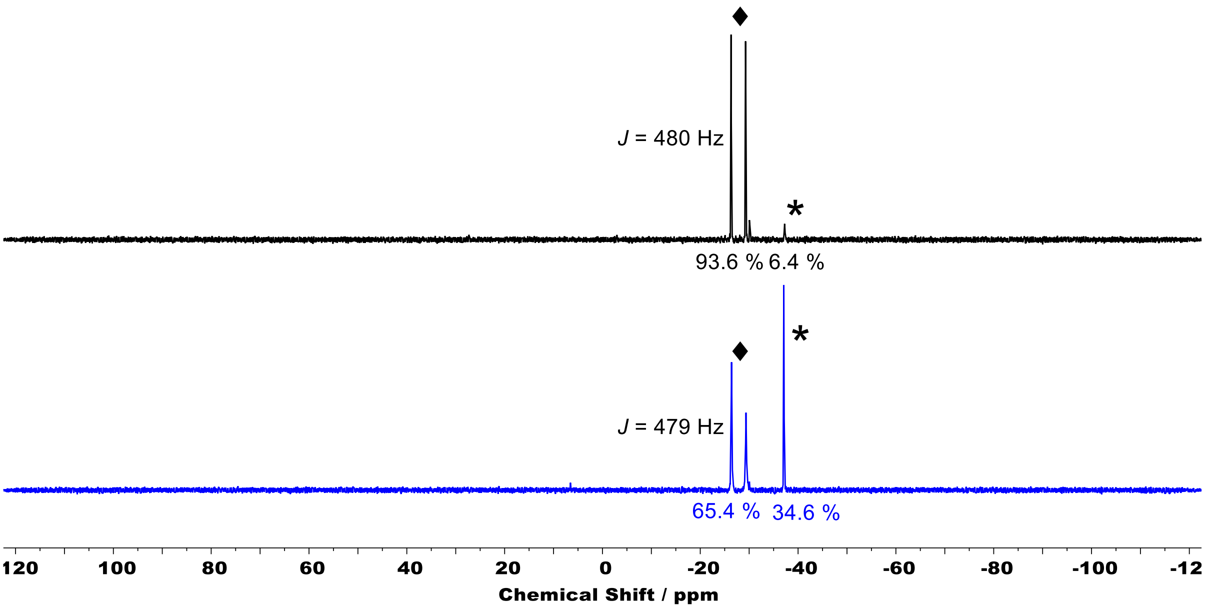

Fig. S59 ^{31}P NMR (162 MHz, $CDCl_3$) spectra of the crude products from the mixture of FRP/styrene/4-(trifluoromethyl)benzoate. Top: small molecular **1/3** and bottom: macromolecular **Poly-1/Poly-3** as catalysts. ♦Converted protonated phosphine; * unreacted phosphine **3** or **Poly-3**.

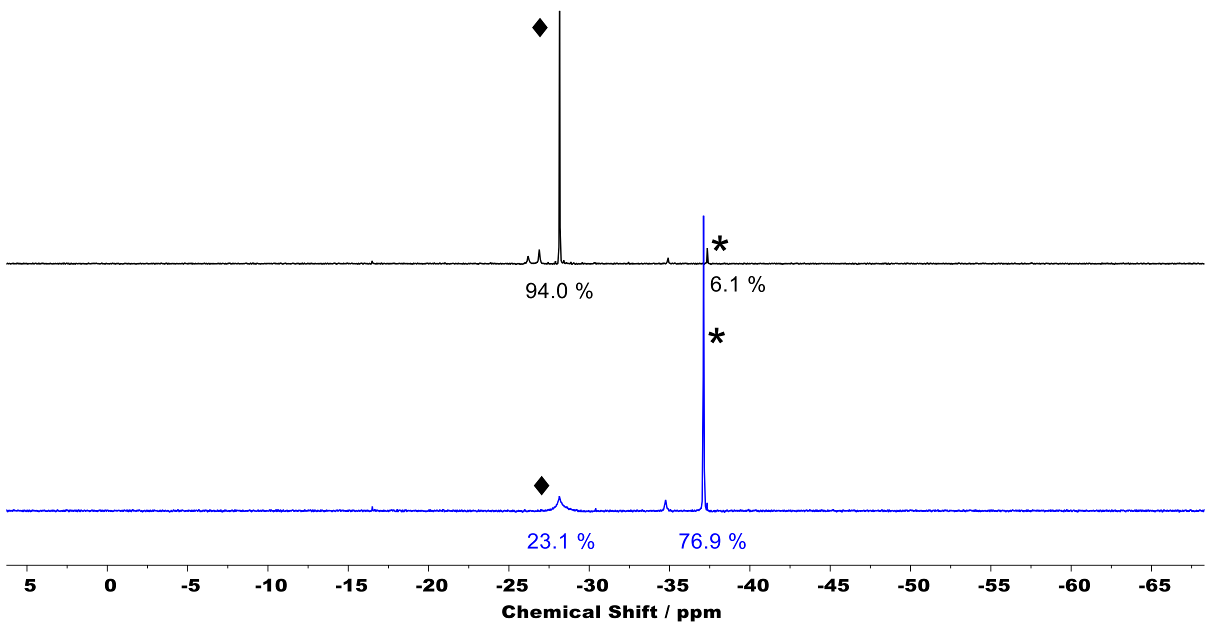

Fig. S60 ^{31}P NMR (162 MHz, toluene-d_8) spectra of the crude products from the mixture of FRP and 4-(trifluoromethyl)benzoate . Top: small molecular **1/3** and bottom: macromolecular **Poly-1/Poly-3**. ♦Converted protonated phosphine; * unreacted phosphine **3** or **Poly-3**.

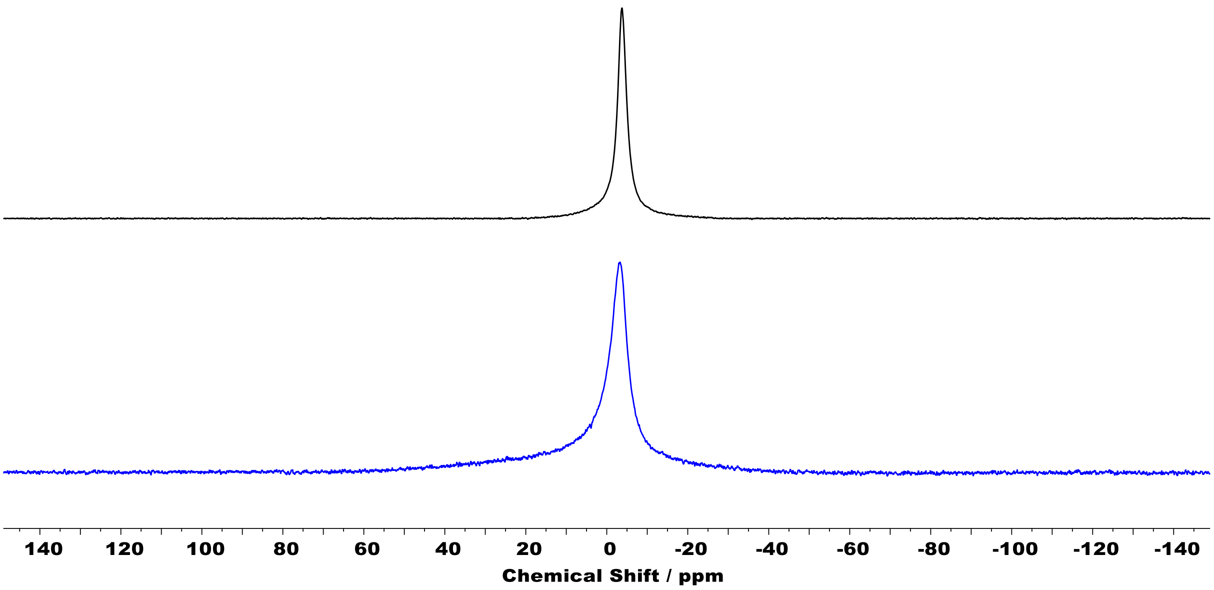

Fig. S61 ^{11}B NMR (128 MHz, toluene-d_8) spectra of the reaction mixture of **1**/**3**/styrene/4-(trifluoromethyl)benzoate (top) and **1**/**3**/4-(trifluoromethyl)benzoate (bottom).

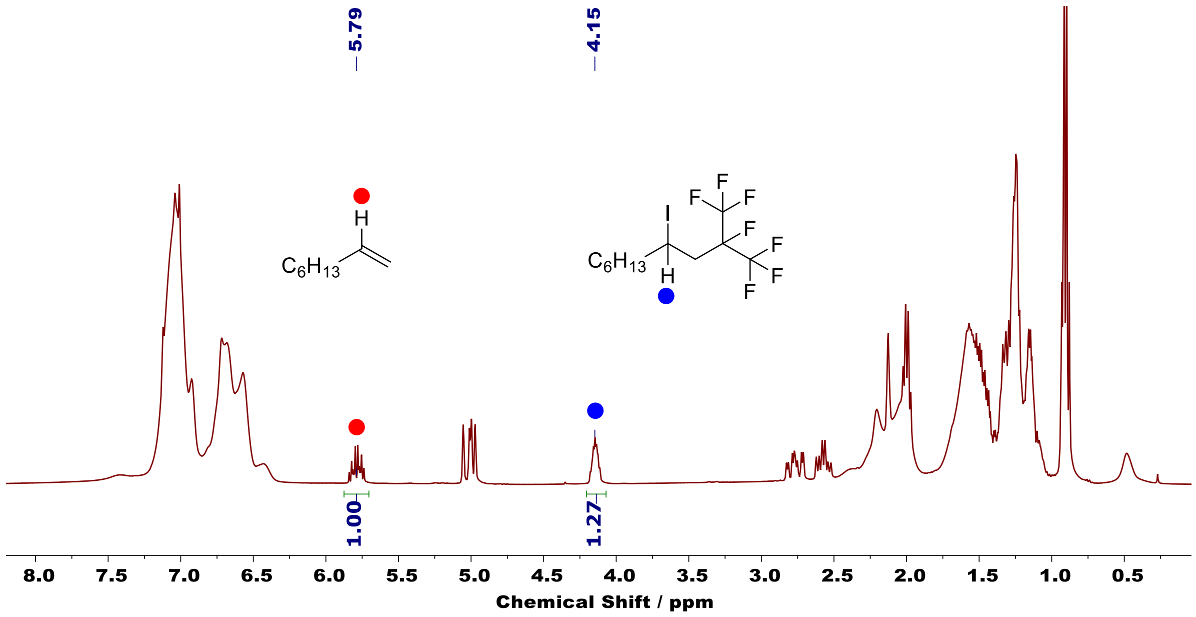

Fig.S62 An example to show to the determination of the conversion of perfluoroalkylation reaction using ^{1}H NMR spectroscopy.

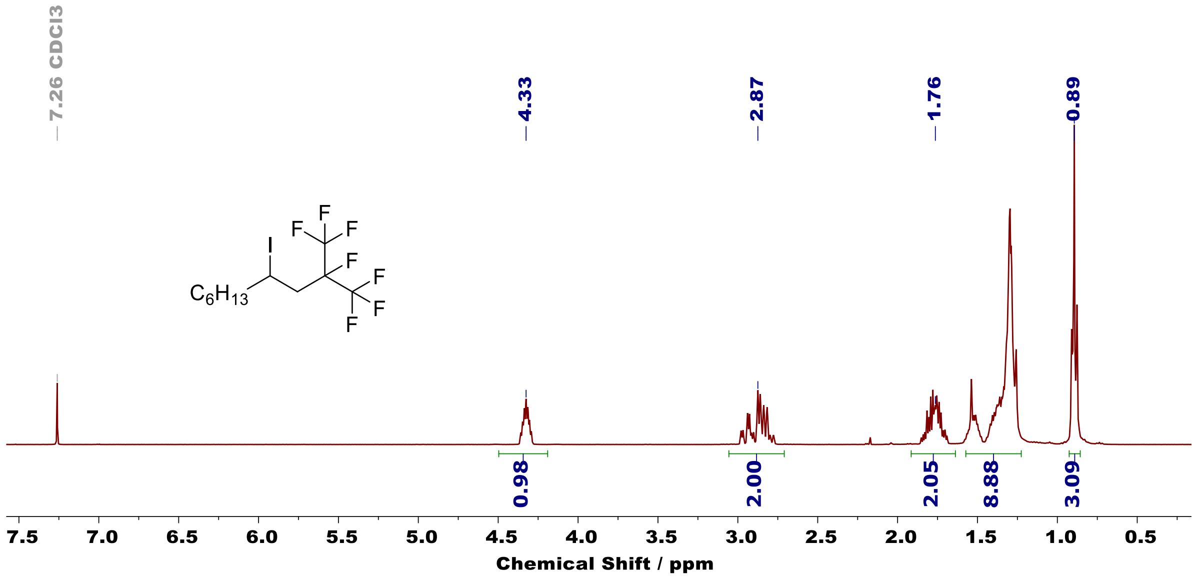

Fig.S63 ^{1}H NMR (400 MHz, $CDCl_3$) spectra of the purified perfluoroalkylation product.

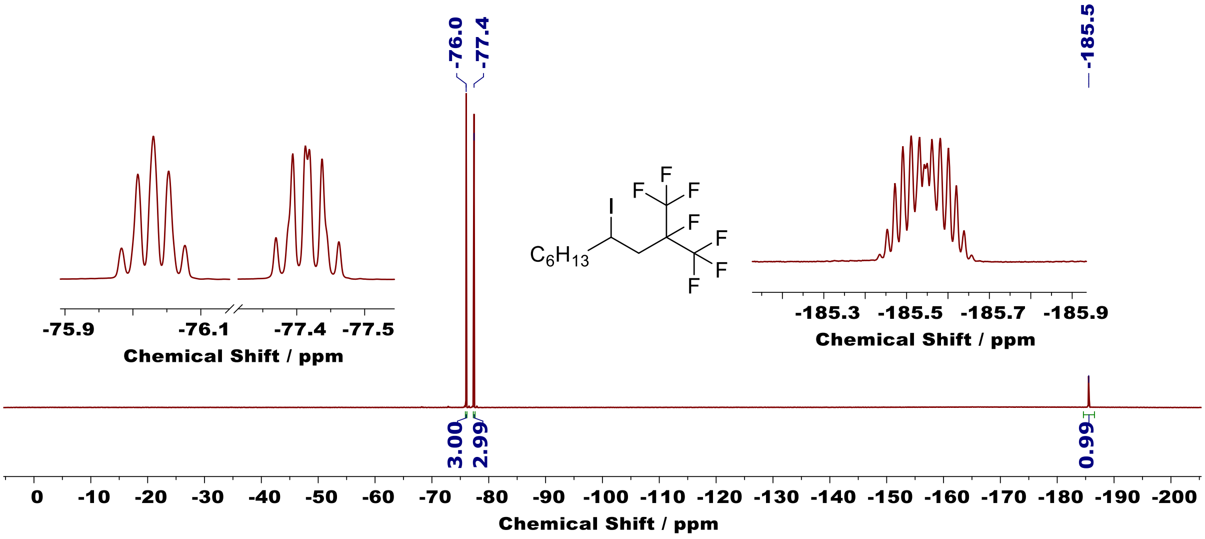

Fig.S64 ^{19}F NMR (376 MHz, $CDCl_3$) spectra of the purified perfluoroalkylation product.

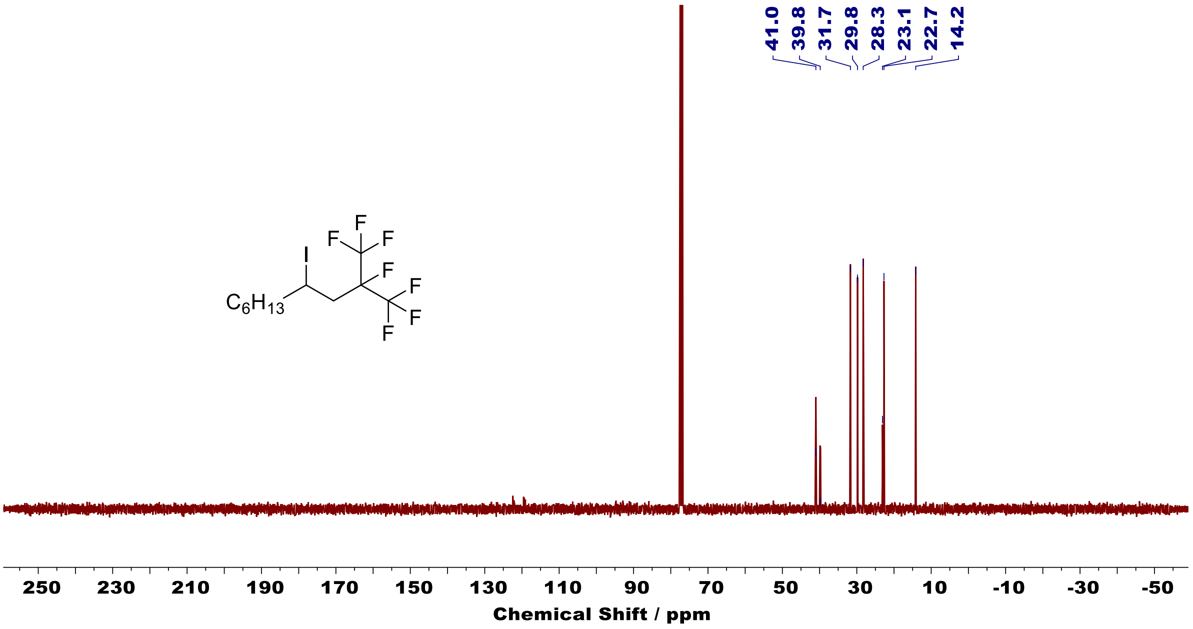

Fig.S65 ^{13}C NMR (101 MHz, $CDCl_3$) spectra of the purified perfluoroalkylation product.

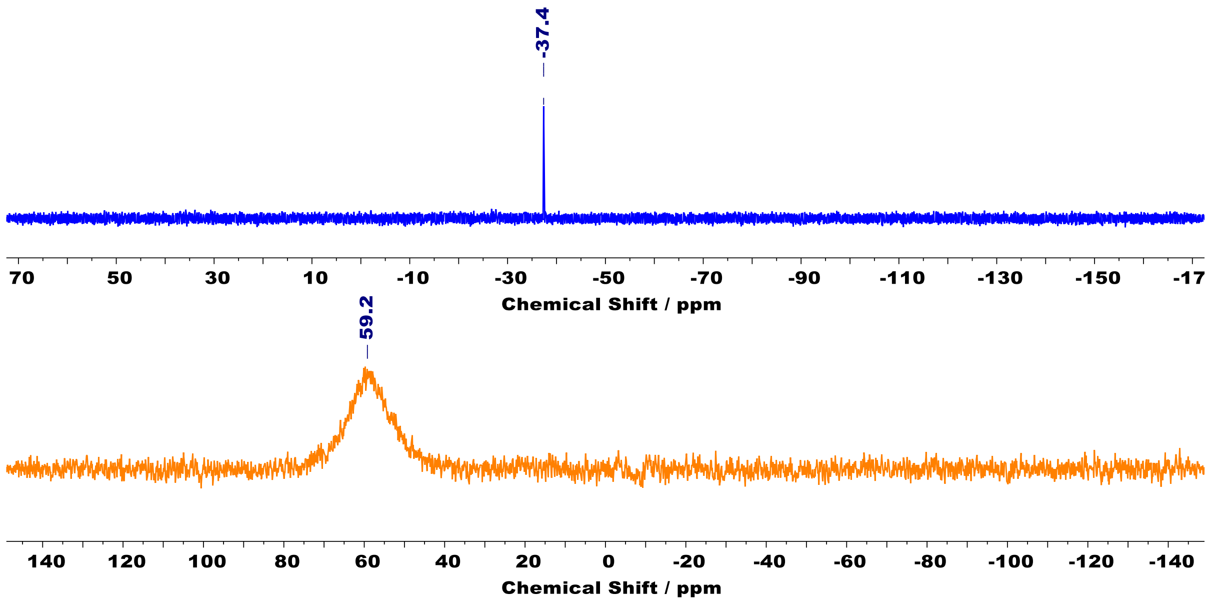

Fig.S66 Top: ^{31}P NMR (162 MHz, toluene-d_8) and bottom:^{11}B NMR (128 MHz, toluene-d_8) spectra of reaction mixture of **1**/**3**/1-octene/2-iodoheptafluoropropane.

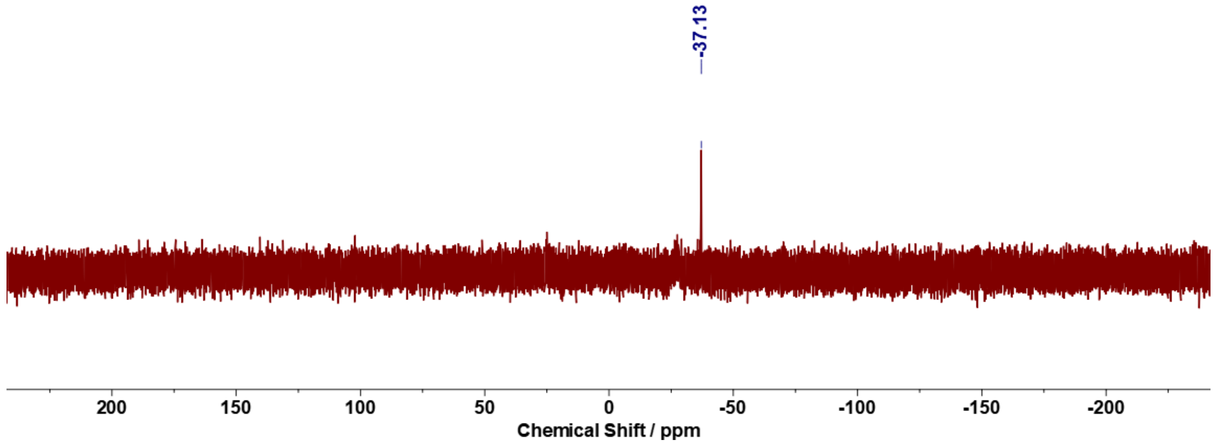

Fig.S67 ^{31}P NMR (162 MHz, toluene-d_8) of the recycled polymeric catalyst **Poly-1/Poly-3** after one photocatalytic cycle.

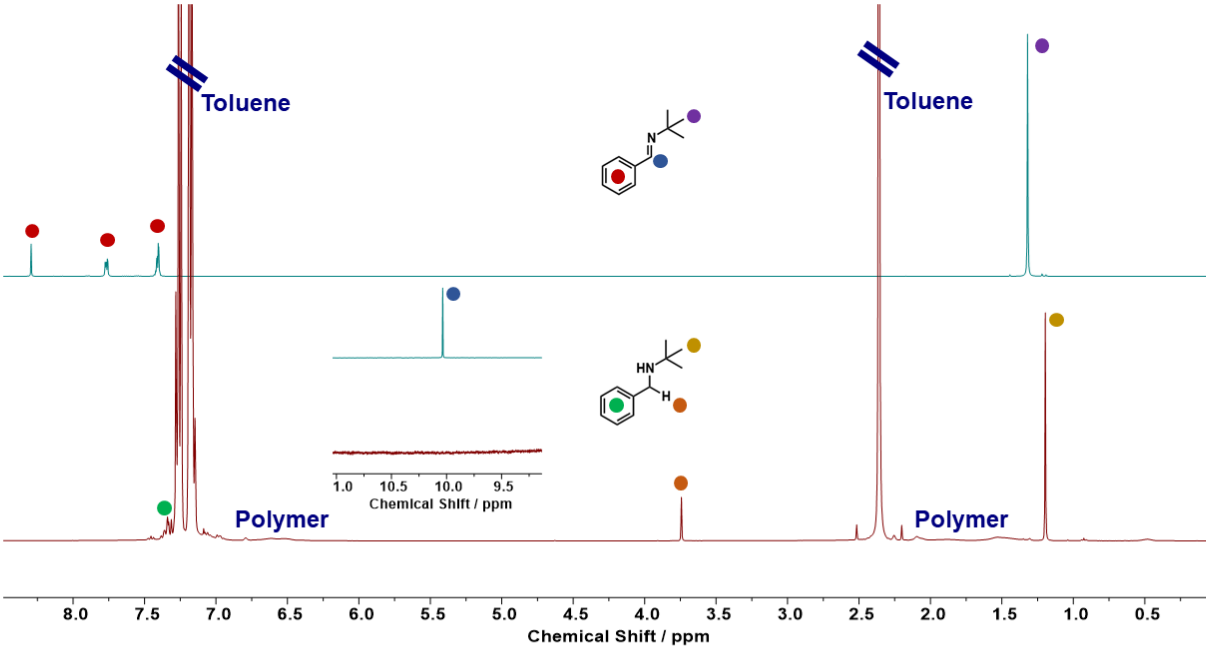

Fig.S68 ^{1}H NMR spectra (400 MHz, $CDCl_3$). **Top:** starting material N-benzylidene-*tert*-butylamine and **bottom:** crude mixture after the catalysed reaction. From the reaction crude, there's no peak corresponding to the starting material that can be observed (including the aromatic peaks and benzylidene proton), indicating a quantitative conversion achieved.

TGA, DSC and GPC Data

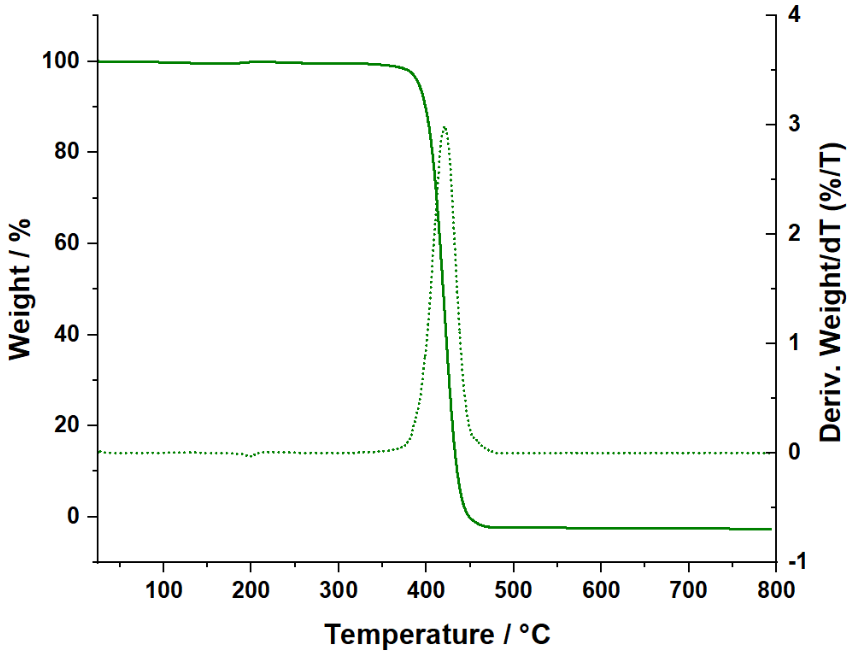

Fig.S69 TGA plot of **Poly-Si**. Solid line: Weight loss; Dotted line: first derivative of weight loss.

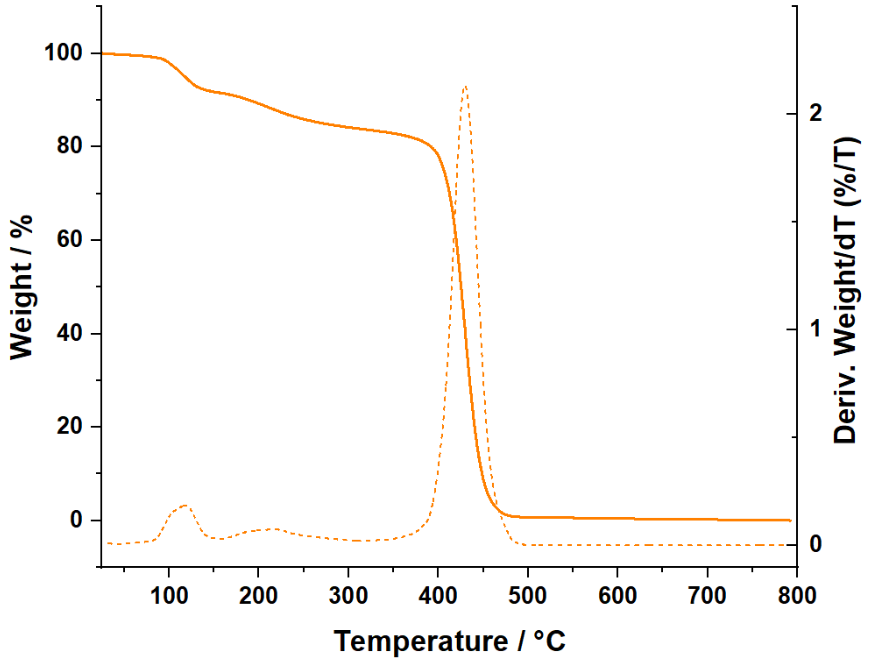

Fig.S70 TGA plot of **Poly-1**. Solid line: Weight loss; Dotted line: first derivative of weight loss.

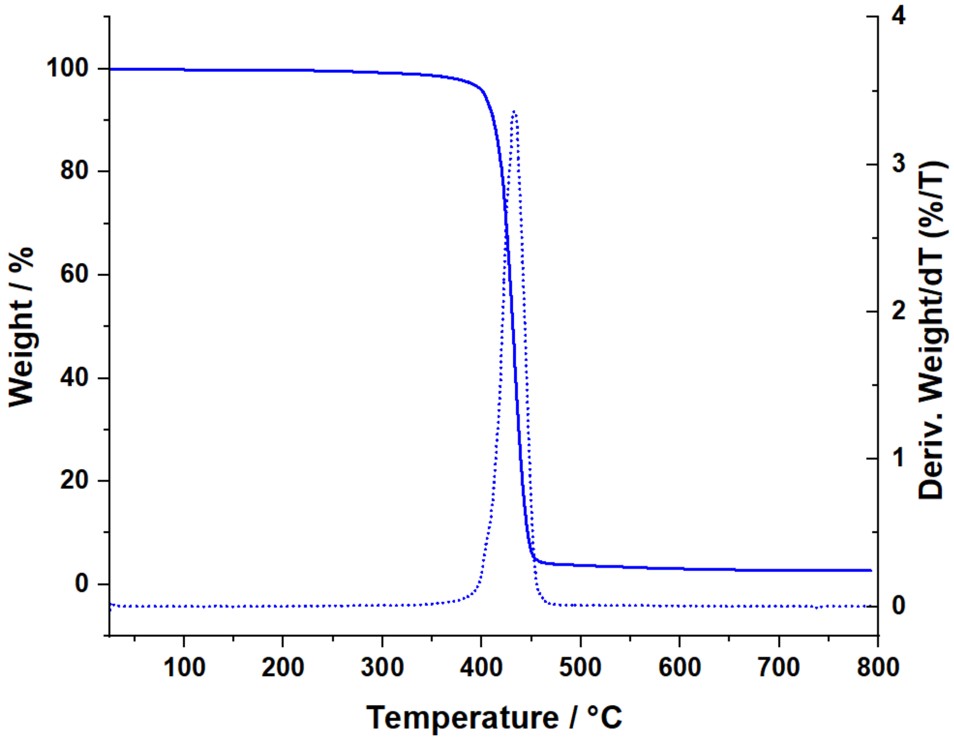

Fig.S71 TGA plot of **Poly-3**. Solid line: Weight loss; Dotted line: first derivative of weight loss.

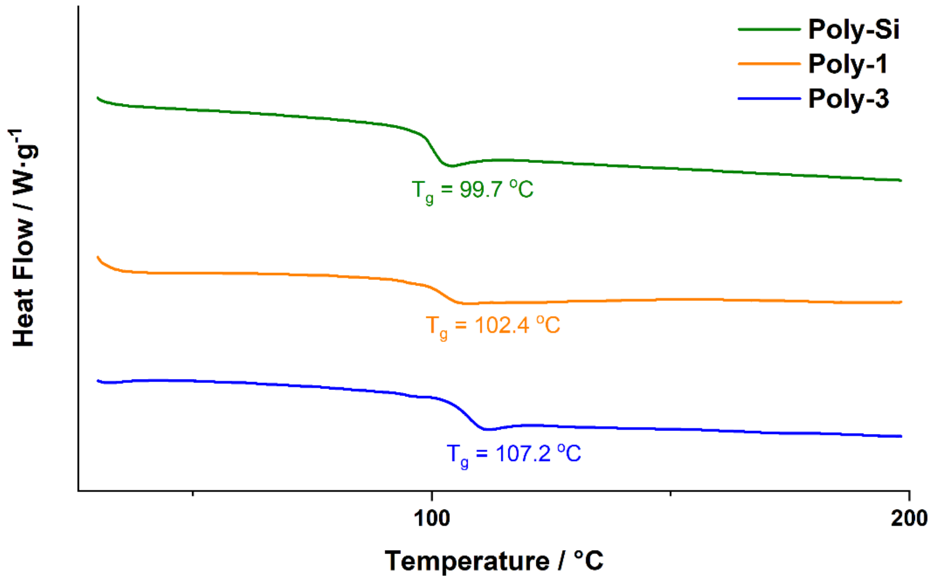

Fig.S72 Stacked DSC curves of **Poly-Si, Poly-1 and Poly-3**. Note that the unexpected low measured T_g value of **Poly-1** was due to the degradation during the measurements.

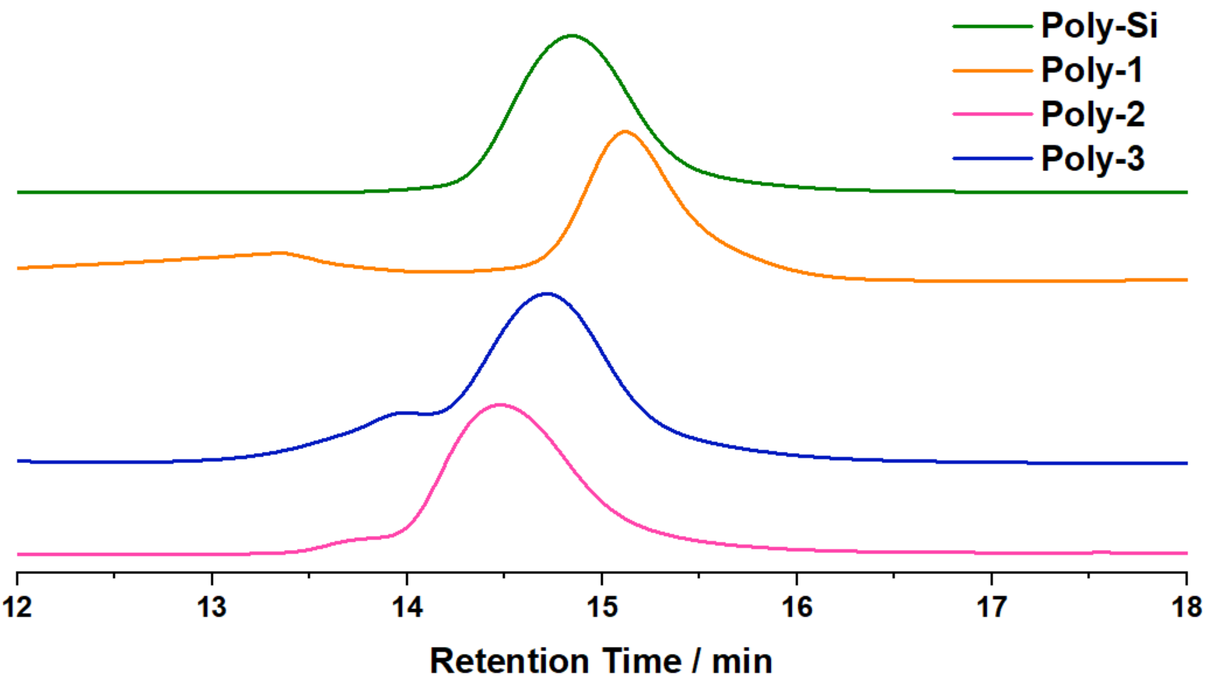

Fig.S73 Stacked GPC traces for **Poly-Si**, **Poly-1**, **Poly-2** and **Poly-3**. Note that after post-polymerisation modifications, **Poly-1** has longer retention time compared to its precursor **Poly-Si** due to the changed hydrodynamic radius of resultant copolymer, hence universal calibration was applied to determine the MW data. **Poly-1** also showed a larger cluster which correspond to high MWs, which also indicating its potential interactions to GPC column gels/impurities in solvent.

UV/Vis Spectroscopic Data

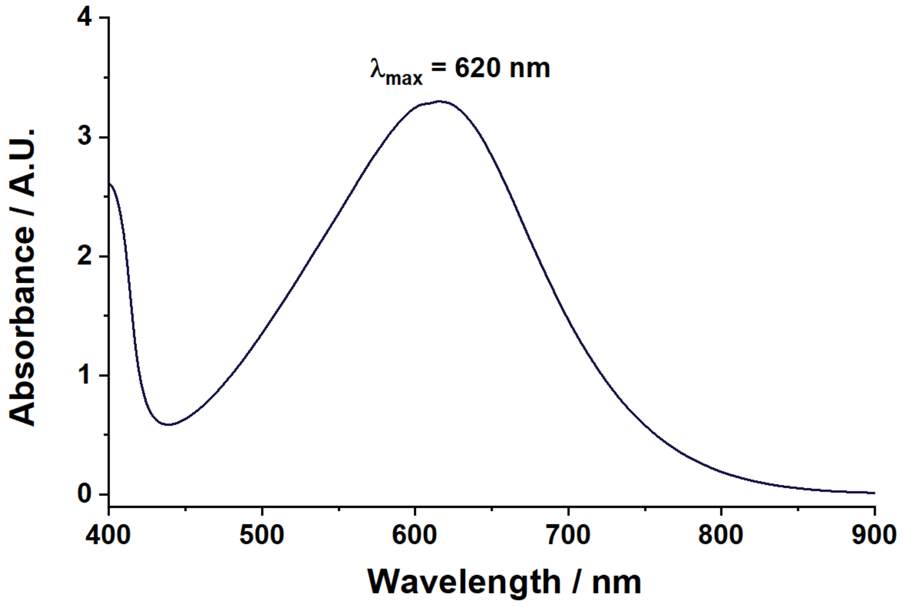

Fig.S74 UV/Vis spectrum of mixture of **1**, **3** and benzoyl peroxide.

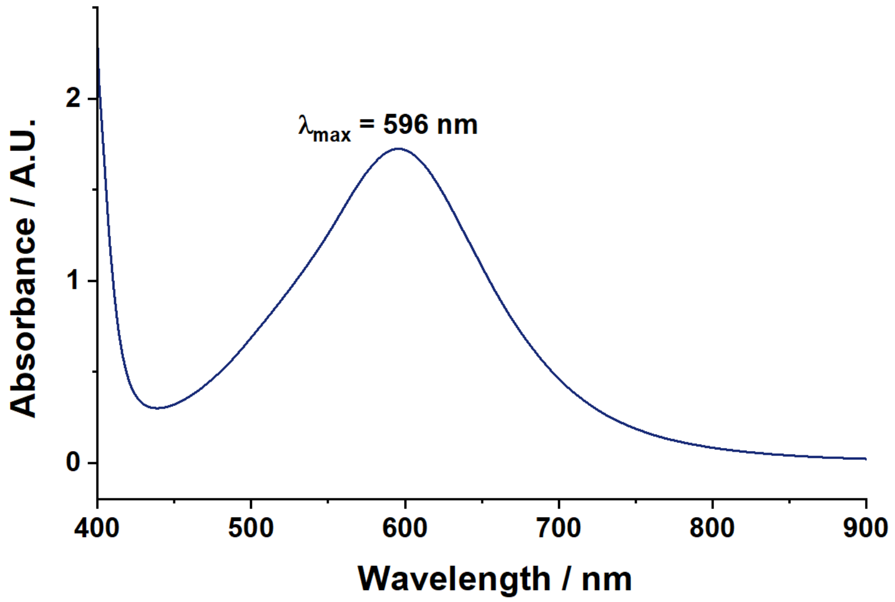

Fig.S75 UV/Vis spectrum of mixture of **Poly-1**, **Poly-3** and benzoyl peroxide.

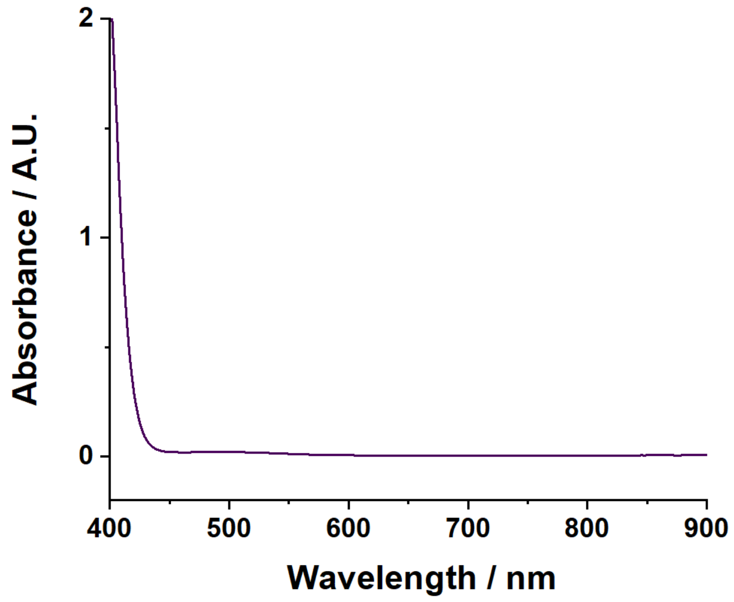

Fig.S76 UV/Vis spectrum of mixture of **1**, **2** and benzoyl peroxide.

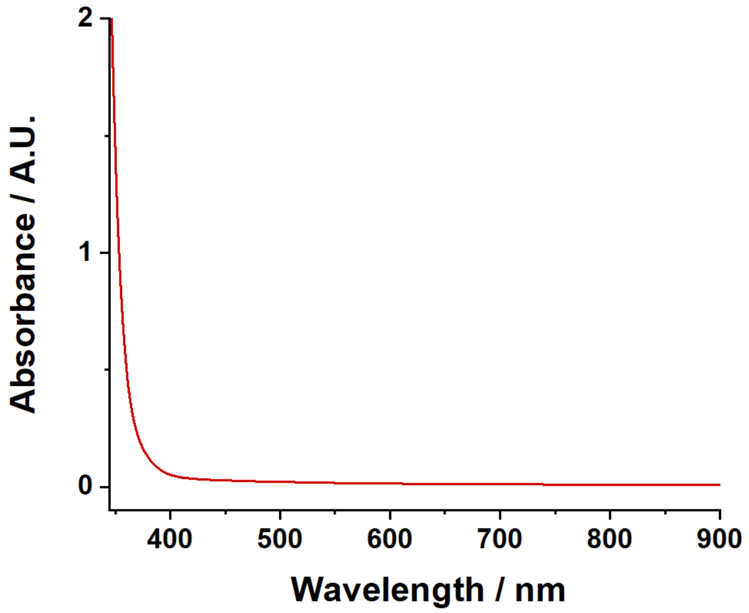

Fig.S77 UV/Vis spectrum of mixture of **Poly-1**, **Poly-2** and benzoyl peroxide.

EPR Spectroscopic Data

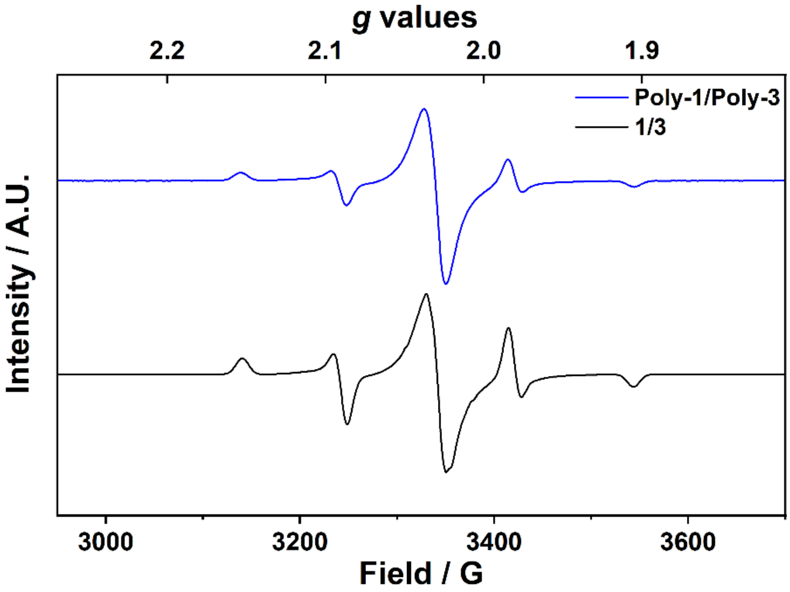

Fig.S78 Experimental data of anisotropic cw X-band EPR spectra at 20 K of **Poly-1/Poly-3** and **1/3** under blue LED (455 nm) irradiation.

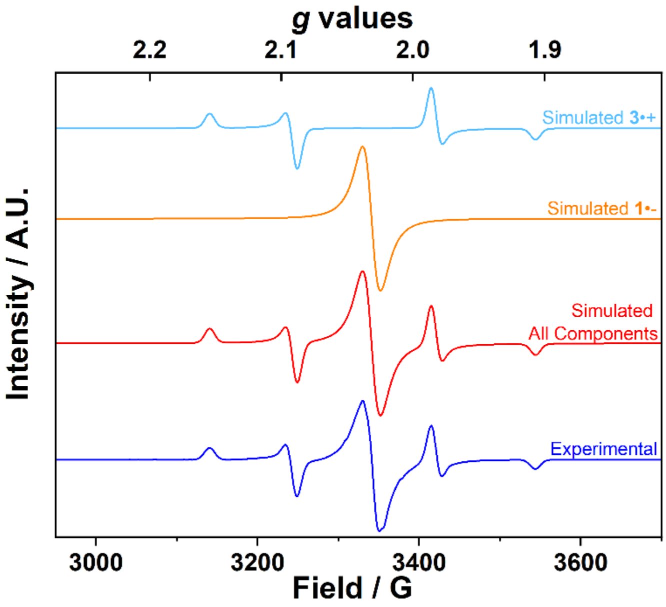

Fig.S79 Simulation and Experimental data of anisotropic cw X-band EPR spectra of **1/3** at 20K under blue LED (455 nm) irradiation.

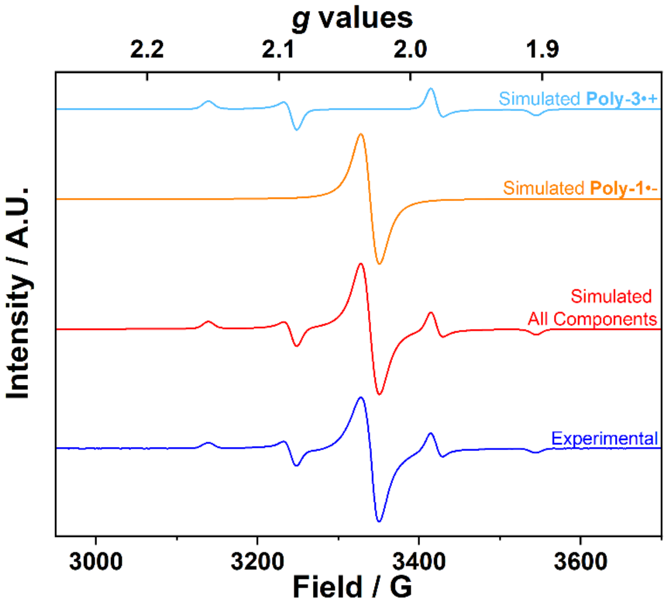

Fig.S80 Simulation and Experimental data of anisotropic cw X-band EPR spectra of **Poly-1/Poly-3** at 20K under blue LED (455 nm) irradiation.

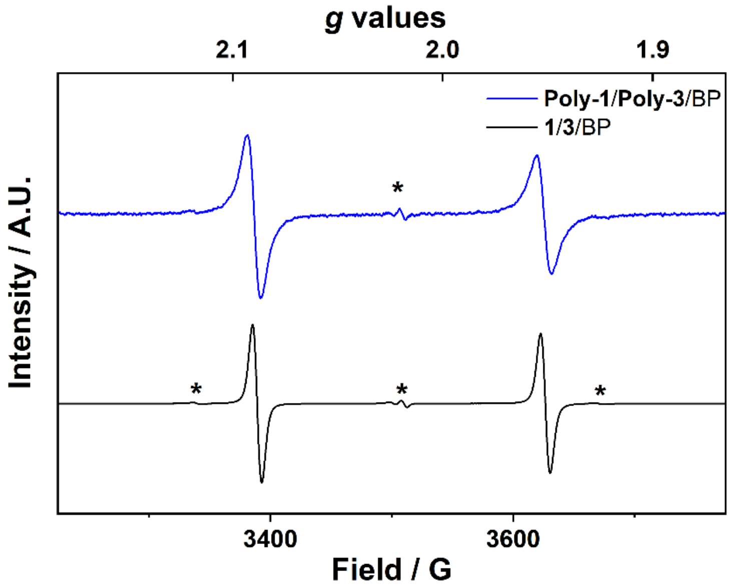

Fig.S81 Experimental data of isotropic cw X-band EPR spectra of **Poly-1/Poly-3**/BP and **1/3**/BP. * Radicals generated as side products during the reaction.

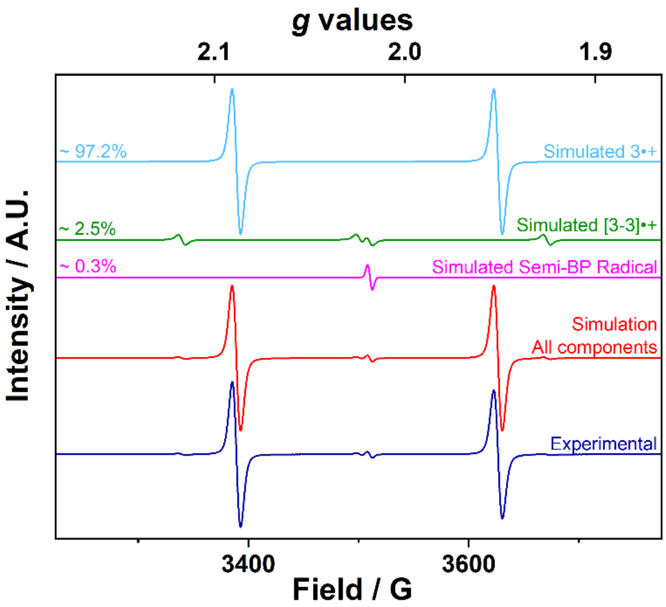

Fig.S82 Simulation and Experimental data of isotropic cw X-band EPR spectra of **1/3**/BP.

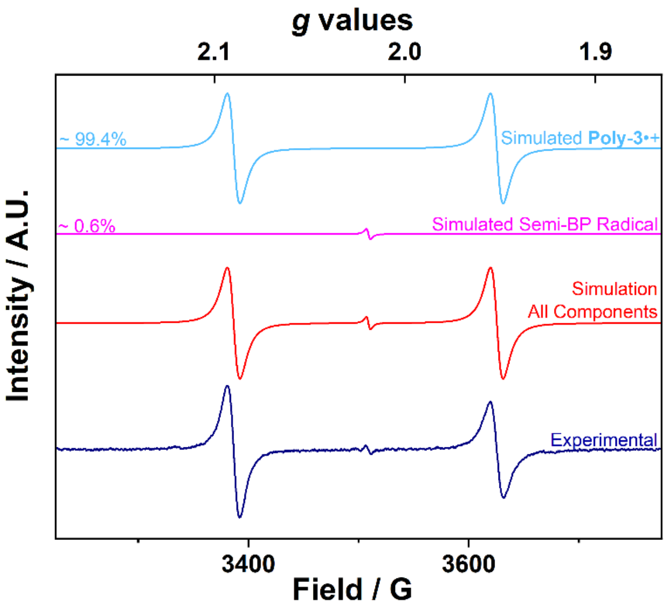

Fig.S83 Simulation and Experimental data of isotropic cw X-band EPR spectra of **Poly-1/Poly-3**/BP.

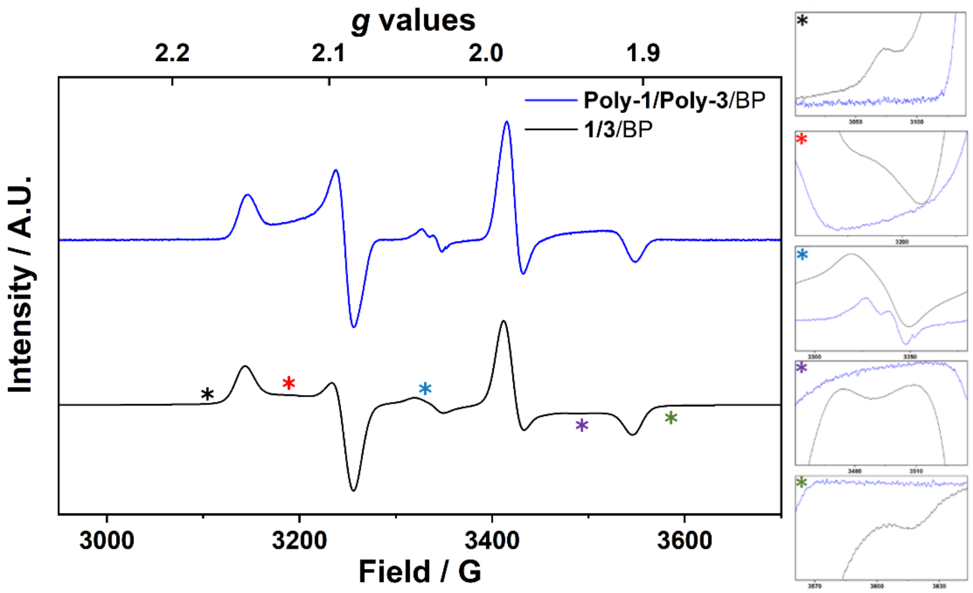

Fig.S84 Experimental data of anisotropic cw X-band EPR spectra at 20 K of **Poly-1/Poly-3**/BP and **1/3**/BP. * Radicals generated as side products during the reaction.

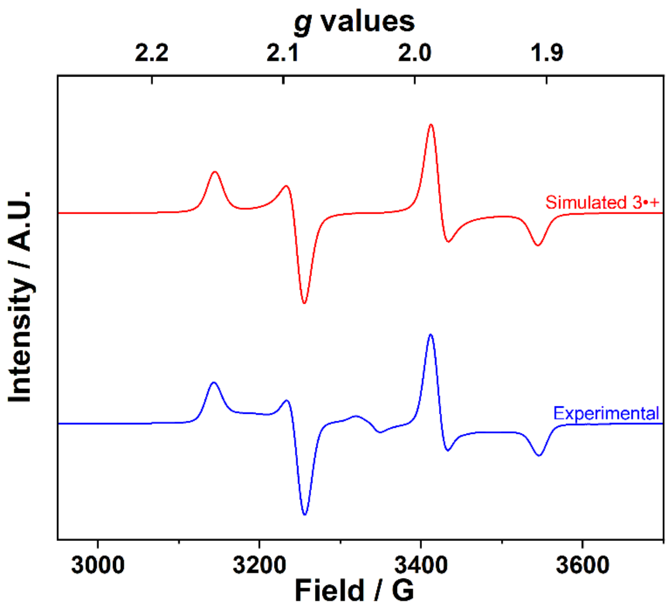

Fig.S85 Simulation and experimental data of anisotropic cw X-band EPR spectra of **1/3**/BP at 20 K.

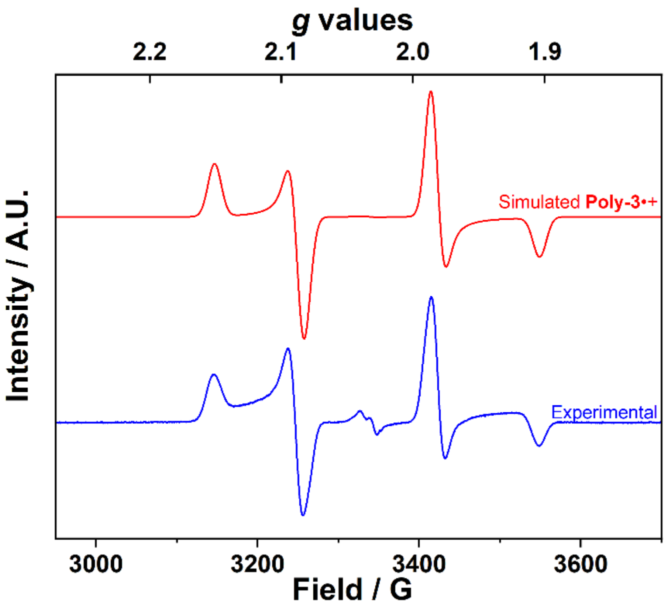

Fig.S86 Simulation and experimental data of anisotropic cw X-band EPR spectra of **Poly-1/Poly-3**/BP at 20 K.

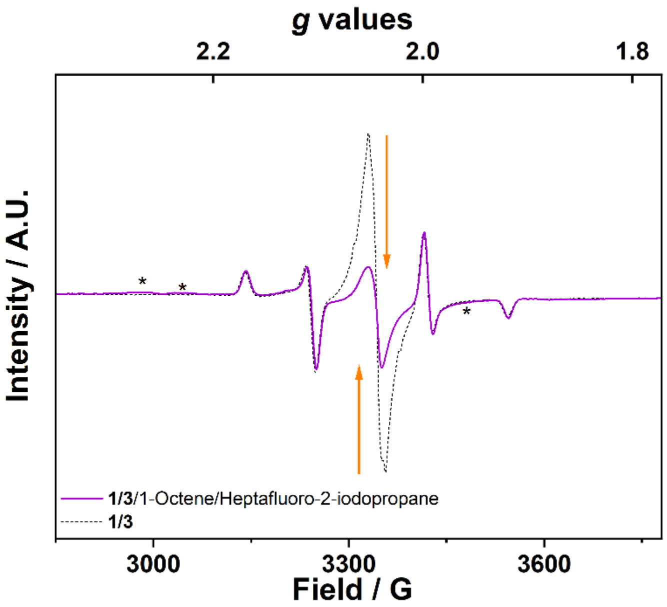

Fig.S87 Anisotropic cw X-band EPR spectra at 20 K of the mixture of **1/3**/1-Octene/ Heptafluoro-2-iodopropane comparing to the FRP without substrates. * New unknown radical species generated during LED (455 nm) irradiation.

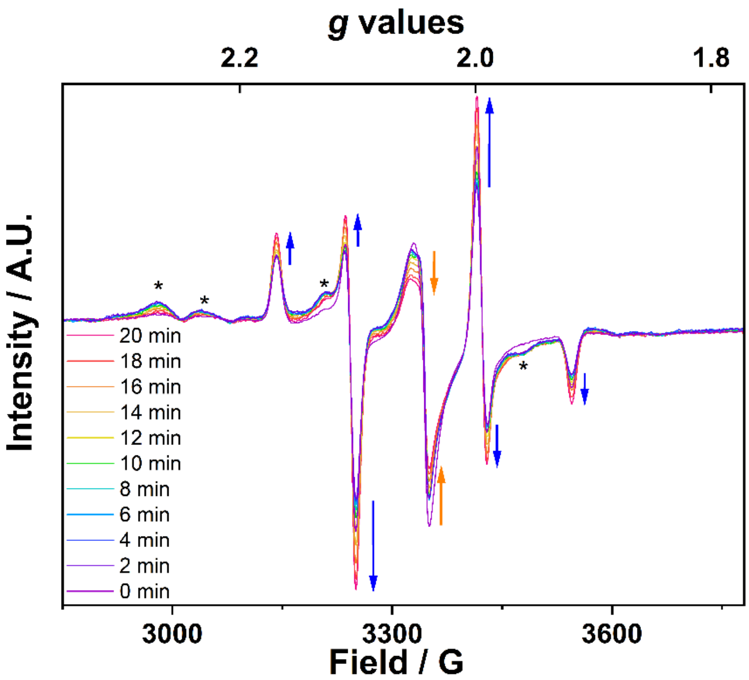

Fig.S88 Anisotropic cw X-band EPR spectra at 20 K of the mixture of **1/3**/1-Ocetene/ Heptafluoro-2-iodopropane after turning off the LED irradiation. * Unknown radical species generated during LED (455 nm) irradiation.

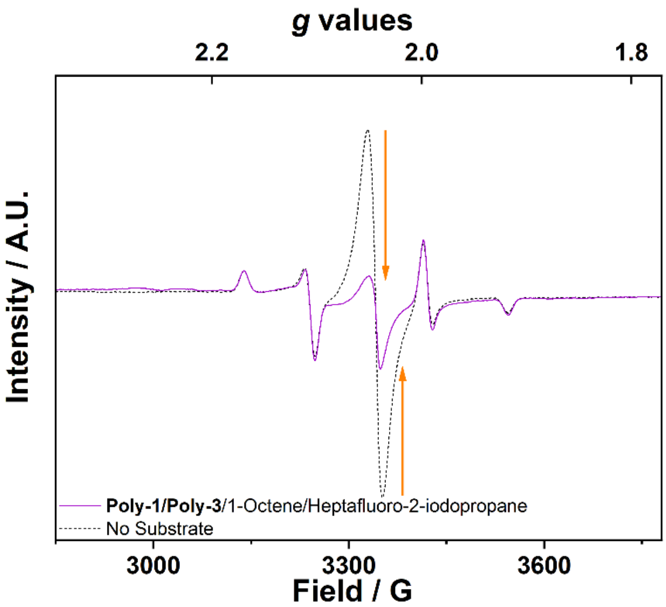

Fig.S89 Anisotropic cw X-band EPR spectra at 20 K of the mixture of **Poly-1/Poly-3**/1-Ocetene/ Heptafluoro-2-iodopropane comparing to the poly(FRP) without substrates. * New unknown radical species generated during LED (455 nm) irradiation.

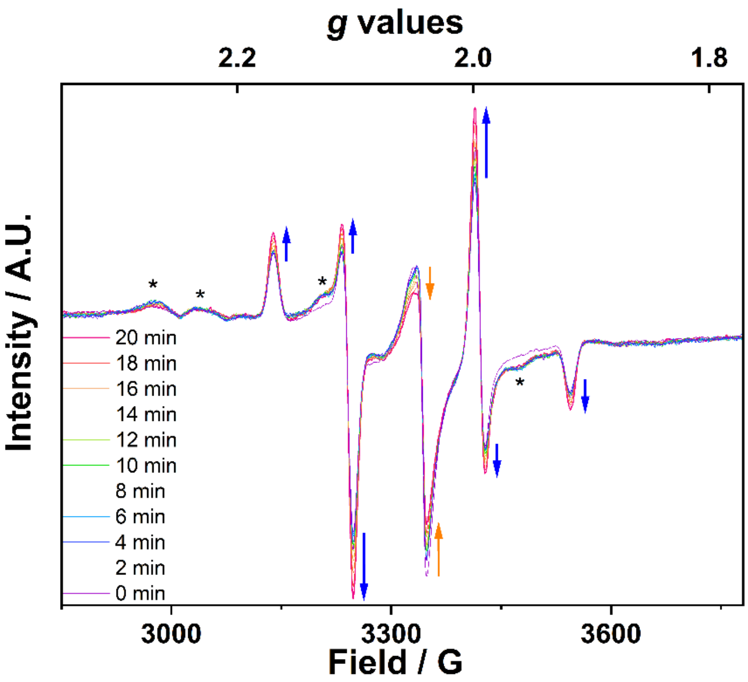

Fig.S90 Anisotropic cw X-band EPR spectra at 20 K of the mixture of **Poly-1/Poly-3**/1-Ocetene/ Heptafluoro-2-iodopropane after turning off the LED irradiation. * Unknown radical species generated during LED (455 nm) irradiation.

Reference

1. Wang, M.; Nudelman, F.; Matthes, R. R.; Shaver, M. P., Frustrated Lewis Pair Polymers as Responsive Self-Healing Gels. *J. Am. Chem. Soc.* **2017,** *139* (40), 14232-14236.
2. Yolsal, U.; Wang, M.; Royer, J. R.; Shaver, M. P., Rheological Characterization of Polymeric Frustrated Lewis Pair Networks. *Macromolecules* **2019,** *52* (9), 3417-3425.
3. Roesler, R.; Har, B. J. N.; Piers, W. E., Synthesis and Characterization of (Perfluoroaryl)borane-Functionalized Carbosilane Dendrimers and Their Use as Lewis Acid Catalysts for the Hydrosilation of Acetophenone. *Organometallics* **2002,** *21* (21), 4300-4302.
4. Sivaev, I. B.; Bregadze, V. I., Lewis acidity of boron compounds. *Coord. Chem. Rev.* **2014,** *270*, 75-88.
5. Behrends, I.; Bähr, S.; Czekelius, C., Perfluoroalkylation of Alkenes by Frustrated Lewis Pairs. Chemistry – A European Journal **2016**, 22 (48), 17177-17181.
